# Discovery of a small molecule secreted clusterin enhancer that improves memory in Alzheimer’s disease mice

**DOI:** 10.1038/s44386-025-00009-2

**Published:** 2025-05-02

**Authors:** Whitaker Cohn, Jesus Campagna, Dongwook Wi, Jessica T. Lee, Sahiba Beniwal, Gazmend Elezi, Chunni Zhu, Barbara Jagodzinska, Julian Whitelegge, Robert Damoiseaux, Varghese John

**Affiliations:** 1 The Drug Discovery Lab, Mary S. Easton Center for Alzheimer’s Disease Research, Department of Neurology, David Geffen School of Medicine, 710 Westwood Plaza, University of California Los Angeles, Los Angeles, USA; 2 Pasarow Mass Spectrometry Laboratory, Jane and Terry Semel Institute for Neuroscience and Human Behavior, David Geffen School of Medicine, 760 Westwood Plaza, University of California Los Angeles, Los Angeles, USA; 3 Department of Molecular and Medical Pharmacology, 650 Charles E. Young Drive, University of California Los Angeles, Los Angeles, USA

**Keywords:** Biomarkers, Drug screening, Medicinal chemistry, Target validation

## Abstract

Despite substantial research and drug discovery efforts, Alzheimer’s Disease (AD) remains the sixth leading cause of death in the United States, underscoring the urgent need for novel therapeutic targets. A mutation in the clusterin (CLU) gene that hinders expression of the cyto-protective secreted isoform of clusterin (sCLU) that affects the aggregation and clearance of two key proteins implicated in AD, Aβ and tau, is the third most significant genetic risk factor for late-onset AD. Here, we present findings from our drug discovery program to identify small molecules that enhance sCLU levels and assess their impact on AD pathology and cognition in a murine model of AD. A high-throughput screening campaign identified two classes of epigenetic modulators that increase sCLU levels with subsequent medicinal chemistry efforts leading to bromodomain and extra-terminal (BET) inhibitor new chemical entities (NCEs) with enhanced potency, drug-like properties, and oral brain bioavailability. The lead candidate NCE, DDL-357, increased brain sCLU in the murine ApoE4TR-5XFAD model of AD in a subchronic study. In a follow-up chronic study in the murine 3xTg-AD model, DDL-357 reduced phospho-tau in brain and led to improvements in mouse performance and memory in the Barnes maze testing paradigm. Proteomic analysis of brain tissue from both AD models revealed changes in proteins involved in mitochondrial function and synaptic plasticity. These findings reveal the potential of sCLU enhancement as a target for therapeutic development in AD and support the continued development of the preclinical lead candidate.

## Introduction

Alzheimer’s Disease (AD), the most common form of age-related dementia, is characterized by the gradual accumulation of amyloid-β (Aβ) plaques and tau neurofibrillary tangles in the brain, leading to significant inflammation, synaptic injury, and progressive neuronal degeneration^1^. Despite extensive research and drug discovery efforts, AD remains the sixth leading cause of death in the United States and there are no truly effective treatment options^2^. The first therapeutics approved for use in AD, acetylcholinesterase inhibitors and N-methyl D-aspartyl (NMDA) receptor antagonists, only provide temporary symptomatic relief and the more recently approved anti-amyloid monoclonal antibodies only marginally slow cognitive decline^3^. The limited benefit of these therapeutics underscores the necessity for identifying new targets for therapeutic development in AD. To achieve meaningful efficacy, it is likely multi-functional drug candidates that target primary disease mechanisms as well as promote key neuroprotective functions that halt the progression of this devastating disease will be necessary.

Genome-wide association studies (GWAS) indicate the third strongest genetic risk factor for late-onset Alzheimer’s disease (LOAD) is a single nucleotide polymorphism (SNP) in a gene that encodes for clusterin (CLU), a multifunctional chaperone protein directly involved in a wide range of AD-associated biological processes, including Aβ and tau metabolism, lipid transport, immune modulation, oxidative stress, and apoptosis^4^. Seven CLU gene variants have been associated with AD, and individuals carrying the C allele of the rs11136000 SNP show increased Aβ and tau deposition, accelerated cognitive decline, and decreased expression of the secreted clusterin isoform (sCLU) compared to other isoforms^5,6^. Consequently, extensive research on CLU in different disease states, including neurodegeneration, cardiovascular disease, and cancer, has led to the prevailing hypothesis that sCLU promotes cytoprotection, while intracellular isoforms (nCLU) have pro-apoptotic effects^4^.

**Fig. 1:**
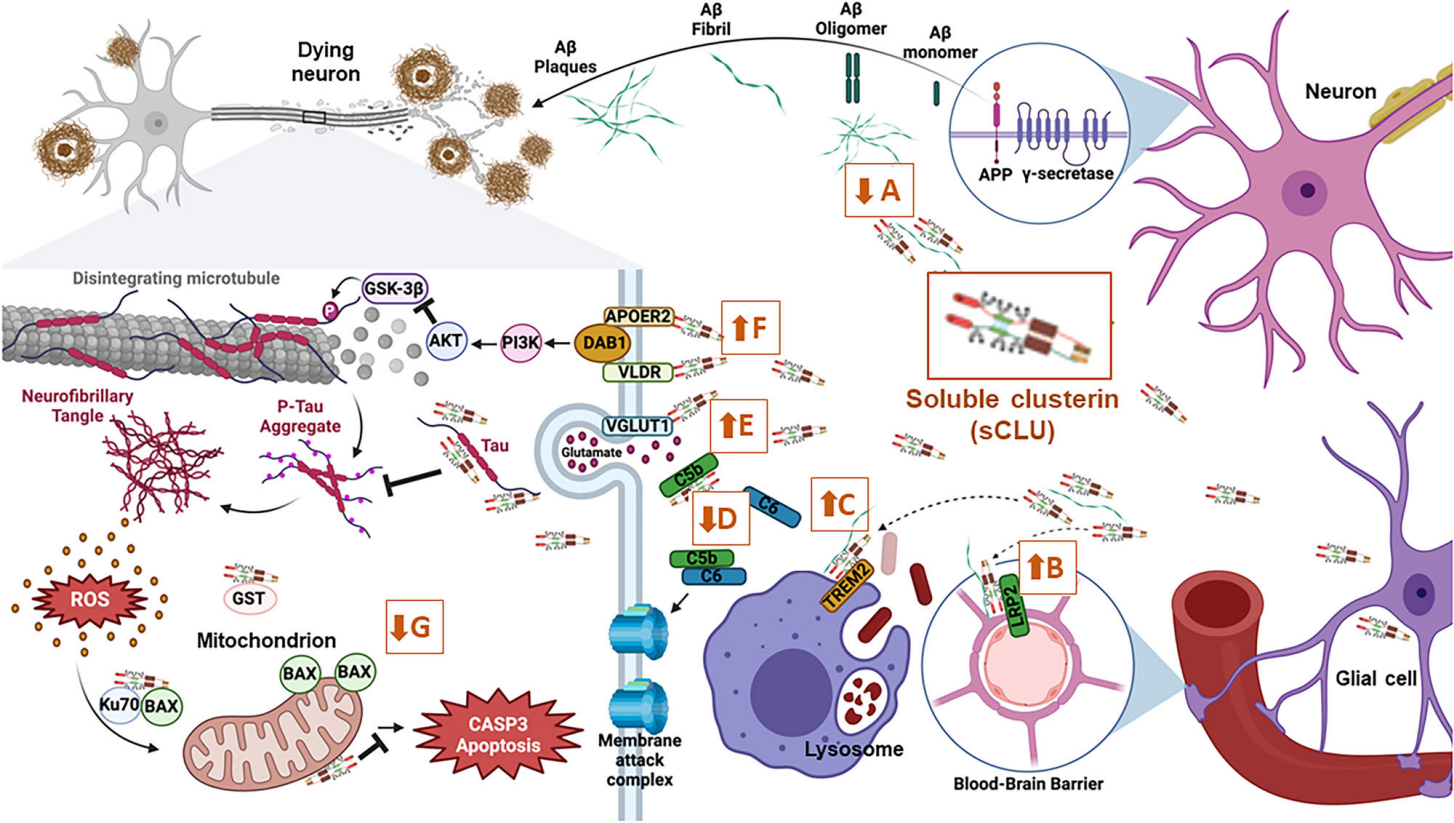
Molecular mechanisms of secreted clusterin for preventing Alzheimer’s disease. Secreted clusterin (sCLU; outlined in orange): **A** binds and prevents the aggregation of Aβ; **B** interacts with LRP2 to transport Aβ species across the blood-brain barrier into the periphery; **C** interacts with TREM2 to transport Aβ into glial cells for lysosomal degradation; **D** prevents assembly of the membrane attack complex and associated apoptosis; **E** interacts with VGLUT1 to stimulate excitatory neurotransmission; **F** interacts with APOER2 and VLDR to induce reelin signaling, which decreases tau phosphorylation by inhibiting GSK3β; and **G** prevents oxidative stress and associated CASP3-mediated apoptosis. Illustrations were created using Biorender.com.

The most significant protective function of sCLU is its role in regulating protein homeostasis within the central nervous system (CNS). Research shows that sCLU binds and prevents aggregation of disease-relevant toxic, misfolded proteins, including α-synuclein, TDP-43, tau and Aβ (Fig. 1A)^7–10^. sCLU is a crucial mediator of Aβ clearance from the brain due to its high affinity for Aβ and endocytic receptors like LRP2 and TREM2, facilitating the transport of toxic Aβ species across the blood-brain barrier (BBB) or into glial cells for lysosomal degradation (Fig. 1B and C)^11,12^. sCLU prevents assembly of the membrane attack complex and associated apoptosis, interacts with VGLUT1 to stimulate excitatory neurotransmission, and binds to ApoER2 and VLDR, activating signaling pathways such as the reelin pathway that can reduce tau phosphorylation (p-tau) (Fig. 1D, E, and F)^13,14^. sCLU also protects against oxidative stress, mitochondrial dysfunction, harmful immunological changes, and synaptic deficits, all of which are factors that contribute to AD progression (Fig. 1G)^15–19^. This suggests enhancement of sCLU is a rationale target for novel therapeutic development in AD. In support of this, peripheral administration of recombinant sCLU in an AD mouse model was observed to reduce Aβ accumulation, neuronal loss and levels of pro-inflammatory markers^20^.

Here, we report our discovery of a sCLU enhancer lead candidate from our comprehensive drug discovery effort aimed at identifying potent, brain-permeable small molecules that enhance brain sCLU levels and suppress AD pathology, while improving memory function, and neuroprotective proteins in a murine models of AD. High-throughput screening (HTS) was first employed to identify small molecule compounds that enhance sCLU. Following confirmation and prioritization of HTS hits, we conducted exploratory medicinal chemistry on a known, potent bromodomain extraterminal (BET) protein inhibitor to design new chemical entity (NCE) sCLU enhancers. Utilizing in-vitro absorption, distribution, metabolism, excretion, and toxicity (ADME-T) assays, as well as in-vivo pharmacokinetics (PK) analyzes, we assessed drug-like properties and oral brain permeability of analogs to guide lead selection for further preclinical efficacy testing in two AD mouse models. The lead candidate, DDL-357, that possessed good drug-like characteristics, was found to increase sCLU in brain in the ApoE4TR-5XFAD murine model of AD, and both decrease phospho-tau (p-tau) and improve memory in the 3xTg-AD murine model of AD.

### HTS identifies HDAC and BET inhibitors that increase sCLU levels

**Fig. 2:**
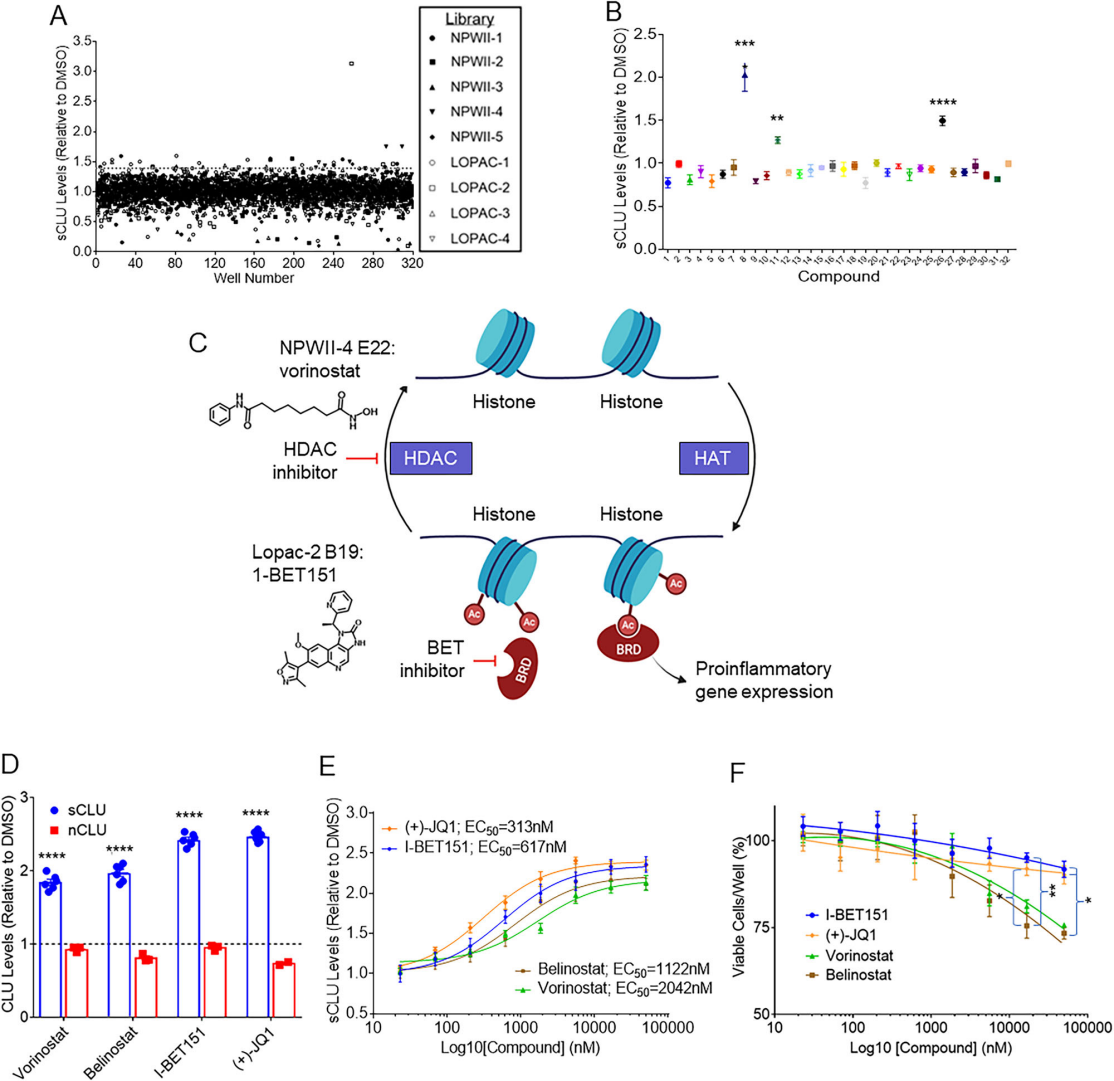
HTS identification of HDAC and BET inhibitors that increase sCLU levels in vitro. **A** Scattergraph of sCLU levels from HTS of a subset of UCLA compound library at a concentration of 5 µM in U-87 MG glioblastoma cells. The UCLA module name is listed at right. **B** Three of 32 HTS hits significantly increase sCLU levels in U-87 MG cells in re-testing (n = 3 per compound). **C** Schematic showing the molecular relationship between HDAC inhibitor, vorinostat, and BET inhibitor, I-BET151in transcriptional regulation. **D** sCLU and nCLU levels following treatment U-87 MG cells with HDAC inhibitors vorinostat and belinostat or BET inhibitors I-BET151 and (+)-JQ1 at 5 µM (*n* = 6 for sCLU and *n* = 3 for nCLU). Dashed line at 1 represents DMSO control. **E** Dose-response curves showing sCLU levels and calculated EC_50_ values for vorinostat, belinostat, I-BET151, and (+)-JQ1 (*n* = 6). **F** Dose-response cytotoxicity testing in U-87MG cells following treatment with vorinostat, belinostat, I-BET151, and (+)-JQ1 (*n* = 4). All results graphed as mean ± SEM. All statistics were performed using one-way ANOVA (*p ≤ 0.05; **p < 0.01; ***p < 0.001; ****p < 0.0001). Illustrations created with Biorender.com.

Our efforts to identify potent, brain-permeable small molecules that increase sCLU levels started with a HTS campaign using a customized AlphaLISA immunoassay designed to quantify sCLU secreted from human U-87 MG glioblastoma cells. Modules from the UCLA compound library were screened at a concentration of 5 µM (Fig. 2A) and three of the thirty-two hit compounds were retested in triplicate and confirmed to significantly increase sCLU levels relative to DMSO controls (Fig. 2B). These validated hits included two HDAC inhibitors, vorinostat and piperlongumine, as well as one BET inhibitor, I-BET151. Notably, all three compounds inhibit epigenetic protein families that recognize acetyl groups on lysine residues (Fig. 2C). HDACs regulate transcription by removing acetyl groups from histones and transcription factors and are linked to neurodegenerative diseases, including AD^21^. HDAC inhibition has been described as a promising therapeutic strategy offering neuroprotection by reversing hypoacetylation in AD, preventing Aβ-induced tau hyperphosphorylation, and promoting genes related to synaptic plasticity, learning, and memory^21^. The HDAC inhibitor vorinostat has been evaluated in phase 1 clinical trials^22^. Similarly, BET proteins are recognized as master transcriptional regulators, using two acetyl lysine-recognizing bromodomains to recruit transcription factors and coactivators to target gene sites^23^. BET inhibition has also demonstrated beneficial effects in AD mouse models, reducing neuroinflammation and tau phosphorylation, and promoting brain plasticity and cognitive function^23–26^. The BET inhibitor apabetalone has been associated with improved cognition in clinical testing^27^.

Next, we conducted testing of the commercially available structurally diverse HDAC (vorinostat and belinostat) and BET (I-BET151, and (+)-JQ1) inhibitors in U-87 MG cells and determined that all increased sCLU while not affecting nCLU levels (Fig. 2D). The enantiomer (−)-JQ1) did not increase sCLU (Supplementary Fig. S1A). Dose-response analyzes revealed that, overall, BET inhibitors were more potent sCLU enhancers than HDAC inhibitors, based on EC50s (Fig. 2E). I-BET151 and (+)-JQ1 both demonstrated nanomolar potency, increasing sCLU with EC50 values of 617 nM and 313 nM, respectively. In contrast, vorinostat and belinostat had higher EC50 values of 2042 nM and 1122 nM, respectively. Cytotoxicity assays revealed HDAC inhibitors to be more cytotoxic to U-87 MG cells than BET inhibitors, with significant differences between belinostat and the BET inhibitors at the highest concentrations tested (Fig. 2F). Consequently, our drug discovery efforts focused on medicinal chemistry to identify potent, brain-permeable BET inhibitors that enhance sCLU levels with a favorable therapeutic index.

To further investigate the molecular mechanisms underlying effects of our HTS hits on sCLU levels, we also tested other commercially available selective HDAC and BET inhibitors. Inhibition of HDAC1, HDAC3, and HDAC6 increased sCLU levels but was less potent than pan-HDAC inhibition with vorinostat (Supplementary Fig. S1A, 1B). Similarly, bromodomain (BD1/BD2) selective inhibitors such as MS436 or ABV-744 were less potent sCLU enhancers than pan-BET inhibitors such as (+)-JQ1 (Supplementary Fig. S1C, Fig. 2E). This finding is important because more selective compounds, though less potent, may have reduced risk for adverse effects, a primary cause of drug attrition^28^. Combined treatment with HDAC and BET inhibitors did typically significantly increase sCLU levels as compared to single compounds, but the effect was not additive or synergistic and was only observed at micromolar concentrations (Supplementary Fig. S1D). Given their greater potency, subsequent medicinal chemistry efforts to develop brain-permeable sCLU enhancers were based on BET inhibitors.

### Design, synthesis, and evaluation of novel sCLU enhancers

**Fig. 3:**
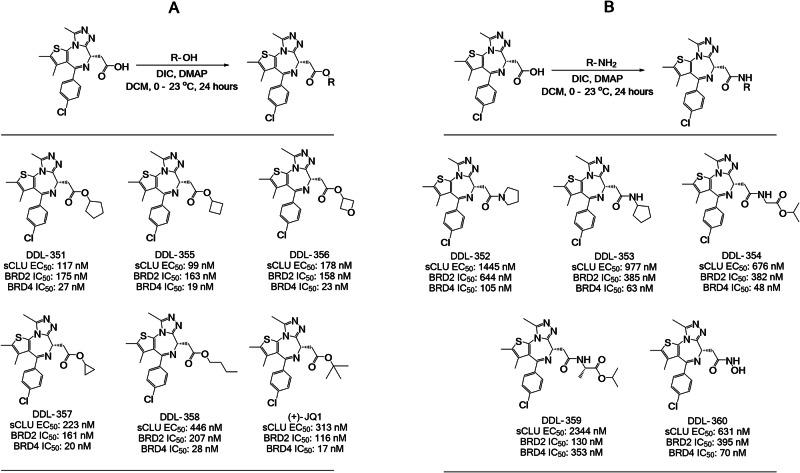
Synthetic scheme and structure activity of novel sCLU enhancers. **A** General synthetic scheme used for the synthesis of ester-containing (+)-JQ1 analogs, and the biological activity (sCLU EC_50_, BRD2/4 IC_50_) for ester-containing analogs, DDL-351, -355, -356, -357, and -358. **B** General synthetic scheme used for the synthesis of amide-containing (+)-JQ1 analogs, and the biological activity (sCLU EC_50_, BRD2/4 IC_50_) for amide-containing analogs DDL DDL-352, -353, -354, -359, and -360. Four analogs had increased potency for sCLU enhancement, relative to (+)-JQ-1.

We used the more potent sCLU enhancer (+)-JQ1 (Fig. 2E), as a starting point to develop novel, potent and brain permeable analogs. Ten novel (+)-JQ1 NCE analogs were designed, synthesized and evaluated in vitro for their potency as sCLU enhancers (Fig. 3) (Synthesis details are presented in Supplementary Materials). Computer-aided molecular docking was initially performed for (+)-JQ1 (Supplementary Fig. S2A and B) in bromodomain 1 of the bromodomain-containing protein 4 (BRD4) crystal structure (PDB: 3MXF) to model potential protein-ligand binding interactions and facilitate design of new chemical entities (NCEs). Subsequently, after lead selection based on in vitro testing, docking was also performed for DDL-357 (Supplementary Fig. S2C-E), which was found to have a Lead Finder (LF) score similar to that of (+)-JQ1 (Supplementary Fig. S2).

Two synthetic approaches were used to generate these analogs of the BET inhibitor (+)-JQ1. Analogs DDL-351, -355, -356, -357, and -358 were synthesized via an ester coupling reaction between (+)-JQ1 carboxylic acid and an alcohol-containing substituent (2 equiv), in the presence of 4-dimethylaminopyridine (DMAP; 2 equiv) and diisopropylcarbodiimide (DIC; 2 equiv) **(**Fig. 3A, C**)**. Analogs DDL-352, -353, -354, and -359 were synthesized via an amide coupling reaction between the (+)-JQ1 carboxylic acid (1 equiv) and an amide-containing substituent (2 equiv), in the presence of 1-ethyl-3-(3-dimethylaminopropyl)carbodiimide (EDC; 2 equiv.), hydroxybenzotriazole (HOBt; 2 equiv.), and N-N, diisopropylethylamine (DIPEA; 2.5 equiv.) (Fig. 3B, D).

Dose-response curves demonstrated that, overall, the ester-containing analogs such as DDL-351 exhibit greater potency for enhancing sCLU in vitro compared to their amide-containing compounds such as DDL-353 (Fig. 3C, D; Supplementary Fig. S3A). Bromodomain-containing protein 2 and 4 (BRD2/4) inhibition assays show a similar trend in potency for these analogs against the molecular drug targets (Supplementary Fig. S3B and C). The structure activity relationship (SAR) analysis shows a general correlation of sCLU EC_50_ to the BRD4 IC_50_ but less so to BRD2 IC_50_ (Supplementary Fig. S3B and C)_._ DDL-355 exhibited the greatest potency in-vitro with BRD2/4 IC_50_s of 163 and 19 nM and a sCLU EC_50_ of 99 nM followed by DDL-356 and 357. The NCE DDL-357 has similar affinity for BD1 and BD2 bromodomains like (+) JQ1 (Supplementary Fig. S3D).

To identify compounds suitable for treating CNS disorders in vivo, various in vitro assays were used to evaluate structural modifications influencing drug ADME, including solubility, liver microsome stability, plasma binding, brain tissue binding, and BBB permeability (Table 1). Good in vitro activity alone does not guarantee favorable in vivo activity without adequate bioavailability and half-life^29,30^. Due to high development costs and candidate attrition rates, drug discovery strategies now emphasize simultaneous evaluation of drug physicochemical and ADME properties alongside efficacy^30^. This is especially important for CNS disorders, as the BBB selectively excludes nearly 100% of large-molecule neurotherapeutics and over 98% of small-molecule drugs^31^. Desired in vitro ADME property values and criteria for continued in vivo evaluation were: Kinetic Solubility > 50 µM; Liver Microsomal Stability t1/2 > 1 hour; Plasma Binding < 90%; Brain Tissue Binding < 80%; BBB Permeability Pm > 1.0 = CNS + ; sCLU EC50 < 313 nM. Of the ten compounds assessed, only the lead candidate DDL-357 met these criteria.

**Table 1:** Physicochemical Properties of sCLU Enhancer Analogs

Compound	MW (Da)	Kinetic Solubility [μM]	Microsomal Stability [t1/2(min)]	Plasma Binding [% bound]	Brain Tissue Binding [% bound]	PAMPA [Pm]	EC50 for sCLU increase [nM]
**(** + **)-JQ1**	456.99	86.5	27	85	87	1.44	313
**DDL-351**	469.00	57.5	3	99	94	1.35	117
**DDL-352**	453.99	>100	62	96	64	0.98	1445
**DDL-353**	468.02	>100	8	93	79	1.08	977
**DDL-354**	500.01	91.1	12	91	67	0.76	676
**DDL-355**	454.97	63.7	27	86	88	1.42	99
**DDL-356**	456.95	>100	75	79	57	0.96	178
**DDL-357**	440.95	80.0	61	88	75	1.66	223
**DDL-358**	456.99	59.3	14	98	93	1.42	446
**DDL-359**	514.04	>100	13	94	44	0.71	2344
**DDL-360**	415.90	>100	>120	88	59	1.09	631

Desired physicochemical properties: solubility >50 μM; microsomal stability t_1/2_ > 1 hr; plasma binding <90%; brain tissue binding <80%; PAMPA Pm>1.0; sCLU enhancement EC50 < 313 nM. Values outside desired range shown in red.

Given the key role of drug toxicity in attrition during clinical development^28^, the CNS gliotoxicity (U-87 MG), neurotoxicity (SH-SY5Y), nephrotoxicity (HEK 293 T), and hepatotoxicity (HEPG2)^32,33^ of DDL-357 were assessed. DDL-357 did not show overt toxicity at concentrations as high as 50 µM (Ctox ≥ 50 µM) (Supplementary Fig. S4A). DDL-357 was also evaluated in a human ether-a-go-go-related gene (hERG) potassium channel inhibition assay and showed minimal effect with an EC50 > 100 µM (Supplementary Fig. S4B).

### Pharmacokinetics of DDL-357 in vivo in mice

**Fig. 4:**
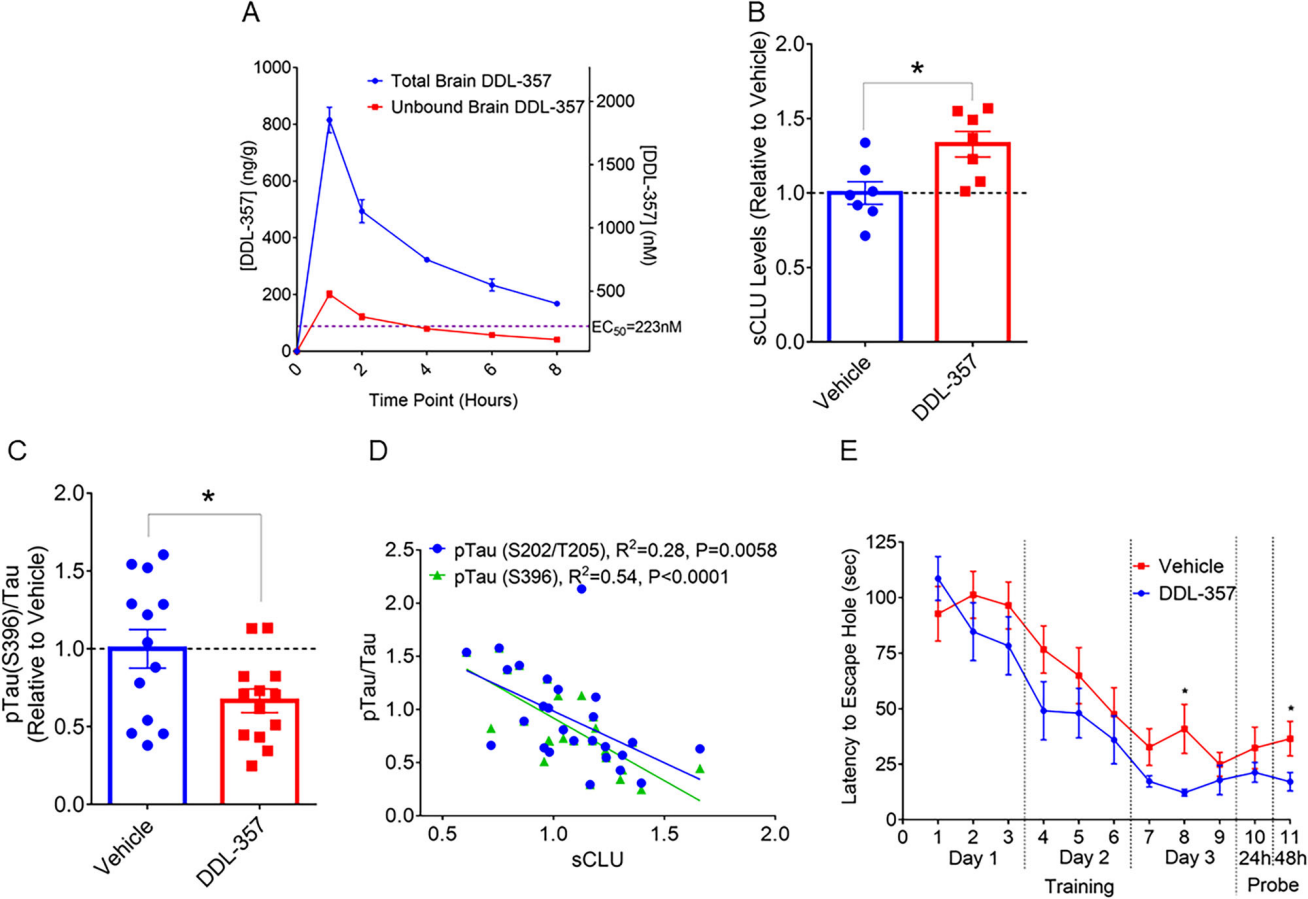
Oral administration of DDL-357 increases sCLU levels and improves memory in AD mouse models. **A** DDL-357 brain concentrations (total in blue and unbound in red), 1, 2, 4, 6 and 8 hours following oral administration at a dose of 30 mg/kg (*n* = 3 per time point). **B** sCLU levels in the hippocampus of ApoE4TR-5XFAD mice following subchronic, 2-week oral administration of DDL-357 at 15 mg/kg BID (*n* = 7 per group, DDL-357 - 4 females and 3 males; vehicle - 3 females and 4 males). For chronic 6-week testing of oral 15 mg/kg BID in the 3xTg-AD model: **C** phosphorylated tau (ptau)/total tau levels at S396 in the hippocampus (*n* = 13 per group, DDL-357 - 4 females and 9 males; vehicle - 5 females and 8 males), **D** scatter plot and Pearson correlation coefficients for the ptau/total tau ratio for S396 and S202/T205 vs. sCLU levels in the hippocampus, and **E** latency to escape hole (sec) in both training a probe trials in the Barnes Maze. All results graphed as mean ± SEM. The statistics for were performed using unpaired two-tailed t-test, where *p ≤ 0.05; **p < 0.01; ***p < 0.001; ****p < 0.0001). Pearson correlation coefficients.

Pharmacokinetic (PK) analyzes were conducted to determine if DDL-357 could reach therapeutically-relevant brain concentrations. In mice, DDL-357 showed good brain bioavailability, reaching a maximum concentration of 1850 nM one hour after oral administration at 30 mg/kg (Fig. 4A). Considering only total brain drug concentration may be misleading, as only unbound drug exerts pharmacological effects according to the free drug hypothesis^34^. Integrating in vitro brain tissue binding studies with in vivo pharmacokinetics is therefore essential. When DDL-357’s brain tissue binding (75.3%) is considered, the unbound brain concentration is still 457 nM, nearly twice its sCLU EC_50_ or minimum target concentration (EC_50_ = 223 nM) (Fig. 4A). Further analyzes confirmed that unbound brain concentrations of DDL-357 one hour post administration at doses of either 3 or 10 mg/kg were below the sCLU EC_50_ (Supplementary Fig. S5). Based on these results, twice daily (BID) treatments of 15 mg/kg were selected for efficacy testing of DDL-357 in two AD mouse models (ApoE4TR-5XFAD and 3xTg-AD).

Pharmacokinetics experiments also indicate that an additional sCLU enhancing candidate, DDL-356, may be a promising candidate for future in-vivo study, as it reaches higher concentrations of unbound drug in the brain above its sCLU EC_50_ at lower doses (Supplementary Fig. S6A and B).

### Subchronic dosing with DDL-357 increases sCLU in ApoE4TR-5XFAD mouse brain

The initial subchronic, two-week in vivo testing was performed to assess sCLU levels in brain in an ApoE4-expressing mouse model of AD, ApoE4-TR:5xFAD. Oral administration of DDL-357 (15 mg/kg BID or 30 mg/kg daily) in this model resulted in a significant increase in sCLU levels in the hippocampus as compared to vehicle-only treated mice (Fig. 4B). While the mean for Aβ1-42 was lower in DDL-357-treated mice as compared to controls, the difference did not reach significance (Supplementary Fig. S7A).

### Chronic dosing with DDL-357 lowered the p-tau/tau ratio in brain and improved learning and memory in 3xTg-AD mice

For the subsequent, chronic 6-week oral testing at 15 mg/kg BID (30 mg/kg daily), the 3xTg-AD model was used that expresses human tau with the MAPT P301L mutation in addition to mutations in PS1 and APP, to enable analysis of DDL-357 effects on tau phosphorylation. While mean sCLU levels were higher, and mean Aβ1-42 levels lower, with DDL-357 treatment of this model, the differences were not significant (Supplementary Fig. S7B and C, respectively). Similarly, reduction of tau phosphorylation at Ser202/Thr205 was lower, but not significantly so (Supplementary Fig. S7D), but the reduction of tau phosphorylation at Ser396 was significant (Fig. 4C). Further, an inverse correlation between sCLU levels and the p-tau/tau ratio in brain was observed (Fig. 4D). Overall, the p-tau/total tau ratio was greater for males than females, with more variability in the males (Supplementary Fig. S7E and F)^35,36^.

In the Barnes maze assessment of learning and memory, after the first training session on Day 1 of training, 3xTg-AD mice treated with DDL-357 performed better overall during both the training and probe phases, with significant differences being observed on Day 3 of training and in the 48-hour probe (Fig. 4E).

### DDL-357 increased proteins related to mitochondrial function, synaptic plasticity and protein aggregate clearance in ApoE4TR-5XFAD mice

**Fig. 5:**
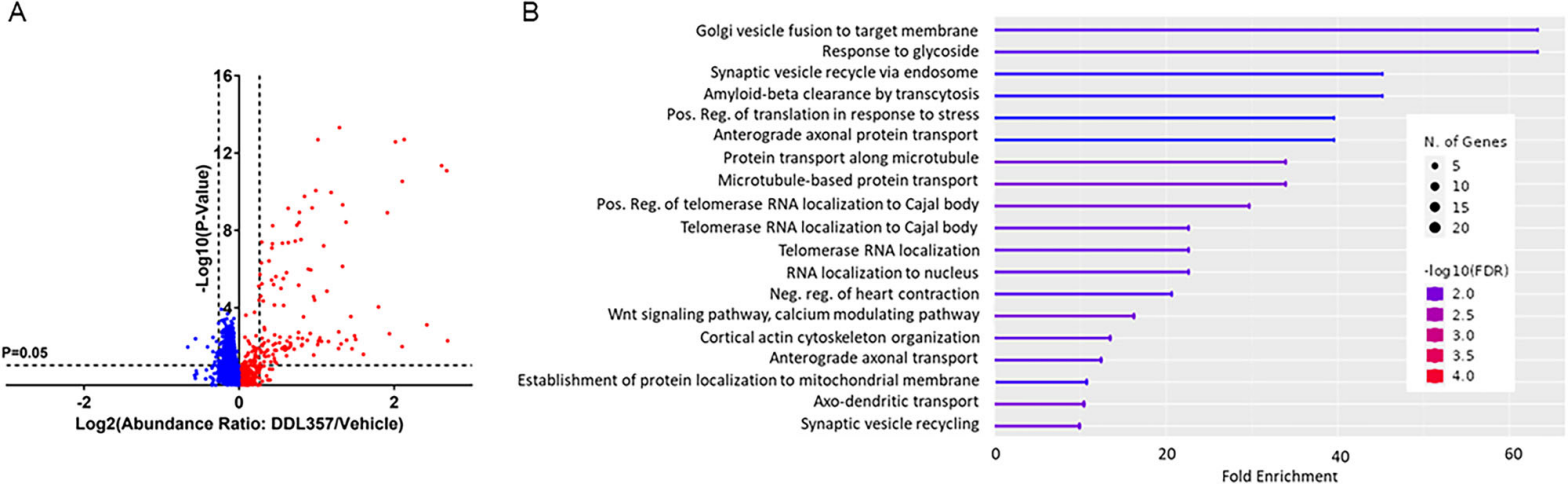
Proteomics of DDL-357-treated murine brain. **A** Volcano plot abundance ratio of proteins in hippocampi of DDL-357/Vehicle-treated ApoE4TR-5XFAD mice (DDL-357 oral dose of 15 mg/kg BID). The−log_10_ (*p*-value) is plotted against log_10_ abundance ratio. Proteins upregulated by DDL-357 in red, those reduced in blue. **B** Results of gene set pathway enrichment analysis performed on genes significantly upregulated by DDL-357 in-vivo.

To further characterize the pharmacological effects of DDL-357 in-vivo, an unbiased proteomics analysis was performed to identify differentially expressed proteins in the hippocampus of the vehicle versus DDL-357-treated ApoE4TR-5XFAD mice. The abundance ratios of proteins affected by DDL-357 treatment is shown in Fig. 5A, with proteins increased by DDL-357 treatment in red. A subsequent gene-set enrichment analysis determined many of the proteins significantly upregulated at least 1.3-fold by DDL-357 treatment to be associated with biologically relevant processes, including pathways of neurodegeneration (Fig. 5B).

Proteomic analysis identified upregulated proteins associated with mitochondrial function and known to be disrupted during AD pathogenesis (Table 2)^37,38^. This included several proteins from complex I, NADH:ubiquinone oxidoreductase (NDUFA13, NDUFC2, NDUFS5, NDUFB4), and complex III, ubiquinol–cytochrome c oxidoreductase (UQCRFS1), of the mitochondrial respiratory chain. These proteins are decreased in AD patient brains, and this decrease has been suggested to be causative of the disease^37^. Increasing these proteins has important implications for maintaining mitochondrial bioenergetics vital to supporting neuronal function and preventing oxidative damage in AD (Supplementary Table 1)^38–40^. Their upregulation suggests a potential role in mitigating mitochondrial dysfunction, increasingly recognized as a driver of cognitive decline in AD^38^.

**Table 2:** Functional Role of Proteins Upregulated by DDL-357 in ApoE4TR-5XFAD Mouse Brain Implicated in AD Pathology

Mitochondrial Function	Protein/Peptide Aggregate Clearance	Synaptic Plasticity
Gene Name		
BLMH	PSMD3	ALCAM
NDUFC2	TRPV2	PPP3R1
API5	RAB8A	DLGAP2
NDUFS5	SMCR8	NPTXR
PRDX3	GRPEL1	GNA!2
SLC4A10	Rab27b	HTT
NDUFB4	CCT2	SNAP29
UQCRFS1	EIF5A	ITSN1
NDUFA13	VBP1	PLCB1
	UBE2L3	

Proteomics in ApoE4TR-5XFAD mouse brain also revealed increased expression of various proteins important for synaptic plasticity, and protein aggregate clearance, which are also implicated in AD pathogenesis (Table 2)^37,38,41–43^. The specific biological processes include Aβ clearance by transcytosis, WNT signaling pathway, synaptic vesicle recycling, and protein localization to mitochondrion^37,41,42^. The upregulated proteins included SMCR8, TRPV2, RAB8A, RAB27B, and GRPEL1, known to enhance autophagic-lysosomal clearance of protein aggregates, including Aβ and alpha-synuclein (αSyn)^38^. This was accompanied by increases in molecular chaperones (CCT2, CCT3, VBP1), as well as additional components of the endo-lysosome autophagy pathway (VSP39) and the ubiquitin-proteasome system degradation pathway (UBE2L3, PSMD3)^39^. Several proteins critical for maintaining synaptic function, dendritic spine morphology, long-term potentiation, and associated learning and memory processes were also upregulated (SNAP29, ALCAM, ITSN1, DLGAP2, NPTXR) (Table 2; Supplementary Table S1).

### DDL-357 increased proteins related to synaptic function in 3xTg-AD mice

Brain tissue from 3xTg-AD mice in the chronic study of DDL-357 also underwent proteomic analysis. Proteins found to be increased included those important for maintaining synaptic function, dendritic spine morphology, long-term potentiation, and associated learning and memory processes (NPTN, KCNJ6, ITM2B, DUSP3, PLCB3, LRRC4B, MBNL1, DLGAP2, NCS1, RPSA, ALCAM, BRK1, GUCY1B1, CKB, OSCP1) (Supplementary Fig. S8 and Table S2). One such protein upregulated by DDL-357 is a positive allosteric modulator of GUCY1B1 that boosts cGMP production, enhancing neuronal function, cerebral blood flow, reducing neuroinflammation, and improving cellular bioenergetics in preclinical models; it is currently in clinical trials^40^.

### DDL-357 increased mitochondrial oxygen consumption, and HN and SirT1 expression in U-87 and SH-SY5Y cells, and neurite outgrowth in iPSC-derived neurons

**Fig. 6:**
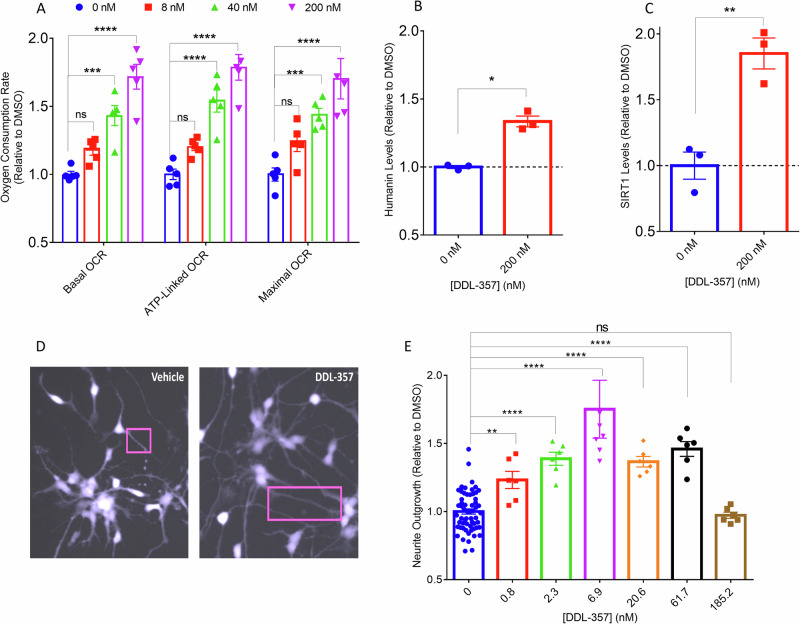
DDL-357 enhances mitochondrial function and neurite outgrowth in human neuronal and iPSCs **A** Basal, ATP-linked, and Maximal oxygen consumption rate in SH-SY5Y neuroblastoma cells, following treatment with DDL-357 for 24 h at concentrations of 0, 8, 40 and 200 nM (*n* = 5). **B** Humanin levels in SH-SY5Y cells, following 24 h treatment with DDL-357 at concentrations of 200 nM (*n* = 3). **C** Sirtuin 1 levels in SH-SY5Y cells, following 24 h treatment with DDL-357 at concentrations of 200 nM (*n* = 3). **D** Confocal image of Calcein-AM stained iPSC-derived neurons 48 h after treatment with 0.5% DMSO and DDL-357 at a concentration of 6.9 nM. **E** Measurements of neurite outgrowth in iPSC-derived neurons following treatment with 0.5% DMSO or DDL-357 for 48 h at concentrations 0, 0.8, 2.3, 6.9, 20.6, 61.7, 185.2 nM (*n* = 60 for 0 nM while *n* = 6 for the other concentrations). All results graphed as mean ± SEM. All statistics were performed as one-way ANOVA or in panel **B** and **C** as unpaired two-tailed t-test (*p ≤ 0.05, **p < .01, ***p < 0.001; ****p < 0.0001).

To reveal the possible functional impact of alteration of proteins involved in mitochondrial function, oxygen consumption was assessed in untreated and DDL-357-treated SH-SY5Y neuronal cells. DDL-357 increased the oxygen consumption rate, an established measure of mitochondrial respiration^44^, in a dose-dependent manner (Fig. 6A). A similar increase in oxygen consumption was also observed in U-87 cells (Supplementary Fig. S9D).

In addition to increasing sCLU (Supplementary Fig. S9A), treatment of SH-SY5Y cells with DDL-357 at a concentration of 200 nM significantly increased expression of the master metabolic regulator sirtuin 1 (SirT1) and mitochondrial-derived peptide humanin (HN) (Fig. 6B, C, respectively; Supplementary Fig. S9B, and C), both of which have numerous neuroprotective functions shown to lessen AD pathology and improve cognition^45–47^. The concentrations at which these effects were observed are physiologically relevant to brain levels of DDL-357 achieved in vivo.

DDL-357 was also evaluated for its ability to promote neurite outgrowth in human iPSC-derived neurons. Concentrations of 0.8–61.7 nM DDL-357 were associated with significantly increased neurite outgrowth (Fig. 6D, E). Of note, this effect occurred at concentrations achieved by the free unbound drug in the brain (Supplementary Fig. S5).

## Discussion

Our discovery of a novel class of brain permeable BET inhibitors as sCLU enhancers that improve memory presents an opportunity for development of a new therapeutic for AD. The cognitive improvement observed using a BET inhibitor in the clinic provides further support^27^. Despite the limitations of mouse models for assessment of potential therapeutic benefit in a complex human disease like AD^48^, our preclinical testing of lead candidate DDL-357 demonstrates its ability to enhance sCLU levels in brain of the ApoE4TR-5XFAD model after subchronic oral treatment. In chronic testing decrease of the p-tau/total tau ratio, and improvement of memory was seen in the 3xTg-AD model. These in vivo studies provide initial proof of efficacy for DDL-357.

The findings from proteomics analysis of DDL-357-treated murine brain tissue reveal pathways affected by DDL-357 and that may be affected by BET inhibition generally. Enhancement of key neurotrophic proteins associated with mitochondrial function, synaptic plasticity, and disease relevant protein aggregate clearance was observed. The opportunity for improvement of mitochondrial function is appealing in AD, as numerous mitochondria-targeting interventions are currently being investigated in clinical trials to prevent AD progression^49^.

One of the enhanced proteins, ITSN1, is a key component of the Reelin signaling pathway and thus may have the potential to act in conjunction with sCLU to reduce levels of p-tau and thus neurofibrillary tangle formation. Given that these critical regulatory mechanisms are known to become progressively impaired with aging, identifying interventions that regulate proteostasis network machinery may be instrumental in effectively controlling the accumulation and clearance of disease-relevant aggregation-prone proteins such as Aβ in AD^43^.

Given the limited therapeutic benefits of current treatments focused on single protein targets associated with AD pathology - such as Aβ-directed antibodies that effectively clear the brain of plaques, but do not address the neuronal functions and networks that are damaged in AD - new approaches are needed.

Limitations of the present studies are largely attributed to the mice n numbers used for in vivo testing, and the need for better understanding of the influence of sex dimorphism in 3xTg mice on responses to DDL-357. In future studies, which may include testing of additional analogs such as DDL-356, we plan to increase cohort numbers to power statistics and test equal numbers of animals of each sex along with longer treatment period. In advanced pre-clinical testing, additional species such as a rat model of AD, would also be used.

Continued pre-clinical development is warranted for lead candidate DDL-357, which represents a possible new class of therapeutics that might increase the expression of neuroprotective protein networks and could work in tandem with the current AD therapeutics to improve cognition. While further analysis of AD-related biomarkers, off-target effects in the brain, and IND-enabling studies are needed, our drug discovery effort underscores their potential as valuable therapeutic avenue for AD. The findings described here suggest this class of therapeutic, if successfully developed, may also be efficacious in the treatment of other neurodegenerative proteinopathies, such as Parkinson’s disease and amyotrophic lateral sclerosis.

## Methods

### Human sCLU AlphaLISA

Cell culture supernatant (2 μL) was added to a 384-well proxy plate preloaded with AlphaLISA HiBlock buffer (2 µL, 1X, CAT#AL004C, Perkin Elmer). Then an anti-sCLU antibody mixture (2 µL, mAb J84, biotinylated (10 nM, Cat# 3717-6-1000, Mabtech) and mAb CLU aa 22-227 (Cat#AF7084, R&D Systems), Acceptor bead conjugated (50 µg/mL, Cat#6772001, Perkin Elmer)) was added and incubated at room temperature for 1 hour. AlphaScreen Streptavidin Donor beads (2 µL, Cat#6760002S, Perkin Elmer) were added, incubated for an additional 30 min in the dark, and the plate was read in an Envision plate reader.

### Mouse sCLU ELISA

Brain tissue was homogenized and diluted to a protein concentration of 50 ng/mL. The Mouse Clusterin ELISA Kit (Cat#ab199079, Abcam) was performed according to the manufacturer’s protocol.

### High throughput screening

Human glioblastoma U-87 MG cells were proliferated in EMEM with 10% FBS and 1% P/S (complete medium) in 10 cm dishes until approximately 90% confluent. Following trypsinization, a suspension of 2 × 10^5^ cells/mL (25 µL) were added to 384-well plates containing complete medium (25 µL) and library compounds from 9 plates (5 µM). After incubation for 30 h at 37 °C and 5% CO_2_, 2 μL of cell culture supernatant were analyzed via human sCLU AlphaLISA as described above. Cell culture medium and cells were added to the 384-well plates using the MultiDrop. The BioMek FX was used to pin the drugs and transfer the cell culture supernatant. The MANTIS liquid handler was used to added human sCLU AlphaLISA reagents.

### Dose response analysis

Human glioblastoma U-87 MG cells were proliferated in EMEM with 10% FBS and 1% P/S (complete medium) in 10 cm dishes until approximately 90% confluent. Following trypsinization, a suspension of 2 × 10^5^ cells/mL (25 µL) were added to 384-well plates containing complete medium (25 µL) with various concentrations of compounds (5, 14, 41, 123, 370, 1111, 3333, 10000 nM). After incubation for 30 hours at 37 °C and 5% CO_2_, 2 μL of cell culture supernatant were analyzed via human sCLU AlphaLISA as described above.

### Cell viability and toxicity analysis

Cell viability and toxicity were simultaneously assessed using the MultiTox-Fluor Multiplex Cytotoxicity Assay (Cat# PRG9200, Fisher Scientific) according to the manufacturer’s protocol.

### Molecular docking and scoring of protein–ligand binding energy

Two compounds, ( + )JQ-1 and DDL-357 underwent molecular docking analysis in BD1 domain of BRD4. Molecular docking and scoring was performed using Flare software (Cresset, version 7) and calculations were run under MacOSX v14 on computer with Apple Silicon M2 Ultra CPU with 24 cores and GPU with 60 cores. The Lead Finder (LF) scores were obtained for (+)-JQ1 and DDL-357. Calculations used OpenFF force field (V2.2.0) with charge method AM1-BCC. Simulation analysis was done for the lead candidate DDL-357 using Flare software (Cresset, version 7). The Simulation length - 100 ns, solvent model was TIP3P with explicit water. Solvent box buffer: 10.0 Å. Solvent box shape: Truncated octahedron. Solvent ionic strength: Just neutralize, simulation settings: 298 °K at 1 bar pressure. Simulation was carried out after initial equilibration for 200 ps, with time step of 4.00 fs. Hydrogen mass repartitioning coefficient was 1.50.

The Lead Finder docking algorithm^50^. Lead Finder combines a generic algorithm search with local optimization procedures. Three different scoring functions are employed and optimized for the accurate prediction of 3D docked ligand poses, protein-ligand binding energy and rank-ordering of active and inactive compounds in virtual screening experiments. Pocket Detection in Flare is based on the fpocket methods^51^, and can be used to identify and characterize pockets and cavities within a protein structure, enabling the identification of possible drug binding active sites, water binding pockets, channels and small cavities, large solvent-exposed sites. The docking procedure used for preparation of the molecular files for docking is an integral part of Flare software. The scoring functions used for prediction of docked ligand poses and their protonation states are internal routines within Flare software.

### Aβ and p-tau/total tau AlphaLISA analysis

Hippocampal tissue was sonicated in TPER buffer containing 1x protease and phosphatase inhibitors (Fisher catalog # 78429) on ice. Samples were centrifuged at 16000xg for 10 min at 4 degrees C. A BCA protein quantification assay was performed on each sample in duplicate. Aliquots of 40 uL were stored in the -80 freezer.

A bicinchoninic acid (BCA) assay was implemented to accurately standardize each treatment groups’ protein density. Nine BSA standards ranging in concentration from 0 ug/ml to 2000 ug/ml were prepared in the same media as the samples. 2 uL of the samples were added to 8 uL of PBS and 10 uL of each standard were plated into a 96 well plate in triplicates. The working reagent was made by combining Reagent A and Reagent B from the Pierce BCA kit (CN:23225) in a 50:1 ratio and 100 uL was added to each well. The plate was then incubated for 30 min at 37 °C in the dark. The optical densities of the cell lysates were measured using a spectrophotometer set at 562 nm and used to calculate the final concentrations through comparing the optical density (OD) values for each treatment group with the OD values of nine standards.

A sample of brain homogenate (8.8 mg/mL) was tested at 10 dilutions using the Tau AlphaLISA (Revvity Cat#AL271C) (32, 64, 128, 256, 512, 1024, 2048, 4096, 8192, 16384-fold) and, separately, the anti-tau phosphor AT8 and S396 (Fisher catalogs MN1020B and 44-752 G, respectively) antibodies (2, 4, 8, 16, 32, 64, 128, 256, 512, 1024-fold). AlphaLISA assays were by loading 2 µL of the anti-tau acceptor bead mixed with anti-tau or anti-ptau biotinylate antibodies in the 384-well plate, then 2 uL sample was loaded and incubated for 1 h at room temperature. AlphaScreen Streptavidin Donor beads (2 µL, Cat#6760002S, Perkin Elmer) were added, incubated for an additional 30 minutes in the dark, and the plate was read in an Envision plate reader.

Additional assays: The Aβ1-42 ELISA assay was performed on hippocampus brain homogenate according to the manufacturer’s protocol (Fisher KHB3441). Humanin in cell culture lysate was quantify by ELISA assay, according to the manufacturer’s protocol (MyBioSourc Cat# MBS7607247). SirT1 in cell culture lysate was quantify using AlphaLISA. Cell lysate (2 μL) was added to a 384-well proxy plate preloaded with 2 µL anti-SiRT1 antibody mixture (ABCAM catalog AB110304, biotinylated (10 nM) and ABCAM catalog AB104833, acceptor bead conjugated (50 µg/mL, Cat#6772001, Perkin Elmer)) was added and incubated at room temperature for 1 hour. AlphaScreen Streptavidin Donor beads (2 µL, Cat#6760002S, Perkin Elmer) were added, incubated for an additional 30 min in the dark, and the plate was read in an Envision plate reader.

### Compound synthesis via amide coupling

(S)-2-(4-(4-chlorophenyl)-2,3,9-trimethyl-6H-thieno[3,2-f][1,2,4]triazolo[4,3-a][1,4]diazepin-6-yl)acetic acid (1 equiv), amine-containing reagent (2 equiv), 1-Ethyl-3-(3-dimethylaminopropyl)carbodiimide (EDC; 2 equiv.), Hydroxybenzotriazole (HOBt; 2 equiv.), and N-N, Diisopropylethylamine (DIPEA; 2.5 equiv.) were added to a round bottom flask. The reagents were solubilized using methylene chloride and the reaction mixture was stirred at room temperature for 16 h. The crude product was concentrated in vacuo and then purified via flash column chromatography. A mobile phase of hexanes:ethyl acetate (time/% ethyl acetate: 0/0, 2/0, 7/50, 15/100, 20/0) was first utilized to elute any existing impurities, and the product was eluted in the DCM:MeOH mobile phase (time/%MeOH: 0/0, 4/0, 35/15, 42/20, 45/50, 50/0). Identity and purity of each compound was confirmed via LC-MS, LC-UV/Vis, and ^1^H NMR. The synthesis and analytical data for each compound is provided in Supplementary Materials.

### Compound synthesis via ester coupling

(S)-2-(4-(4-chlorophenyl)-2,3,9-trimethyl-6H-thieno[3,2-f][1,2,4]triazolo[4,3-a][1,4]diazepin-6-yl)acetic acid (1 equiv), alcohol-containing reagent (3 equiv), and 4-Dimethylaminopyridine (DMAP; 2 equiv) were added to a round bottom flask. The reagents were solubilized using methylene chloride (800 µL) and was brought to 0 °C in an ice bath. A solution of diisopropylcarbodiimide (DIC; 2 equiv) in methylene chloride (200 µL) was added dropwise over the course of 30 min and then slowly brought to room temperature. After stirring for 24 h, the reaction mixture was filtered through a filter funnel and concentrated in vacuo. The crude product was purified via flash column chromatography. A mobile phase of hexanes:ethyl acetate (time/% ethyl acetate: 0/0, 2/0, 7/50, 15/100, 20/0) was first utilized to elute any existing impurities, and the product was eluted in the DCM:MeOH mobile phase (time/%MeOH: 0/0, 4/0, 35/15, 42/20, 45/50, 50/0). Identity and purity of each compound was confirmed via LC-MS, LC-UV/Vis, and ^1^H NMR. The synthesis and analytical details for each compound is provided in Supplementary Materials.

### Purity analysis via proton nuclear magnetic resonance spectroscopy (^1^H NMR)

Analysis of purity by magnetic resonance spectroscopy was done at the UCLA Molecular Instrumentation Center (MIC; Ignacio Martini, Ph.D., Director). ~4 mg of compound were diluted in 750 µL of MeOH-D4 and analyzed using a AV400 NMR spectrometer (Bruker) containing a 5 mm broadband Z-gradient probe with Automatic Tune and Match (ATM). The analysis method consisted of a 64-scan proton NMR (¹H-NMR) utilizing default parameters. Predicted ¹H-NMR spectra were obtained using the ChemNMR ¹H estimation function in ChemDraw (PerkinElmer). The experimental data was visualized and interpreted using Mnova (Mestrelab Research).

### Liver microsome stability assay

An aliquot (1 µL) of test compound (1 mM, 100% DMSO) was added to an aqueous liver microsome solution (1000 µL, PBS pH 7.4, 0.5 mg/mL human liver microsomes (Thermo Fisher Scientific, Cat#HMMPL), 2 mM NADPH, 2 mM MgCl2) and incubated at 37 °C for 120 min. Aliquots (50 µL) of the microsome solution were taken at various time points (0, 5, 10, 15, 30, 60, 90, 120 min) and added to a reaction quenching solution (200 µL 100% Acetonitrile) containing an internal standard. Solutions were clarified by centrifugation (16,000 x g, 5 min), and the supernatants were transferred to new tubes and lyophilized. Samples were reconstituted in 100 µL of 50/50/0.1 (Water/Acetonitrile/Formic Acid) prior to analysis via liquid chromatography-tandem mass spectrometry (LC-MS/MS). Chromatographic peak areas normalized to the internal standard were plotted at each time point and the half-life (t_1/2_) of compound in liver microsomes was determined by using the trendline equation to calculate the time at which compound abundance was 50% of that at time point 0 (t_0_).

### Kinetic solubility

Test compound (10 mM, 100% DMSO) was diluted separately into aqueous buffer (100 µM; PBS pH 7.4) and DMSO at various concentrations (1000, 100, 10, 1, 0.1 µM). The solutions were then incubated at 37 °C for 90 min and centrifuged (16000xg, 5 min). An aliquot of each supernatant was analyzed by UV/Vis (if possible) or LC-MS/MS. A standard curve was made by plotting the known amount of analyte per standard in DMSO vs. absorbance or chromatographic peak area. Kinetic solubility (mM) was calculated using the trendline equation with maximum absorbance or chromatographic peak area observed in the aqueous sample.

### Plasma and brain tissue binding assays

Brain tissue was homogenized in PBS (pH 7.4) (1: 3 weight(mg)/volume(µL)) and the protein concentration was determined using the Micro BCA™ Protein Assay Kit (Thermo Fisher Scientific, Cat#23235). Brain homogenate was clarified and diluted to 20 mg/mL in PBS (pH 7.4). Either brain homogenate or plasma was and added to Slide-A-Lyzer™ MINI Dialysis Devices, 10 K MWCO dialysis cups (Thermo Fisher Scientific, Cat#PI88401) in a 48-well plate containing PBS (500 µL; pH 7.4). 1 µL of 1 mM compound was added to the brain homogenate (Final Concentration: 2 µM compound, 0.5% DMSO) and incubated on a rocker for 4.5 hours at 37 °C. 50 µL of brain homogenate or plasma (within the dialysis cup) and PBS (within the 48-well plate) were transferred to new microcentrifuge tubes containing 400 µL of quenching reagent (100% Acetonitrile) containing internal standard. Solutions were clarified by centrifugation (16,000 x g, 5 min), and the supernatants were transferred to new tubes and lyophilized. Samples were reconstituted in 100 µL of 50/50/0.1 (Water/Acetonitrile/Formic Acid) prior to analysis via liquid chromatography-tandem mass spectrometry (LC-MS/MS). The % of the unbound drug (f_u,bound_) was calculated using the following equation:

% Bound = [1− (PBS chromatographic peak area/ brain homogenate or plasma chromatographic peak area)] x 100

### Blood–Brain Barrier (BBB) permeability assay

A liquid chromatography-ultraviolet/visible spectroscopy (LC-UV/Vis) assay was performed on a 1290 Infinity HPLC system (Agilent Technologies) with an HPLC column containing immobilized phosphatidylcholine (IAM.PC.DD, Regis Technologies, Cat#774011, 5 µm 300 Å 100 ×4.6 mm). The HPLC method was a mixture of 6.7 mM phosphate buffer saline (pH 7.4; solvent A) and acetonitrile (solvent B), and a gradient was used for the elution of the compounds (min/%B: 0/20, 20/60, 21/20, 30/20). The retention time of the compound (t_r_) and void volume time of the column (t_0_) were recorded. Blood-brain barrier (BBB) permeability (P_m_) was calculated using the following equations as described by Yoon et al. (10.1177/1087057105281656):

K_IAM_ = (t_r_ − t_0_) / t_0_; *P*_m_ = (K_IAM_ / MW^4^) x 10^10^

Compounds with a *P*_m_ > 1.0 were determined to be BBB permeable (CNS + ) at pH 7.

### In vivo models

All procedures were performed in accordance with protocols approved by the UCLA Institutional Animal Care and Use Committee (IACUC) and guidelines of the NIH. Mouse strains used were C57BL/6 J (Black 6, Jackson Laboratory; JAX stock #000564). All non-AD mice were 4 to 5 mo old at the time of the experiments. The AD model mice consisted of ApoETR-5XFAD mice which express APOE4 under the control of the endogenous mouse APOE promoter were bred to 5XFAD mice (Tg6799) which co-express five FAD mutations (APP K670N/ M671L + I716V + V717I and PS1 M146L + L286V) under the control of the neuron-specific mouse Thy-1 promoter, and backcrossed three times to ApoE-TR mice, resulting in mice homozygous for APOE4, and hemizygous for the 5XFAD transgenes, on a background strain 97% C57Bl/6 J and 3% SJL; mice were then inbred between 5XFAD+ and 5XFAD- resulting in littermates E4 + / + :FAD+ and E4 + / + :FAD-. The AD model mice were 4 to 5 mo old for the in vivo studies. The 3xTg-AD mice are commercially available at The Jackson Laboratory catalog # 034830-JAX, the background strain is B6129SF2/J (The Jackson Laboratory catalog # 101043 and were 8–9 mo old for the in vivo studies. For all the in vivo testing both male and female mice were used.

### In-vivo pharmacokinetics

Following oral administration of compound, mice brain tissue and plasma were collected after euthanasia and perfusion at 1, 2, 4, 6 and 8 h. All animals will be euthanized by ketamine/xylazine injection to allow cardiac puncture and saline perfusion, which is consistent with recommendations of the AVMA Panel on Euthanasia. Brain tissue were homogenized in a bead beater using 5 volumes of ice-cold 80% acetonitrile (1/5; mg of brain/µL of 80% ACN). Plasma analytes were extracted using 4 volumes of ice-cold acetonitrile (1/4; µL of plasma/µL of ACN). Solutions were clarified by centrifugation (16,000 x g, 5 min) and the supernatants were transferred to new tubes and lyophilized. Samples were reconstituted in 100 µL of 50/50/0.1 (Water/Acetonitrile/Formic Acid) prior to analysis via liquid chromatography-tandem mass spectrometry (LC-MS/MS). An internal standard (IS) was added to every sample to account for compound loss during sample processing. Standards were made in drug naïve plasma and brain lysates with increasing amounts of analyte (S1,S2: 0 pmol/ S3,S4: 1 pmol/ S5,S6: 10 pmol/ S7,S8: 100 pmol, S9,S10: 1000 pmol). The standard curve was made by plotting the known amount of analyte per standard vs. the ratio of measured chromatographic peak areas corresponding to the analyte over that of the IS (analyte/IS). The trendline equation was then used to calculate the absolute concentrations of each compound in plasma and brain tissue.

### In-vivo testing of DDL-357

Male and female 3-month-old mice were administered DDL-357 via pipette feeding at a dose of 15 mg/kg BID (30 mg/kg/day). Mice were weighed before the first dose for the calculation of volume to be administered. The formulation used comprised DDL-357 as a 15 mg/mL solution dissolved in a strawberry flavored solution (propylene glycol/water/strawberry syrup, 2/1/1, v/v/v). ApoE4TR-5XFAD mice (7 mice, 3 females & 4 males-vehicle; 7 mice, 4females and 3 males – DDL357) were dosed for two weeks. 3xTg-AD mice (13 mice, 5 females & 8 males-vehicle; 13 mice, 4 females and 9 males- DDL357) were dosed for six weeks before entering Barnes Maze testing. At end of study, mice brain tissue and plasma were collected after euthanasia and perfusion. All animals will be euthanized by ketamine/xylazine injection to allow cardiac puncture and saline perfusion, which is consistent with recommendations of the AVMA Panel on Euthanasia. The collected brain was dissected to obtain hippocampus used in the biomarker analysis.

### Barnes maze assessment of spatial learning and memory

Barnes Maze testing was performed as describe by Attar et al. Briefly, in a room with visual cues on the walls, mice are introduced in a circular maze containing 20 holes where 19 holes are closed during training and all 20 holes are closed during the probe trials. On day 1 of the habituation stage, mice are placed at the center of the maze inside the start chamber. After 10 s the start chamber is removed, and mice are allowed to explore the maze for 3 min. After 3 min of exploration, the mice are guided to the target (escape) hole and allowed to stay there for 2 min. After habituation, mice undergo 3 consecutives training days, and each training day is comprised of 3 trials. Each trial starts with the mice being placed at the center of the maze inside the start chamber for 10 s. After this the start chamber is removed and mice are allowed to explore the maze for 3 min. If during this time they do not find the target hole, they are guided to it and allowed to remain inside for 1 min. At the end of each training day mice were dosed at 10 mg/kg. Next are the 24- and 48-hours probe days. During the probe days, the target hole is closed, and spatial memory of the mouse is determined during a 90 s probe trial. Mice are placed at the center of the maze inside the start chamber, after 10 s the start chamber is removed, and mice are allowed to explore the maze for 90 s. At the end of the trial, the mice are returned to the home cage and dosed with DDL-357 at 15 mg/kg. The ANY-maze software (Stoelting) was used to analyze the behavior of the mice in the Barnes Maze. Over the course of the training and probe trials, the software-calculated parameters included, but were not limited to, total distance traveled, speed, number of holes visited, latency to visiting the target/escape hole, percent time spent in the four quadrants of the maze.

### Liquid chromatography-tandem mass spectrometry

Analysis of compound levels was done at the UCLA Pasarow Mass Spectrometry Lab (PMSL; Julian Whitelegge, Ph.D., Director). A targeted LC-MS/MS assay was developed for each compound using the multiple reaction monitoring (MRM) acquisition method on a 6460 triple quadrupole mass spectrometer (Agilent Technologies) coupled to a 1290 Infinity HPLC system (Agilent Technologies) with a Phenomenex analytical column (Kinetex 1.7 µm C18 100 Å 100 ×2.1 mm). The HPLC method utilized a mixture of solvent A (99.9/1 Water/Formic Acid) and solvent B (99.9/1 Acetonitrile/Formic Acid) and a gradient was use for the elution of the compounds (min/%B: 0/1, 3/1, 19/99, 20/1, 30/1). Two fragment ions originating from each compound were monitored at specific LC retention times to ensure specificity and accurate quantification in the complex biological samples. The normalized chromatographic peak areas were determined by taking the ratio of measured chromatographic peak areas corresponding to each compound over that of the internal standard (Analyte/IS).

### Proteomics

Hippocampus of DDL-357 or vehicle treated were homogenized in lysis buffer (12 mM sodium lauroyl sarcosine, 0.5% sodium deoxycholate, 50 mM triethylammonium bicarbonate (TEAB), Halt™ Protease and Phosphatase Inhibitor Cocktail (Thermo Fisher Scientific)), the samples were reduced and alkylated with tris(2-carboxyethyl)phosphine (10 mM) and chloroacetamide (40 mM) for 30 min at 95 °C, and then digested with trypsin (20 ug) for 16 hours at 37 °C. The samples were then desalted using a modified version of Rappsilber’s protocol [ref. ^1^] and were isotopically labeled (TMT18plex Isobaric Label Reagent Set, Thermo Fisher Scientific) according to the manufacturer’s protocol to provide relative quantitation between samples. The samples were then fractionated separately via high pH reversed-phase chromatography (Pierce™ High pH Reversed-Phase Peptide Fractionation Kit) as per manufacturer’s protocol for increased proteome coverage. Aliquots of each fraction were injected onto a reverse phase nanobore HPLC column (AcuTech Scientific, C18, 1.8um particle size, 360 um – 20 cm, 150 um ID), equilibrated in solvent A (water/acetonitrile/FA, 98/2/0.1, v/v/v) and eluted (300 nL/min) with an increasing concentration of solvent B (acetonitrile/water/FA, 98/2/0.1, v/v/v: min/% F; 0/0, 5/3, 18/7, 74/12, 144/24, 153/27, 162/40, 164/80, 174/80, 176/0, 180/0) using an EASY-nLC II (Thermo Fisher Scientific). The effluent from the column was directed to a nanospray ionization source connected to a hybrid quadrupole-Orbitrap mass spectrometer (Q Exactive Plus, Thermo Fisher Scientific) acquiring mass spectra in a data-dependent mode alternating between a full scan (m/z 350-1700, automated gain control (AGC) target 3 × 106, 50 ms maximum injection time, FWHM resolution 70,000 at m/z 200) and up to 15 MS/MS scans (quadrupole isolation of charge states 2–7, isolation window 0.7 m/z) with previously optimized fragmentation conditions (normalized collision energy of 32, dynamic exclusion of 30 s, AGC target 1 × 105, 100 ms maximum injection time, FWHM resolution 35,000 at m/z 200). Raw proteomic data were searched against a Uniprot database containing the complete human proteome using SEQUEST-HT (including dynamic modifications: oxidation ( + 15.995) on M, deamidation ( + 0.984) on N/Q, and carbamidomethyl ( + 57.021), phosphorylation ( + 79.966) on S/T/Y) in Proteome Discoverer (Version 2,4, Thermo Scientific), which provided measurements of relative abundance of the identified peptides. Decoy database searching was used to generate high confidence tryptic peptides (FDR < 1%). Tryptic peptides containing amino acid sequences unique to individual proteins were used to identify and provide relative quantification between different proteins in each sample. Post-translationally modified peptides from each protein were normalized to protein abundance and peptides exhibiting a *p*-value ≤ 0.05 with a log2-fold change ≥ 0.5 were analyzed using a series of bioinformatics tools including functional protein association network analysis, comprehensive gene set enrichment gene ontology (GO) classification and pathway analysis, as well as kinase substrate enrichment analyzes^13,14,17^.

### Neurite outgrowth assay in iPSC-derived human neurons

The iPSC derived human neurons were provided by the Kornblum lab to be cultured in normal growth conditions (37 °C, 5% CO_2_) for 2–3 days. Cells were cultured in CELLSTAR uClear 384 well plates, PS, F bottom at seeding density of 5000 cells per well coated overnight with 30 ug/mL poly-D-lysine and 2 ug/mL laminin. Media was replaced every 3 days with Lonza Primary Neuron Basal Medium (PNBM) supplemented with 2 mM L-glutamine, GA-1000, and 2% NSF-1. On day 3, cells were treated with DDL357 in doses ranging from 15,000 nM to 0.76 nM for 48 h. All compounds were diluted in 0.5% DMSO and pinned to 384 well plates with 25 µL of cell media per well with pin size of 250 nL using BioMek FX (Beckman Coulter, CA). After 48 h, media was replaced twice to ensure removal of any remaining compound residues before imaging for neurite outgrowth with Calcein-AM and Hoescht. Imaging was done at UCLA Molecular Screening Shared Resource core facility (MSSR; Robert Damoiseaux, Ph.D., Director) with ImageXPress Confocal (Molecular Devices, CA) using a 10x objective. Total neurite outgrowth was measured via a MetaXpress (Molecular Devices, CA) neurite outgrowth analysis algorithm. Neurite outgrowth was set to detect cell bodies with approximate width of ≥ 20 µm and outgrowth with maximal width of 5 µm and length of ≥ 100 µm with intensity of 1000 gray scales over background. Mean neurite outgrowth per drug exposure was determined by normalizing to vehicle control (*n* = 60). Neurite outgrowth assay data were compared using a two-way ANOVA for drug effect and concentration. Internal standards were designed in each plate with controls of untreated neurons (*n* = 24) and vehicle controls of neurons treated with 0.5% DMSO (*n* = 60) since all compounds were dissolved in 0.5% DMSO - the concentration that showed no signs of cell toxicity from previous studies.

### Statistical analysis

All statistical analyzes for in vitro and in vivo studies were performed using GraphPad Prism® software; for one-way ANOVA, with level of significance set at *p* < 0.05. A two-tailed unpaired Student’s t-test was utilized where appropriate, with a *p* < 0.05 considered significant. Note than in efficacy studies, the NTg mice are not littermates of ApoE4TR-5XFAD mice (which have no NTg littermates), therefore, for statistical analysis of treatment effects, only the ApoE4TR-5XFAD groups are compared.

## Supplementary information

Supplementary information

## Data Availability

All data including raw datasets generated or analyzed during this study are included in this published article and its Supplementary Materials/Information file. The mass spectrometry proteomics data have been deposited to the ProteomeXchange Consortium via the PRIDE repository with the dataset identifier PXD060171 and 10.6019/PXD060171, and identifier PXD060173 and 10.6019/PXD060173.
