# Supplementary material for: Discovery of a small molecule secreted clusterin enhancer that improves memory in Alzheimer’s disease mice
Source: NPJ Drug Discov. 2025 May 2;2:7. doi: 10.1038/s44386-025-00009-2 (PMC12048343; doi:10.1038/s44386-025-00009-2)
Supplement: Supplementary file 1 — Supplementary information [file 44386_2025_9_MOESM1_ESM.pdf]

Supplementary Materials for

Discovery of a Small Molecule Secreted Clusterin Enhancer that Improves Memory in Alzheimer's Disease Mice

Whitaker Cohn[1,2], Jesus Campagna[1], Dongwook Wi[1], Jessica T. Lee[1], Sahiba Beniwal[1], Gazmend Elezi[2], Chunni Zhu[1], Barbara Jagodzinska[1], Julian Whitelegge[2], Robert Damoiseaux[3], Varghese John[1]

[1]The Drug Discovery Lab, Mary S. Easton Center for Alzheimer's Disease Research, Department of Neurology, David Geffen School of Medicine, 710 Westwood Plaza, University of California Los Angeles, Los Angeles, CA 90095, USA

[2]Pasarow Mass Spectrometry Laboratory, Jane and Terry Semel Institute for Neuroscience and Human Behavior, David Geffen School of Medicine, 760 Westwood Plaza, University of California Los Angeles, Los Angeles, CA 90095, USA

[3]Department of Molecular and Medical Pharmacology, 650 Charles E. Young Drive, University of California Los Angeles, Los Angeles, CA 90095, USA

TABLE OF CONTENTS

Supplementary Figures

Figure S1. *Testing of selective/pan HDAC and BET inhibitors*……………………………………3
Figure S2. *In-Silico molecular docking of (+)-JQ-1*…………………………………………..……..4
Figure S3. *Dose-response curves for ester and amide-containing (+)-JQ-1 analogs*……………..5
Figure S4. *Candidate DDL-357 cytotoxicity and hERG testing*…….………………………..…6
Figure S5. *Total and unbound DDL-357 brain levels at 3, 10, and 30 mg/Kg.* ………………….6
Figure S6. *Brain levels of potential candidate DDL-356*……………………………….……..6
Figure S7. *Target protein brain levels in ApoE4-5XFAD & 3xTg-AD mice treated with DDL-357*.....…………………………………..……………………………………………………..…7
Figure S8. *Proteomics analysis of brain tissue from DDL-357-treated 3xTg-AD mice.* …..…..…..…8
Figure S9. *sCLU, SirT1 and HN in SH-SY5Y cells and oxygen consumption rate in U-87 MG cells treated with DDL-357*….……………………………….………………………...……..9

Supplementary Tables

Table S1. *AD-relevant proteins upregulated in DDL-357-treated ApoE4TR-5XFAD mice*………10
Table S2. *AD-relevant proteins upregulated in DDL-357-treated 3xTg-AD mice*……………….11

Supporting Information: Synthesis and Analytical data……………………………..….12
NMR data for each compound……………………………………………………………...18
High Resolution Mass spec data for each compound………………………………………....38

Supplementary References……………..……………………………………...…………48

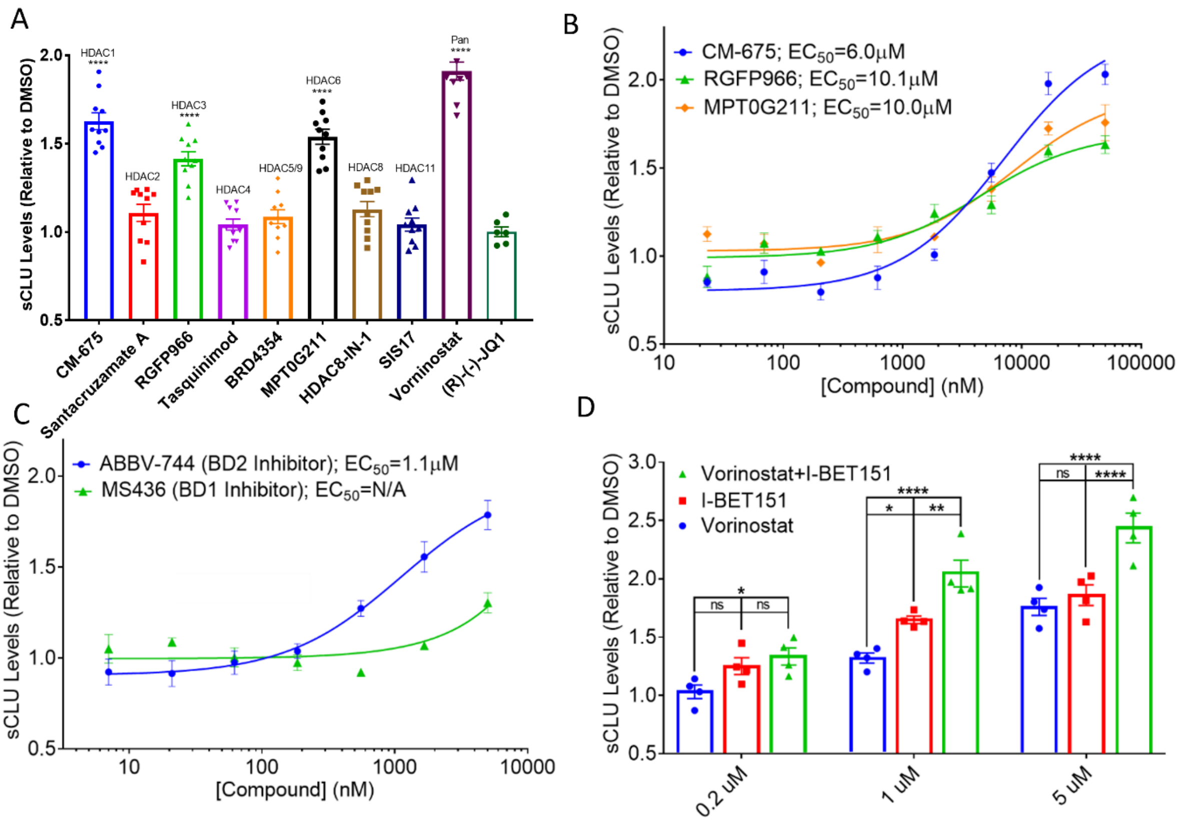

Figure S1. *Testing of selective/pan HDAC and BET inhibitors.* (A) sCLU levels following testing with isoform selective HDAC inhibitors (CM-675, santacruzamate A, RGFP966, tasquinimod, BRD4354, MPT0G211, HDAC8-IN-1, SIS17), pan HDAC inhibitor vorinostat and enantiomer (–)-JQ1at a concentration of 5 µM. (B) Dose-response curves showing sCLU levels and calculated EC_{50} values for HDAC1 inhibitor, CM-675 (6.0 µM), HDAC3 inhibitor, RGFP966 (10.1 µM), and HDAC6 inhibitor, MPT0G211 (10.0 µM), following treatment at concentrations of 2, 7, 206, 62, 185, 556, 1667 and 5000 nM. (C) Dose-response curves showing sCLU levels and calculated EC_{50} values for selective bromodomain 1 (BD1) inhibitor, MS436, and bromodomain 2 (BD2) inhibitor, ABBV-774 (1.1 µM), following treatment at concentrations of 2, 7, 206, 62, 185, 556, 1667 and 5000 nM. (D) sCLU levels following testing of vorinostat and I-BET151 independently and jointly at concentrations of 0.2, 1 and 5 µM. All statistics were performed with a one-way ANOVA (*$p \le 0.05$; **$p < 0.01$; ***$p < 0.001$; ****$p < 0.0001$).

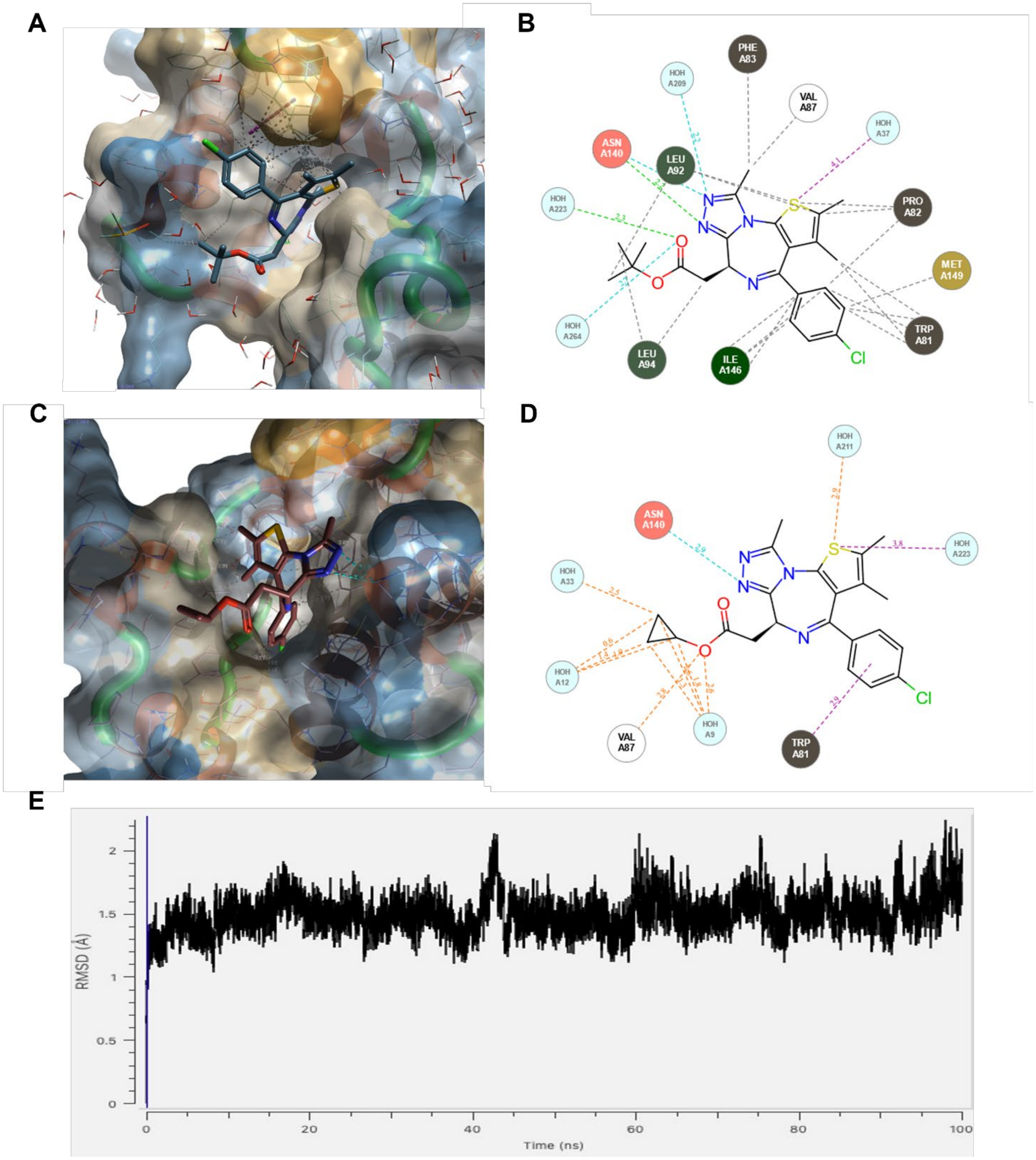

Figure S2. *In-Silico molecular docking of (+)-JQ-1 and DDL-357 to guide design of analogs with favorable protein-ligand binding energies.* (A) Molecular docking of (+)-JQ-1 in BD1 domain of BRD4 (pdb: 3MXF). (B) predicted (+)-JQ1 protein-ligand binding interactions with LF ~8.13. (C) Molecular docking of DDL-357 in BD1 domain of BRD4. (D) predicted DDL-357 protein-ligand binding interactions with LF~ 7.90, and (E) results of a 100 ns simulation (RMSD, nm; root mean square deviations) of DDL-357 in BD1 domain of BRD4 (pdb: 3MXF).

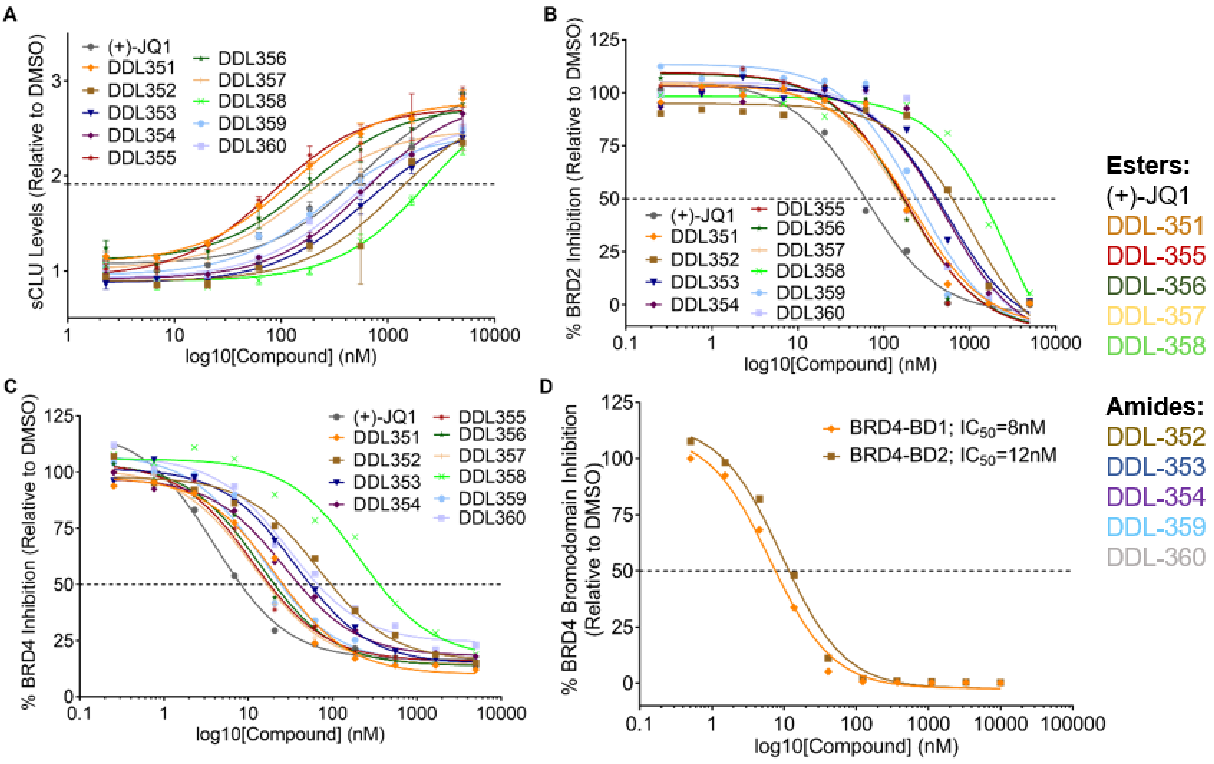

Figure S3. *Dose-response curves for ester and amide-containing (+)-JQ-1 analogs.* (A) Dose-response curves showing sCLU levels, (B) BRD2 inhibition (%), and (C) BRD4 inhibition (%) for (+)-JQ-1 and ten novel analogs, following treatment at concentrations of 2, 7, 206, 62, 185, 556, 1667 and 5000 nM. (D) Dose-response curves showing BRD4 bromodomain 1 (BD1) and bromodomain 2 (BD2) inhibition (%) and calculated IC_{50} values for lead candidate, DDL-357, following treatment at concentrations of 2, 7, 206, 62, 185, 556, 1667 and 5000 nM.

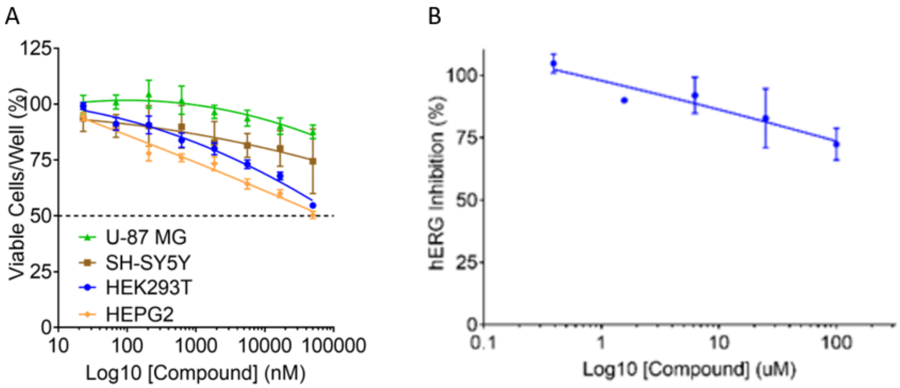

Figure S4. *Candidate DDL-357 cytotoxicity and hERG testing.* (A) Cytotoxicity in U-87 MG, SH-SY5Y, HEK293T, and HEPG2 cells following treatment with lead candidate, DDL-357, at 23, 69, 206, 617, 1852, 5556, 16667 and 50000 nM (n=4). (B) Human ether-a-go-go-related gene (hERG) potassium channel activity following treatment with lead candidate, DDL-357, at 0.39, 1.56, 6.25, 25, and 100 μM.

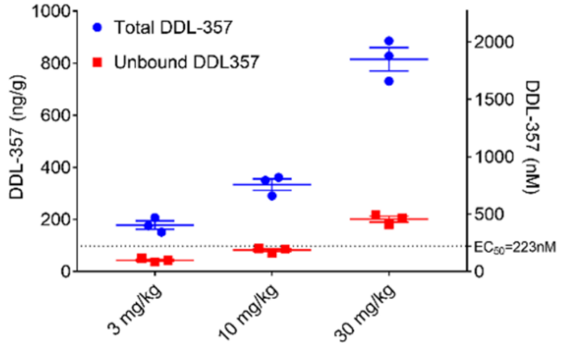

Figure S5. *Total and unbound DDL-357 brain levels at 3, 10, and 30 mg/Kg.* DDL-357 brain concentrations (total and unbound), 1 hour following oral administration at an oral dose of 3, 10, or 30 mg/kg.

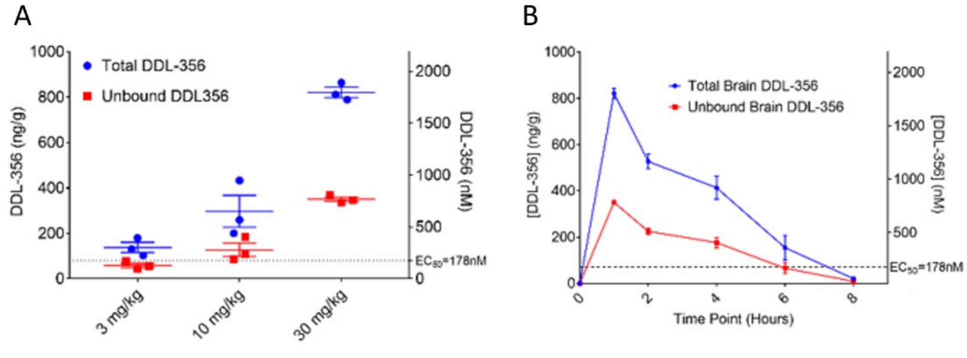

Figure S6. *Brain levels of potential candidate DDL-356*. (A) DDL-356 brain concentrations (total and unbound), 1 hour following oral administration at a dose of 3, 10, or 30 mg/kg. (B) DDL-356 brain concentrations (total and unbound), 1, 2, 4, 6 and 8 hours following oral administration at a dose of 30 mg/kg.

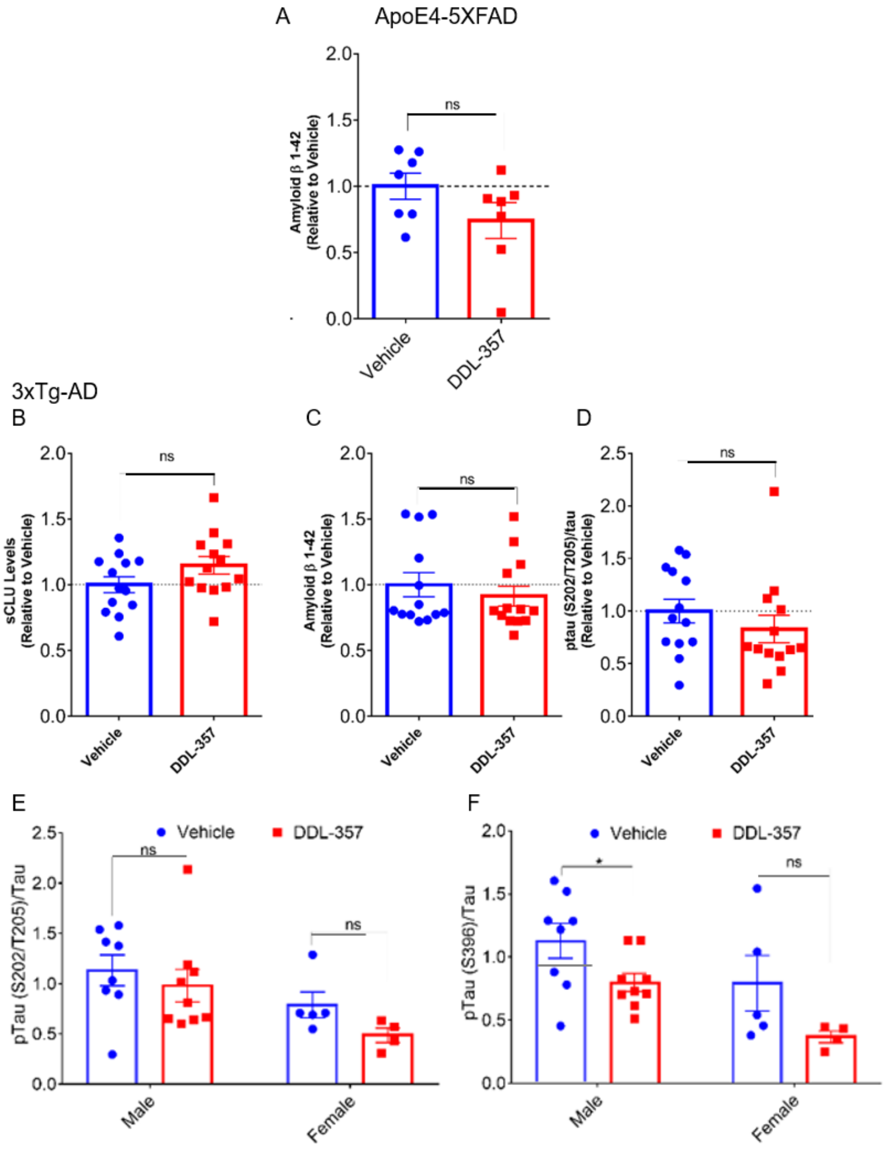

Figure S7. *Target protein brain levels in ApoE4-5XFAD and 3xTg-AD mice treated with DDL-357*. (A) Aβ1-42 levels in ApoE4TR-5XFAD brain from the subchronic, 2-week study. (B) sCLU, (C) Aβ1-42, and (D) p-tau/total tau levels at S202/T205 in 3xTg-AD brain from the 6-week chronic study. The p-tau/tau ratio for (E) S202/T205) and (F) S396 by animal sex. All mice treated with oral BID 15 mg/kg DDL-357. All statistics were performed with a two-tailed unpaired t-test (*$p < 0.05$).

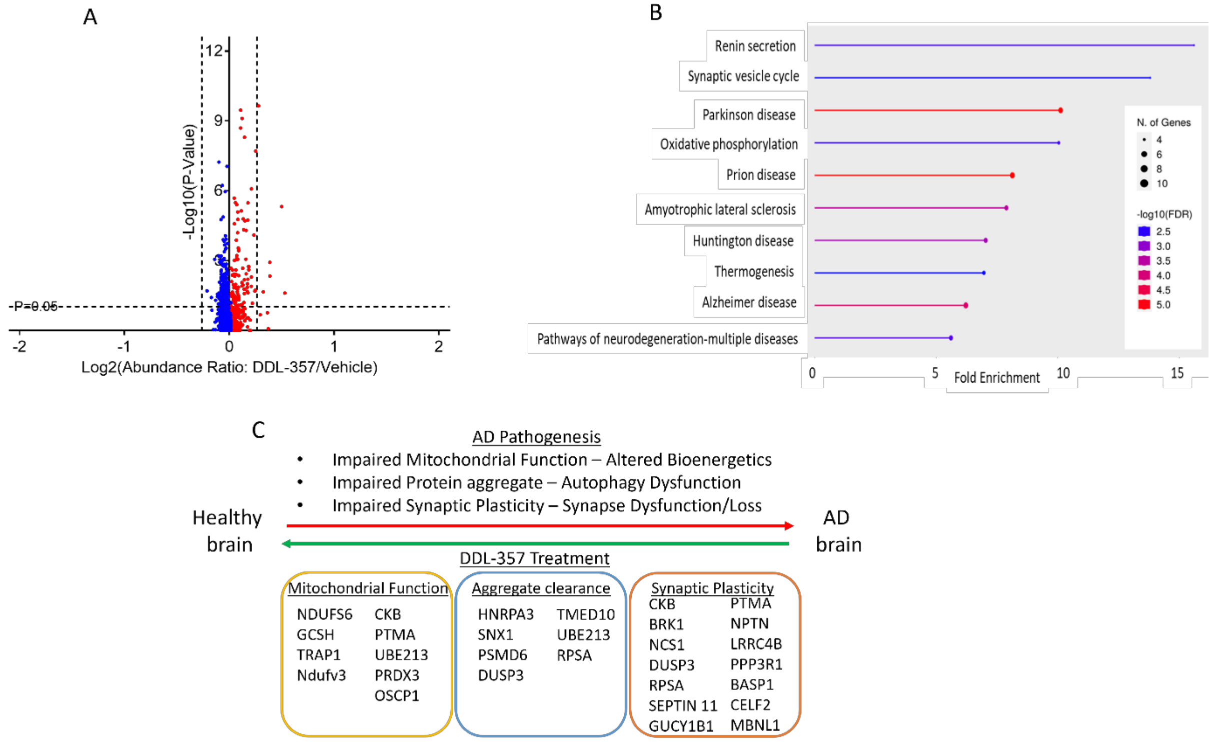

Figure S8. *Proteomics analysis of brain tissue from DDL-357-treated 3xTg-AD mice.* (A) Differences in the abundances of proteins, illustrated via volcano plot, in the hippocampus of 3xTG-AD mice following chronic 6-week oral BID administration of DDL-357 at a dose of 15 mg/kg. The−$\log_{10}$ (*p*-value) is plotted against $\log_{10}$ (abundance ratio: DDL-357/Vehicle). (B) Results of gene set pathway enrichment analysis performed on genes significantly upregulated by DDL-357 *in-vivo* (C). A schematic illustrating the DDL-357 treatment resulting in significantly upregulated proteins important for maintaining AD-relevant biological functions, including mitochondrial function, protein homeostasis, and synaptic plasticity.

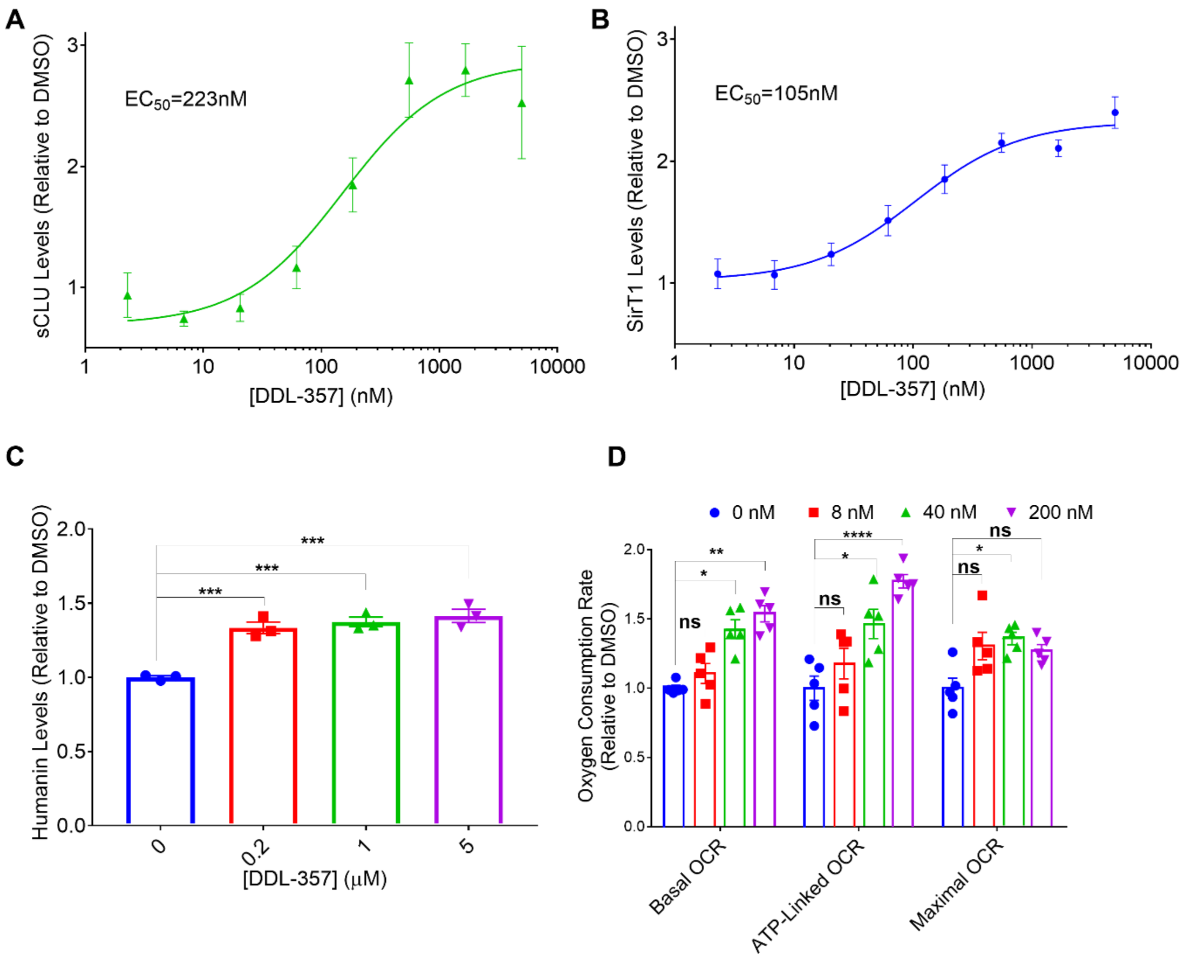

Figure S9. *sCLU, SirT1 and HN in SH-SY5Y cells and oxygen consumption rate in U-87 MG cells treated with DDL-357.* Dose-response curves for (A) sCLU and (B) SirT1 levels and the calculated EC50 values for at 2, 7, 206, 62, 185, 556, 1667 and 5000 nM treatment with DDL-357; and (C) humanin levels with 0.2, 1 and 5 μM DDL-357 in SH-SY5Y neuroblastoma cells. (D) Basal, ATP-linked, and Maximal oxygen consumption rate (OCR) in U-87 MG glioblastoma cells. All statistics were performed with a one-way ANOVA (*$p \leq 0.05$; **$p < 0.01$; ***$p < 0.001$; ****$p < 0.0001$).

Supplementary Tables

Table S1. *AD-relevant proteins upregulated in DDL-357-treated ApoE4TR-5XFAD mice.* Proteomics performed on brain tissue.

Gene	Description	Abundance Ratio (DDL357/Veh)	P-Value	Expression in AD	Biological Function [1-74] *
PSMD3	26S proteasome non-ATPase regulatory subunit 3	100.0	1.51E-13	Up	Protein Homeostasis - Proteosomal Protein [74]
TRPV2	Transient receptor potential cation channel subfamily V member 2	100.0	3.70E-14	Down	Protein Homeostasis - Receptor Promoting Aβ Phagocytosis/Clearence via Microglia
RAB8A	Ras-related protein Rab-8A	100.0	5.63E-10	Down	Protein Homeostasis - Reduces α-synuclein aggregation; Synaptic Plasticity - Postsynaptic Receptor Trafficking
BLMH	Bleomycin hydrolase	6.4	8.14E-12	N/A	Metabolic Homeostasis, Synaptic plasticity and Anti-Neuroinflammation
SMCR8	Guanine nucleotide exchange protein	4.3	2.92E-11	N/A	Protein Homeostasis - Autophagosome/Lysosome Regulator
NDUFC2	NADH:ubiquinone oxidoreductase subunit C2	4.3	1.02E-02	Down	Metabolic Homeostasis - Mitochondrial Membrane Respiratory Chain NADH Dehydrogenase (CI) Subunit
API5	Apoptosis Inhibitor-5	3.8	2.19E-03	N/A	Metabolic Homeostasis - Anti-Apoptotic Regulator of E2F1
ALCAM	Activated leukocyte cell adhesion molecule	3.5	9.02E-05	Up in Plasma	Synaptic Plasticity - Promotes Axon Elongation and Navigation [67-68]
GRPEL1	GrpE protein homolog 1, mitochondrial	2.7	1.31E-02	N/A	Protein/Metabolic Homeostasis - Mitochondrial UPR Regulator
PPP3R1	Calcineurin subunit B	2.5	4.79E-10	Down	Synaptic Plasticity - Modulates Axon Guidance, Glutamatergic Synapse, LTP, and MAPK Signaling Pathways
NDUFS5	NADH:ubiquinone oxidoreductase subunit S5	2.5	4.81E-14	Down	Metabolic Homeostasis - Mitochondrial Membrane Respiratory Chain NADH Dehydrogenase (CI) Subunit
Rab27b	Ras-related protein Rab-27B	2.2	1.38E-05	Up	Protein Homeostasis - Autophagy Regulator - Reduces α-synuclein Toxicity; Synaptic Plasticity - Promotes Presynaptic LTP
DLGAP2	Disks large-associated protein 2	2.1	5.45E-03	Down	Synaptic Plasticity - Critically Regulates Dendritic Spine Density and Morphology & Memory [24]
NPTXR	Neuronal pentraxin receptor	2.0	2.04E-13	Down	Synaptic Plasticity - Mediates Uptake of Synaptic Material and Clustering of AMPA Glutamate Receptors. Crucial for LTP [69-72]
CCT2	T-complex protein 1 subunit beta	2.0	3.98E-05	N/A	Protein Homeostasis - Protein Aggregate Receptor/Chaperone
GNAI2	Guanine nucleotide-binding protein G(i) subunit alpha-2	1.9	1.09E-06	Down	Synaptic Plasticity - Associated with LTP
EIF5A	Eukaryotic initiation factor 5A	1.9	2.11E-03	N/A	Protein Homeostasis- Inhibits TDP-43 Aggregation/UPR Regulator
PRDX3	Peroxiredoxin 3	1.8	1.74E-10	Down	Metabolic Homeostasis; Anti-NeuroInflammation - Mitochondrial Antioxidant Protein
SLC4A10	Solute carrier family 4 member 10	1.7	2.96E-08	Down	Metablolic Homeostasis - Modulates Mitochondrial/Glycolytic Enzymes; Synaptic Plasticity - Modulates Short Term Plasticity
VBP1	VHL Binding Protein 1	1.7	1.87E-03	Mutation Associated	Protein Homeostasis - Facilitates Proteasomal Protein Degradation; Synaptic Plasticity - Modulates Wnt Signaling
HTT	Huntingtin Protein	1.7	3.70E-03	Up	Synaptic Plasticity - Increases BDNF; Metabolic Homeostasis - Traffics Mitochondrial Proteins; Protein Homeostasis - Scaffold for Autophagy
SNAP29	Neuronal synaptosome associated protein 29	1.5	1.53E-06	Down	Synaptic Plasticity - Presynaptic Maintenance is Crucial for Cognitive Function; Autophagy - Mediates Autophagosome-Lysosome Fusion [71]
UBE2L3	Ubiquitin conjugating enzyme E2 L3	1.5	1.38E-02	Mutation Associated	Protein Homeostasis - Protein Ubiquitination; Anti-Neuroinflammation - Rduces IL-1β [73]
NDUFB4	NADH:Ubiquinone Oxidoreductase Subunit B4	1.5	3.10E-06	Down	Metabolic Homeostasis - Mitochondrial Membrane Respiratory Chain NADH Dehydrogenase (CI) Subunit
ITSN1	Intersectin 1	1.5	4.54E-08	N/A	Synaptic Plasticity - Activates Reelin Signaling Through VLDLR [69, 70, 72]
PLCB1	1-Phosphatidylinositol-4,5-bisphosphate phospholipase beta-1	1.4	3.28E-03	N/A	Synaptic Plasticity - Enhances endocannabinoid mobilization and NMDAR-mediated LTP
NDUFA13	NADH:Ubiquinone Oxidoreductase Subunit A13	1.4	7.18E-05	Down	Metabolic Homeostasis - Mitochondrial Membrane Respiratory Chain NADH Dehydrogenase (CI) Subunit
UQCRFS1	Cytochrome b-c1 complex subunit Rieske, mitochondrial	1.3	3.67E-06	Down	Metabolic Homeostasis - Mitochondrial Membrane Respiratory Chain Ubiquinol-Cytochrome C Oxidoreductase (CIII) Subunit

* *Supplementary References*

Supplementary Table 2. *AD-relevant proteins upregulated in DDL-357-treated 3xTg-AD mice.* Proteomics performed on brain tissue.

Gene	Description	Abundance Ratio (DDL357/Veh)	P-Value	Expression in AD	Biological Function *
HNRPA3	Isoform 2 of Heterogeneous nuclear ribonucleoprotein A3	100.0	3.58E-08	Down	Decreases C9orf72 repeat RNA levels and dipeptide-repeat protein deposition [4]
DUSP3	Dual-specificity protein phosphatase 3	100.0	3.58E-08	Down	Prevents exctotoxicityinduce neuronal death and Abeta accumulation [5-6]
NPTN	Isoform 3 of Neuroplastin	100.0	5.98E-04	Down	Regulates neuroplasticity and cognitive function [8]
LRRC4B	Leucine-rich repeat-containing protein 4B	3.2	4.72E-06	Up	Reduces demyelination, neuron damage and neuroimflamation [19-20]
MBNL1	Muscleblind-like protein 1	2.4	4.6#-6	N/A	Regulates neurate outgrouwth and brain structure [21]
TMED10	Transmembrane emp24 domain-containing protein 10	1.7	7.94E-05	Down	Reduces Abeta production and mitochondrial damage [28-29]
NDUFS6	NADH dehydrogenase [ubiquinone] iron-sulfur protein 6	1.6	1.24E-02	Down	Faciliates mitochondrial respiratory chain complex 1 function [30]
RPSA	40S ribosomal protein SA	1.5	1.84E-03	Down	Reduces p-Tau and cellular prion protein. Regulates Neuronal Morphogenesis [31-33]
GCSH	Glycine cleavage system H protein, mitochondrial	1.5	2.38E-02	N/A	Facilitates mitochondrial one-carbon metabolism [34]
BASP1	Brain acid soluble protein 1	1.5	3.26E-06	Down	Increased levels corelates to neurite outgrowth [66]
SNX1	Sorting nexin-1	1.5	1.82E-05	Associated Mutation	Facilitates endosomal trafficking and reduces Aβ production via SORLA [39-41]
NCS1	Neuronal calcium sensor 1	1.4	5.01E-05	N/A	Regulates neuron morphology, synaptic plasticity, learning and memory [26]
TRAP1	Heat shock protein 75 kDa,	1.4	3.67E-03	Down	Chaperone that supports mitochondrial dynamics, bioenergetics and antioxidant defense [38]
CELF2	CUGBP Elav-like family member 2	1.4	2.55E-02	Down	Regulates splicing in CLU transcripts [42]
PPP3R1	Calcineurin subunit B type 1	1.4	4.66E-05	Down	Strong correlation with AD and low PPP3R1, involved in axon guidance [65]
UBE213	Ubiquitin-conjugating enzyme E2 L3	1.3	7.82E-10	Associated mutation	Faccilitates mitophagy and ubiquitin-proteasome system-mediated protein degradation [49-51]
PRDX3	Thioredoxin-dependent peroxide reductase, mitochondrial	1.3	8.66E-03	Down	Supports neurite outgrouwth and antioxidant defense [43-44]
SEPTIN	Septin-11	1.3	7.23E-06	Down	Regulates splicing in CLU transcripts [42]
BRK1	Protein BRICK1	1.3	3.45E-10	N/A	Supports WAVE complex-mediated actin reorganization and synapse function [46]
PSMD6	26S proteasome non-ATPase regulatory subunit 6	1.2	3.41E-04	Down	Facilitates ubiquitin-proteasome system-mediated protein degradation [52]
GUCY1B1	Guanylate cyclase soluble unit beta-1	1.2	3.04E-03	Down	Increases neuronal activity, neuroprotection and cognitive performance [53-54]
PTMA	Prothymosin alpha	1.2	4.35E-03	Up (protective)	Increases oxidative phosphorylation, neurogenesis and memore [55-57]
OSCP1	Protein OSCP1	1.2	1.36E-02	Down	Protects against Aβ-induced mitochondrial dysfunction and synaptic injury [63-64]
Ndufv3	NADH dehydrogenase [ubiquinone] flavoprotein 3	1.2	4.53E-02	Down	Facilitates mitochondrial one-carbon metabolism [34]
CKB	Creatine kinase B-type	1.2	1.69E-05	Down	Increases ATP production, protects agains Abeta-induced axonal degeneration and memory loss [60-62]

* *Supplementary References*

Supporting Information: Synthesis and Analytical Data

The chemicals used for synthesis were purchased from Aldrich Chemical Co., Sigma Chemical Co., Enamine, A2B Chemical CO., Sigma Aldrich, and 1-ClickChemistry, and used as received without purification. The experiments were conducted using a commercially available continuous-flow system (Syrris Asia). Column chromatography was carried out using a CombiFlash Rf 200 instrument and prepacked silica gel (300–400 mesh) cartridges. Analytical thin–layer chromatographic separations were carried out on silica gel (60 Å particle size, 250 μm thickness, F-254, Silicycle) coated glass plates; spots were visualized with UV light. NMR spectra were recorded using a 400 MHz Bruker spectrometer. Chemical shifts are reported in parts per million (ppm, δ) relative to residual ^{1}H resonance of the solvent CDCl3 at 7.26 ppm. C NMR chemical shifts are reported relative to the central line of CDCl3 at 77.16 ppm. High-resolution DART-MS spectra were collected on Thermo Scientific LTQ Orbitrap XL^{TM} Hybrid FT Mass spectrometer equipped with an API ion source, LTQ XL linear ion trap, C-trap, HCD collision cell, and orbitrap mass analyzer. Ions were selected in the linear ion trap and fragmented either in the ion trap (CID) or the new collision cell (HCD). For HCD (Higher Energy Collisional Dissociation) ions were passed through the C-trap into the gas-filled collision cell. Normalized Collision Energy in HCD MS/MS experiments provided reproducible data from instrument to instrument.

DDL-351: *Cyclopentyl (S)-2-(4-(4-chlorophenyl)-2,3,9-trimethyl-6H-thieno[3,2-f][1,2,4]triazolo[4,3-a][1,4]diazepin-6-yl)acetate*

A 25 mL round bottom flask equipped with a stir bar was charged with (S)-2-(4-(4-chlorophenyl)-2,3,9-trimethyl-6H-thieno[3,2-f][1,2,4]triazolo[4,3-a][1,4]diazepin-6-yl)acetic acid (51.24 mg, 1 equiv), pentanol (32.23 mg, 3 equiv), and 4-Dimethylaminopyridine (30.42 mg, 2 equiv). The starting materials and reagents were dissolved in 700 μL of methylene chloride and brought to 0 °C in an ice bath. Diisopropylcarbodiimide (31.42 mg, 2 equiv) was added dropwise over the course of 30 min. The reaction was slowly brought to room temperature. After 24 h, insoluble urea product was visible, and the reaction mixture was filtered through a filter funnel and concentrated in vacuo. The crude product was purified via flash column chromatography. A mobile phase of hexanes: ethyl acetate (time/% ethyl acetate: 0/0, 2/0, 7/50, 15/100, 20/0) was first utilized to elute any existing impurities, and the product was eluted in the DCM:MeOH mobile phase (time/%MeOH: 0/0, 4/0, 35/15, 42/20, 45/50, 50/0). The fractions that corresponded to the interest peaks were dried in a speed vacuum to yield DDL 351 (47.76 mg, 79.67 %). δ ^{1}H NMR (400 MHz, CDCl3) δ 7.41 (m, 2H), 7.32 (m, 2H), 5.25 (m, 1H), 4.12 (q, J = 6.47 Hz, 1H), 3.57 (m, 1H), 2.68 (s, 3H), 2.41 (s, 3H), 1.90 (m, 2H), 1.77 (m, 2H), 1.69 (s, 3H), 1.60 (m, 2H), 1.25 (t, J = 7.06 Hz, 2H). ^{13}C NMR ($CDCl_3$) δ 171.35, 163.75, 155.47, 149.89, 136.79, 136.69, 132.,35, 130.88, 130.81, 130.41, 129.88, 128.74, 60.42, 53.96, 37.19, 32.77, 23.82, 14.42, 14.25, 13.16, 11.89. HRMS-ESI (m/z) $[M+H]^+$ calcd for $C_{24}H_{25}ClN_4O_2S$, 469.14595 found 469.16668.

DDL-352: *(S)-2-(4-(4-chlorophenyl)-2,3,9-trimethyl-6H-thieno[3,2-f][1,2,4]triazolo[4,3-a][1,4]diazepin-6-yl)-1-(pyrrolidin-1-yl)ethan-1-one*

(S)-2-(4-(4-chlorophenyl)-2,3,9-trimethyl-6H-thieno[3,2-f][1,2,4]triazolo[4,3-a][1,4]diazepin-6-yl)acetic acid (80.00 mg, 0.199 mmol, 1 equiv.) in 3 mL of anhydrous DMF was added into a 250mL round bottom flask. Pyrrolidine (32.68 uL, 0.398 mmol, 2 equiv.) was added into the tube followed by the addition of EDAC (76.5 mg, 0.398 mmol, 2 equiv.), HOBt (29.67 mg, 0.219 mmol, 1.1 equiv.), and N-N, Diisopropylethylamine (0.104 mL, 0.597 mmol, 3 equiv.), and the resulting reaction mixture were stirred at room temperature overnight. After stirring overnight, the solution changed colors to light brownish. FIA was performed to check the reaction. The reaction mixture was dried and reconstituted using 20 mL of DCM, transferred to a separatory funnel, and the organic phase was washed with brine (2 x 20 mL), dried with anhydrous Na_2SO_4, filtered, concentrated, and evaporated. The resultant crude compound was purified by using a 4 g silica flash column, eluted with DCM: MeOH (time/% MeOH: 5/0, 20/10, 35/20). The fractions that were corresponding to the interest peaks were dried in a speed vacuum to yield DDL 352 (49.66 mg, Viscous white solid, 54.9%). ^{1}H NMR (400 MHz, $CDCl_3$) δ 7.39 (d, *J* = 8 Hz, 2H), 7.31 (d, *J* = 8 Hz, 2H), 4.80 (t, *J* = 4 Hz 1H), 3.69 – 3.49 (m, 4H), 2.95 (s, 3H), 2.88 (d, *J* = 0.6 Hz, 2H), 2.66 (s, 3H), 2.39 (s, 3H), 2.09 – 1.85 (m, 4H). ^{13}C NMR ($CDCl_3$) δ 169.05, 163.76, 162.60, 155.97, 149.86, 136.96, 136.62, 132.27, 130.99, 130.67, 130.61, 129.89, 128.70, 54.41, 46.99, 45.84, 37.01, 36.53, 31.49, 26.14, 24.57, 14.40, 13.11, 11.88. HRMS-ESI (m/z) $[M+H]^+$ calcd for $C_{23}H_{24}ClN_5OS$, 454.14629 found 454.08334.

DDL-353: *(S)-2-(4-(4-chlorophenyl)-2,3,9-trimethyl-6H-thieno[3,2-f][1,2,4]triazolo[4,3-a][1,4]diazepin-6-yl)-N-cyclopentylacetamide*

(S)-2-(4-(4-chlorophenyl)-2,3,9-trimethyl-6H-thieno[3,2-f][1,2,4]triazolo[4,3-a][1,4]diazepin-6-yl)acetic acid (80.00 mg, 0.199 mmol, 1 equiv.) in 3 mL of anhydrous DMF was added into a 250mL round bottom flask. Cyclopentanamine (39.4 uL, 0.398 mmol, 2 equiv.) was added into the tube followed by the addition of EDAC (76.5 mg, 0.398 mmol, 2 equiv.), HOBt (29.67 mg, 0.219 mmol, 1.1 equiv.), and N-N, Diisopropylethylamine (0.104 mL, 0.597 mmol, 3 equiv.), and the resulting reaction mixture were stirred at room temperature overnight. After stirring overnight, the solution changed colors to light brownish. FIA was performed to check the reaction. The reaction mixture was dried and reconstituted using 20 mL of DCM, transferred to a separatory funnel, and the organic phase was washed with brine (2 x 20 mL), dried with anhydrous Na_2SO_4, filtered, concentrated, and evaporated. The resultant crude compound was purified by using a 4 g silica flash column, eluted with DCM: MeOH (time/% MeOH: 5/0, 20/10, 35/20). The fractions that were corresponding to the interest peaks were dried in a speed vacuum to yield DDL 353 (45 mg, Viscous white solid, 48.3 %). ^{1}H NMR (400 MHz, $CDCl_3$) δ 7.39 (d, *J* = 8.6 Hz, 2H), 7.32 (d, *J* = 8.7 Hz, 2H), 4.60 (t, *J* = 8 Hz 1H), 4.26-4.18 (m, 1H), 3.53-3.27 (m, 2H), 2.66 (s, 3H), 2.39 (s, 3H), 2.04-1.89 (m, 2H), 1.73-1.68 (m, 2H), 1.66 (s, 1H), 1.65-1.43 (m, 4H). ^{13}C NMR ($CDCl_3$) δ 170.11, 163.99, 155.80, 149.97, 136.92, 136.82, 132.33, 131.05, 130.91, 130.60, 129.93, 128.86,

60.52, 54.76, 53.55, 51.43, 39.77, 33.36, 33.04, 23.93, 23.91, 14.50, 13.21, 11.99. HRMS-ESI (m/z) $[M+H]^+$ calcd for $C_{24}H_{26}ClN_5OS$, 468.16194 found 468.25001.

DDL-354: *Isopropyl (S)-(2-(4-(4-chlorophenyl)-2,3,9-trimethyl-6H-thieno[3,2-f][1,2,4]triazolo[4,3-a][1,4]diazepin-6-yl)acetyl)glycinate*

(S)-2-(4-(4-chlorophenyl)-2,3,9-trimethyl-6H-thieno[3,2-f][1,2,4]triazolo[4,3-a][1,4]diazepin-6-yl)acetic acid (80.00 mg, 0.199 mmol, 1 equiv.) in 3 mL of anhydrous DMF was added into a 250mL round bottom flask. Isopropyl glycinate HCl (61.13 mg, 0.398 mmol, 2 equiv.) was added into the tube followed by the addition of EDAC (76.5 mg, 0.398 mmol, 2 equiv.), HOBt (29.67 mg, 0.219 mmol, 1.1 equiv.), and N-N, Diisopropylethylamine (0.208 mL, 1.194 mmol, 6 equiv.), and the resulting reaction mixture were stirred at room temperature overnight. After stirring overnight, the solution changed colors to light brownish. FIA was performed to check the reaction. The reaction mixture was dried and reconstituted using 20 mL of DCM, transferred to a separatory funnel, and the organic phase was washed with brine (2 x 20 mL), dried with anhydrous Na_2SO_4, filtered, concentrated, and evaporated. The resultant crude compound was purified by using a 4 g silica flash column, eluted with DCM: MeOH (time/% MeOH: 5/0, 20/10, 35/20). The fractions that were corresponding to the interest peaks were dried in a speed vacuum to yield DDL 354 (48 mg, Viscous white solid, 48.2 %). ^1H NMR (400 MHz, $CDCl_3$) δ 7.42 (d, *J* = 8.6 Hz, 2H), 7.32 (d, *J* = 8.7 Hz, 2H), 5.06 (hept, *J* = 6.3 Hz, 1H), 4.62 (t, *J* = 6.9 Hz, 1H), 4.16 – 4.06 (m, 1H), 3.95 (dd, *J* = 18.2, 4.9 Hz, 1H), 3.60 (dd, *J* = 14.6, 6.8 Hz, 1H), 3.45 (dd, *J* = 14.6, 7.1 Hz, 1H), 2.66 (s, 3H), 2.39 (s, 3H), 1.67 (s, 3H), 1.24 (d, *J* = 6.3 Hz, 6H). ^{13}C NMR ($CDCl_3$) δ 170.84, 169.29, 164.08, 155.74, 136.92, 136.73, 132.30, 131.08, 130.92, 130.62, 130.05, 128.82, 69.28, 54.44, 44.40, 41.94, 39.14, 21.97, 21.89, 14.51, 13.21, 11.95. HRMS-ESI (m/z) $[M+H]^+$ calcd for $C_{24}H_{26}ClN_5O_3S$, 500.15176 found 500.33335.

DDL-355: *Cyclobutyl (S)-2-(4-(4-chlorophenyl)-2,3,9-trimethyl-6H-thieno[3,2-f][1,2,4]triazolo[4,3-a][1,4]diazepin-6-yl)acetate*

A 25 mL round bottom flask equipped with a stir bar was charged with (S)-2-(4-(4-chlorophenyl)-2,3,9-trimethyl-6H-thieno[3,2-f][1,2,4]triazolo[4,3-a][1,4]diazepin-6-yl)acetic acid (51.13 mg, 1 equiv), cyclobutanol (26.98 mg, 3 equiv), and 4-Dimethylaminopyridine (31.48 mg, 2 equiv). The starting materials and reagents were dissolved in of methylene chloride (800 µL) and was brought to 0 °C in an ice bath. A solution of diisopropylcarbodiimide (31.48 mg, 2 equiv) in methylene chloride (200 µL) was added dropwise over the course of 30 min. The reaction was slowly brought to room temperature. After 24 h, the insoluble urea product was visible, and the reaction mixture was filtered through a filter funnel and concentrated in vacuo. The crude product was purified via flash column chromatography. A mobile phase of hexanes: ethyl acetate (time/% ethyl acetate: 0/0, 2/0, 7/50, 15/100, 20/0) was first utilized to elute any existing impurities, and the product was

eluted in the DCM: MeOH mobile phase (time/%MeOH: 0/0, 4/0, 35/15, 42/20, 45/50, 50/0). The fractions that corresponded to the interest peaks were dried in a speed vacuum to yield DDL 355 (48.82 mg, White solid, 84 %). ^{1}H NMR (300 MHz, $CDCl_3$) δ 7.50 – 7.32 (m, 4H), 5.16 – 5.04 (m, 1H), 4.64 (dd, J = 7.5, 6.6 Hz, 1H), 3.70 – 3.59 (m, 2H), 2.73 (s, 3H), 2.45 (s, 3H), 2.26 – 2.11 (m, 2H), 1.93 – 1.79 (m, 1H), 1.73 (s, 3H), 1.69 – 1.59 (m, 1H), 1.30 (dt, J = 7.1, 4.1 Hz, 1H), 0.90 (dd, J = 14.6, 6.8 Hz, 1H). ^{13}C NMR ($CDCl_3$) δ 171.15, 163.88, 155.51, 150.00, 136.88, 136.79, 132.44, 130.99, 130.83, 130.50, 129.96, 128.82, 69.37, 53.95, 36.95, 30.49, 30.39, 14.51, 13.76, 13.22, 11.98. HRMS-ESI (m/z) $[M+H]^+$ calcd for $C_{23}H_{23}ClN_4O_2S$, 455.13030 found 455.25001.

DDL-356: *Oxetan-3-yl (S)-2-(4-(4-chlorophenyl)-2,3,9-trimethyl-6H-thieno[3,2-f][1,2,4]triazolo[4,3-a][1,4]diazepin-6-yl)acetate*

A 25 mL round bottom flask equipped with a stir bar was charged with (S)-2-(4-(4-chlorophenyl)-2,3,9-trimethyl-6H-thieno[3,2-f][1,2,4]triazolo[4,3-a][1,4]diazepin-6-yl)acetic acid (150 mg, 1 equiv), oxetane-3-ol (71.4 uL, 3 equiv), and 4-Dimethylaminopyridine (91.62 mg, 2 equiv). The starting materials and reagents were solubilized in methylene chloride (2 mL) and were brought to 0 °C in an ice bath. A solution of diisopropylcarbodiimide (117.43 uL, 2 equiv) in methylene chloride (1 mL) was added, and the reaction was slowly brought to room temperature. After 24 h, the insoluble urea product was visible, and the reaction mixture was filtered through a filter funnel. The filtrate was washed with sodium bicarbonate to remove unreacted acid (2 x 10mL) followed by an extraction using DCM, and the solution was washed with brine (2 x 10 mL), and the crude product was purified via flash column chromatography. A mobile phase of hexanes: ethyl acetate (time/% ethyl acetate: 0/60, 5/70, 25/100, 30/0) was first utilized to elute any existing impurities, and the product was eluted in the DCM: MeOH mobile phase (time/% methanol: 0/0, 5/0, 20/10, 35/20, and 40/0) to afford DDL 356 (129mg, 75.4%, white powder). ^{1}H NMR (400 MHz, DMSO) δ 7.46 (d, J = 8.7 Hz, 2H), 7.39 (d, J = 8.6 Hz, 2H), 5.40 (t, J = 5.0 Hz, 1H), 4.80 – 4.44 (m, 5H), 3.47 (d, J = 14.0 Hz, 2H), 2.57 (s, 3H), 2.37 (s, 3H), 1.59 (s, 3H). ^{13}C NMR ($CDCl_3$) δ 171.05, 164.17, 155.27, 150.11, 137.02, 136.65, 132.43, 131.00, 130.98, 130.40, 129.92, 128.88, 68.34, 60.51, 53.78, 36.75, 21.16, 14.54, 13.23, 11.97. HRMS-ESI (m/z) $[M+H]^+$ calcd for $C_{22}H_{21}ClN_4O_3S$, 457.10957 found 457.33334.

DDL-357: *Cyclopropyl (S)-2-(4-(4-chlorophenyl)-2,3,9-trimethyl-6H-thieno[3,2-f][1,2,4]triazolo[4,3-a][1,4]diazepin-6-yl)acetate*

A 25 mL round bottom flask equipped with a stir bar was charged with (S)-2-(4-(4-chlorophenyl)-2,3,9-trimethyl-6H-thieno[3,2-f][1,2,4]triazolo[4,3-a][1,4]diazepin-6-yl)acetic acid (51.24 mg, 1 equiv), propanol (21.73 mg, 3 equiv), and 4-Dimethylaminopyridine (30.48 mg, 2 equiv). The starting materials and reagents were dissolved in methylene chloride (800 μL) and was brought to 0 °C in an ice bath. A solution of diisopropylcarbodiimide (31.48 mg, 2 equiv) in methylene chloride (200 μL) was added dropwise over the course of 30 min. The reaction was slowly brought to room temperature. After 24 h, the insoluble urea product was visible, and the reaction mixture

was filtered through a filter funnel and concentrated in vacuo. The crude product was purified via flash column chromatography. A mobile phase of hexanes: ethyl acetate (time/% ethyl acetate: 0/0, 2/0, 7/50, 15/100, 20/0) was first utilized to elute any existing impurities, and the product was eluted in the DCM: MeOH mobile phase (time/%MeOH: 0/0, 4/0, 35/15, 42/20, 45/50, 50/0). The fractions that corresponded to the interest peaks were dried in a speed vacuum to yield DDL 357 (55.12 mg, white solid, 91 %). δ ^{1}H NMR (400 MHz, CDCl3) δ^{1}H NMR (300 MHz, $CDCl_3$) δ 7.50 – 7.32 (m, 4H), 4.65 (t, *J* = 7.0 Hz, 1H), 4.30 – 4.17 (m, 1H), 3.63 (d, *J* = 7.1 Hz, 2H), 2.73 (s, 3H), 2.46 (d, *J* = 0.8 Hz, 3H), 1.73 (s, 3H), 0.95 – 0.72 (m, 4H). ^{13}C NMR ($CDCl_3$) δ 172.68, 163.96, 155.43, 150.02, 136.91, 136.74, 132.42, 131.01, 130.88, 130.49, 129.96, 128.83, 53.93, 49.36, 36.91, 14.52, 13.23, 11.98, 5.25. HRMS-ESI (m/z) $[M+H]^+$ calcd for $C_{22}H_{22}ClN_4O_2S$, 441.11465 found 441.16668.

DDL-358: *Butyl (S)-2-(4-(4-chlorophenyl)-2,3,9-trimethyl-6H-thieno[3,2-f][1,2,4]triazolo[4,3-a][1,4]diazepin-6-yl)acetate*

A 10 mL round bottom flask equipped with a stir bar was charged with (S)-2-(4-(4-chlorophenyl)-2,3,9-trimethyl-6H-thieno[3,2-f][1,2,4]triazolo[4,3-a][1,4]diazepin-6-yl)acetic acid (67 mg, 1 equiv), 1-butanol (28 mg, 3 equiv), and 4-Dimethylaminopyridine (33 mg, 2 equiv). The starting materials and reagents were dissolved in of methylene chloride (1 mL) and was brought to 0 °C in an ice bath. A solution of diisopropylcarbodiimide (31.55 mg, 2 equiv) in methylene chloride (200 uL) was added dropwise over the course of 30 min. The reaction was slowly brought to room temperature. After 24 h, insoluble urea product was visible, and the reaction mixture was filtered through a filter funnel and concentrated in vacuo. The crude product was purified via flash column chromatography. A mobile phase of hexanes: ethyl acetate (time/% ethyl acetate: 0/0, 2/0, 7/50, 15/100, 20/0) was first utilized to elute any existing impurities, and the product was eluted in the DCM: MeOH mobile phase (time/%MeOH: 0/0, 4/0, 35/15, 42/20, 45/50, 50/0). The fractions that corresponded to the interest peaks were dried in a speed vacuum to yield DDL 358 (13 mg, white solid, 23 %). δ ^{1}H NMR (400 MHz, CDCl3) δ 7.44 – 7.30 (m, 4H), 4.63 (t, J = 6.7 Hz, 1H), 4.18 (t, J = 6.6 Hz, 2H), 3.62 (d, J = 1.4 Hz, 2H), 2.73 (s, 3H), 2.42 (s, 3H), 1.69 (s, 3H), 1.68 – 1.62 (m, 2H), 1.47 – 1.34 (m, 2H), 0.94 (t, J = 7.4 Hz, 3H). ^{13}C NMR ($CDCl_3$) δ 171.80, 163.88, 155.53, 150.00, 136.89, 136.76, 132.42, 131.00, 130.85, 130.51, 129.97, 128.81, 64.76, 53.97, 37.04, 30.82, 19.28, 14.51, 13.85, 13.23, 11.97. HRMS-ESI (m/z) $[M+H]^+$ calcd for $C_{23}H_{25}ClN_4O_2S$, 457.14595 found 457.25001.

DDL-359: *Isopropyl (2-((S)-4-(4-chlorophenyl)-2,3,9-trimethyl-6H-thieno[3,2-f][1,2,4]triazolo[4,3-a][1,4]diazepin-6-yl)acetyl)-L-alaninate*

(S)-2-(4-(4-chlorophenyl)-2,3,9-trimethyl-6H-thieno[3,2-f][1,2,4]triazolo[4,3-a][1,4]diazepin-6-yl)acetic acid (80.00 mg, 0.199 mmol, 1 equiv.) in 3 mL of anhydrous DMF was added into a 250 mL round bottom flask. isopropyl L-alaninate (52.2 mg, 0.398 mmol, 2 equiv.) was added into the

tube followed by the addition of 4-Dimethylaminopyridine (48.62 mg, 0.398 mmol, 2 equiv.), and DIC (50.22 µL, 0.398 mmol, 2 equiv.), and the resulting reaction mixture were stirred at room temperature overnight. After stirring overnight, the solution changed colors to light brownish. FIA was performed to check the reaction. The reaction mixture was dried and reconstituted using 20 mL of DCM, transferred to a separatory funnel, and the organic phase was washed with brine (2 x 20 mL), dried with anhydrous Na_2SO_4, filtered, concentrated, and evaporated. The resultant crude compound was purified by using a 4 g silica flash column, eluted with DCM: MeOH (time/% MeOH: 5/0, 20/10, 35/20). The fractions that were corresponding to the interest peaks were dried in a speed vacuum to yield DDL 359 (48 mg, Viscous white solid, 48.2 %). ^{1}H NMR (400 MHz, $CDCl_3$) δ 7.39 (d, *J* = 8.6 Hz, 2H), 7.31 (d, *J* = 8.8 Hz, 2H), 5.02 (hept, *J* = 6.3 Hz, 1H), 4.60 (t, J = 6.7 Hz, 1H), 4.54 (q, *J* = 7.2 Hz, 1H), 3.61 – 3.37 (m, 2H), 2.66 (s, 3H), 2.39 (s, 3H), 1.66 (s, 3H), 1.45 (d, *J* = 7.2 Hz, 3H), 1.21 (dd, *J* = 9.5, 6.2 Hz, 6H). ^{13}C NMR ($CDCl_3$) δ 172.27, 170.17, 163.97, 155.75, 149.98, 136.91, 136.76, 132.32, 131.04, 130.90, 130.59, 129.93, 128.82, 69.08, 54.37, 48.70, 39.12, 21.81, 21.76, 18.62, 14.51, 13.20, 11.95. HRMS-ESI (m/z) $[M+H]^+$ calcd for $C_{25}H_{26}ClN_5O_3S$, 514.16741 found 514.50001.

DDL-360: *(S)-2-(4-(4-chlorophenyl)-2,3,9-trimethyl-6H-thieno[3,2-f][1,2,4]triazolo[4,3-a][1,4]diazepin-6-yl)-N-hydroxyacetamide*

To a mixture of (S)-2-(4-(4-chlorophenyl)-2,3,9-trimethyl-6H-thieno[3,2-f][1,2,4]triazolo[4,3-a][1,4]diazepin-6-yl)acetic acid (0.0998mmol, 1 Equiv.), EDC (0.1497mmol, 1.5 Equiv.), and HOBt (0.1497mmol, 1.5 Equiv.) in anhydrous DMF were added DIPEA (0.299mmol, 3 Equiv.). The mixture was stirred at RT for 10 min followed by the addition of hydroxyl amine HCl (0.1497mmol, 1.5 Equiv.). The mixture was stirred at rt for 24hr. The resultant crude compound was purified by using a 4 g silica flash column, eluted with DCM: MeOH (time/% MeOH: 0/0, 3/0, 20/10, and 30/100). The fractions that were corresponding to the interest peaks were dried in a speed vacuum to yield DDL 360 (13.41 mg, white solid, 32.4%). ^{1}H NMR (400 MHz, $CDCl_3$) δ 7.46 (d, *J* = 8 Hz, 2H), 7.41 (d, *J* = 8 Hz, 2H), 4.46 (t, *J* = 8 Hz, 1H), 3.09 (d, *J* = 8 Hz), 2.56 (s, 3H), 2.38 (s, 3H), 1.59 (s, 3H), 1.20 (s, 1H). ^{13}C NMR ($CDCl_3$) δ 173.29, 168.07, 164.48, 137.09, 136.48, 131.30, 131.17, 130.73, 130.07, 129.00, 128.88, 54.13, 14.51, 13.24, 11.89. HRMS-ESI (m/z) $[M+H]^+$ calcd for $C_{19}H_{18}ClN_5O_2S$, 416.09425 found 416.33334.

NMR data for each compound

DDL-351: *Cyclopentyl (S)-2-(4-(4-chlorophenyl)-2,3,9-trimethyl-6H-thieno[3,2-f][1,2,4]triazolo[4,3-a][1,4]diazepin-6-yl)acetate ^{1}H NMR*

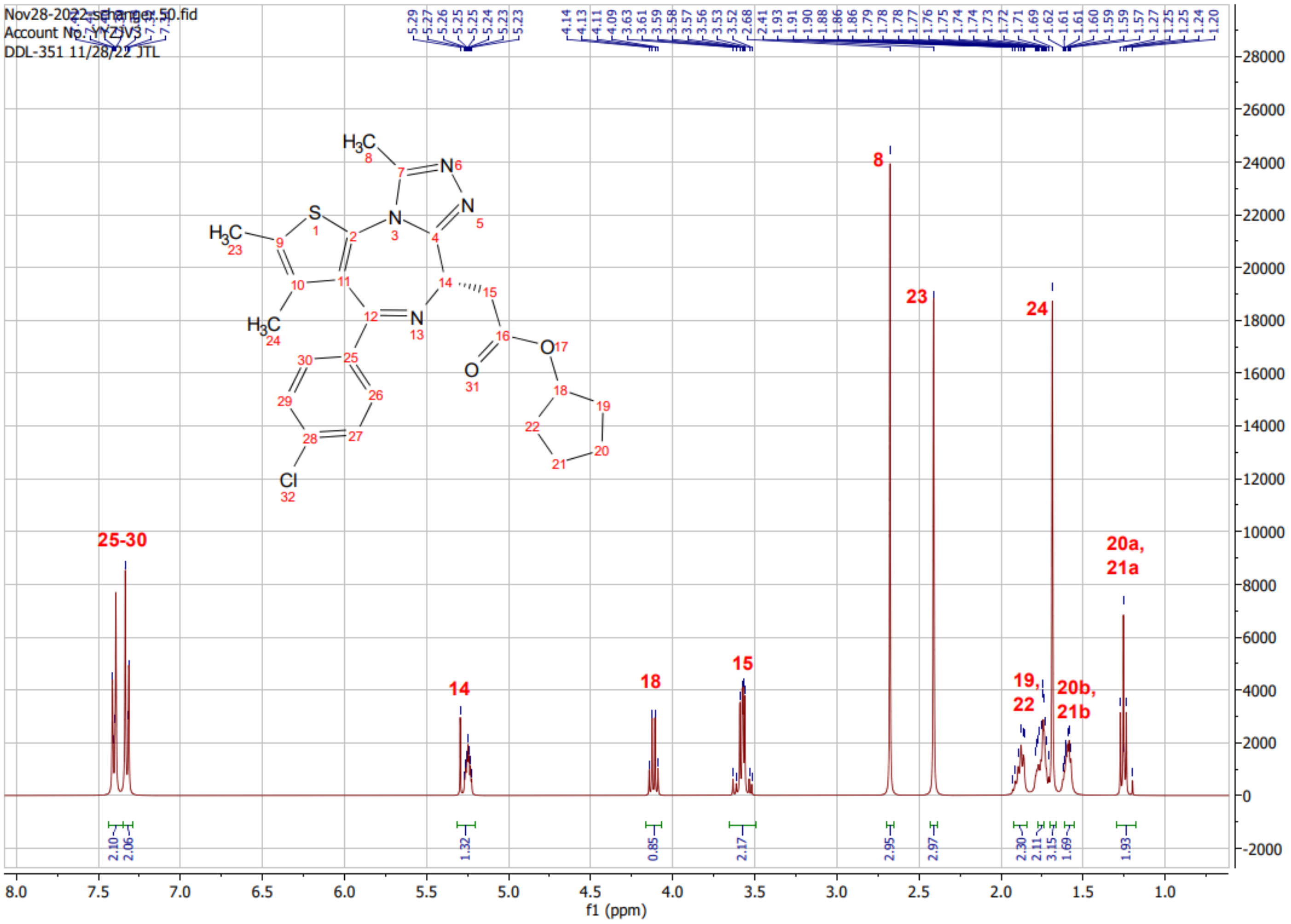

δ ^{1}H NMR (400 MHz, CDCl3) δ 7.41 (m, 2H), 7.32 (m, 2H), 5.25 (m, 1H), 4.12 (q, J = 6.47 Hz, 1H), 3.57 (m, 1H), 2.68 (s, 3H), 2.41 (s, 3H), 1.90 (m, 2H), 1.77 (m, 2H), 1.69 (s, 3H), 1.60 (m, 2H), 1.25 (t, J = 7.06 Hz, 2H).

DDL-351: *Cyclopentyl (S)-2-(4-(4-chlorophenyl)-2,3,9-trimethyl-6H-thieno[3,2-f][1,2,4]triazolo[4,3-a][1,4]diazepin-6-yl)acetate ^{13}C NMR*

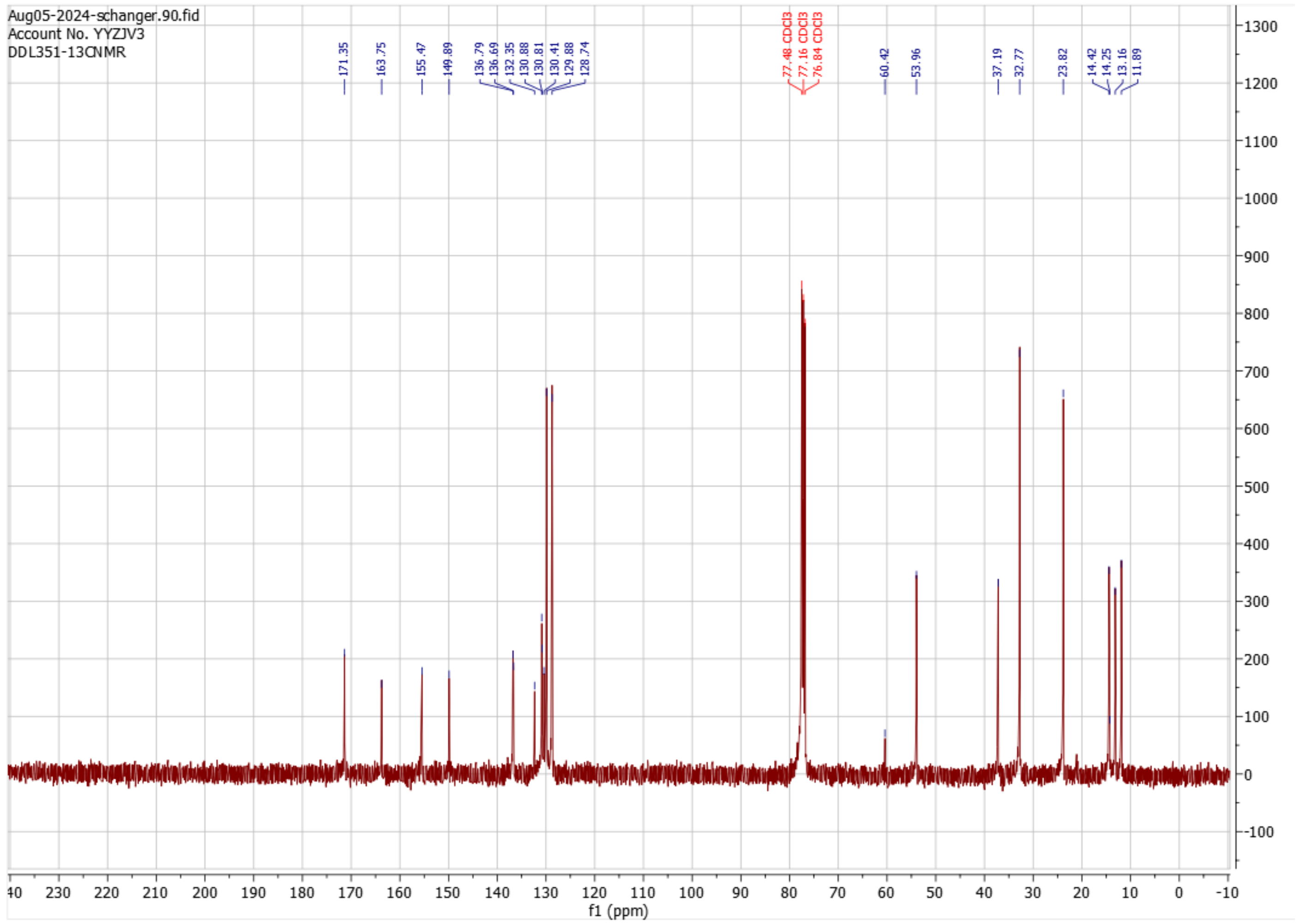

^{13}C NMR ($CDCl_3$) δ 171.35, 163.75, 155.47, 149.89, 136.79, 136.69, 132.,35, 130.88, 130.81, 130.41, 129.88, 128.74, 60.42, 53.96, 37.19, 32.77, 23.82, 14.42, 14.25, 13.16, 11.89.

DDL-352: *(S)-2-(4-(4-chlorophenyl)-2,3,9-trimethyl-6H-thieno[3,2-f][1,2,4]triazolo[4,3-a][1,4]diazepin-6-yl)-1-(pyrrolidin-1-yl)ethan-1-one* ^{1}*H NMR*

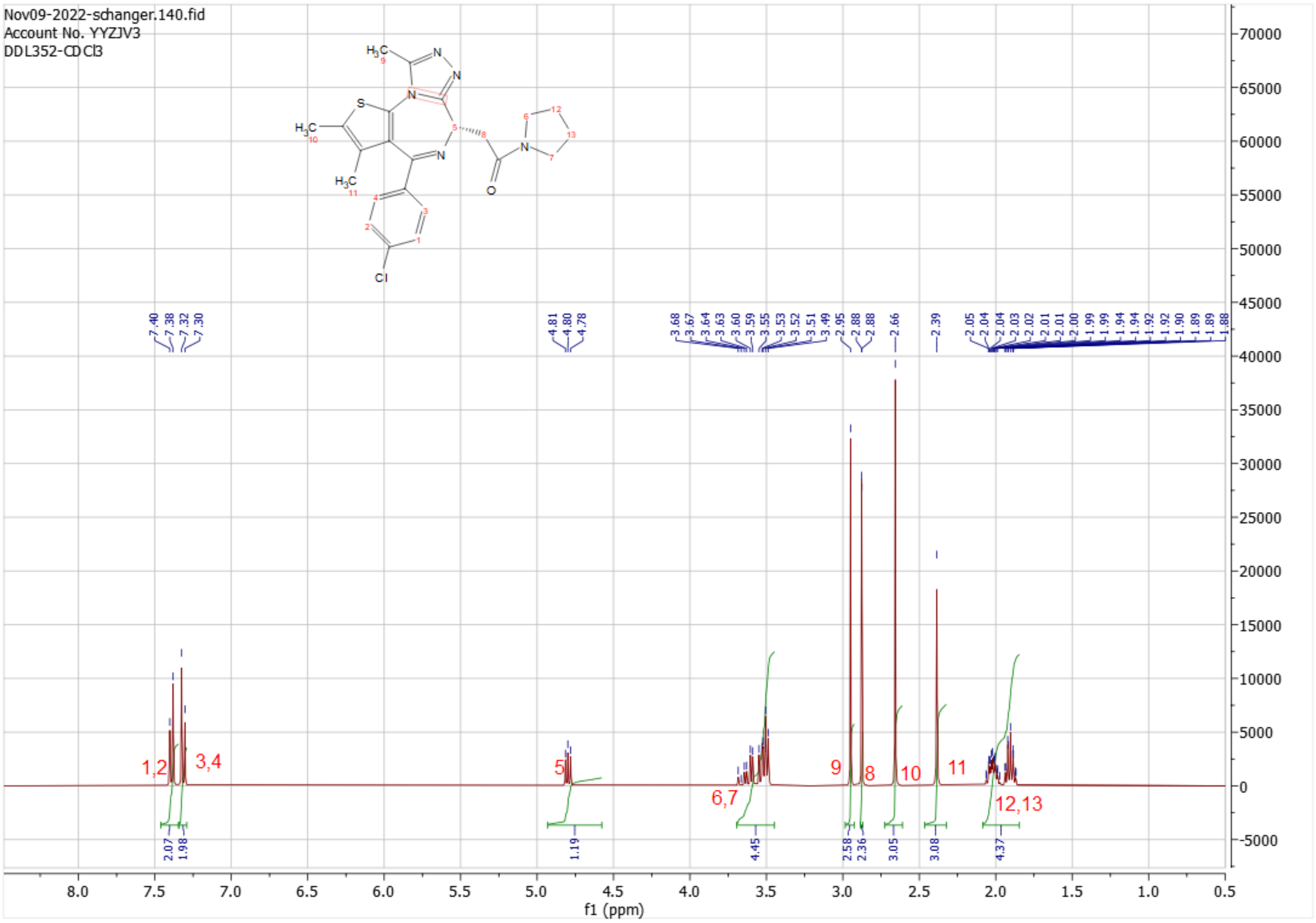

^{1}H NMR (400 MHz, $CDCl_3$) δ 7.39 (d, *J* = 8 Hz, 2H), 7.31 (d, *J* = 8 Hz, 2H), 4.80 (t, *J* = 4 Hz 1H), 3.69 – 3.49 (m, 4H), 2.95 (s, 3H), 2.88 (d, *J* = 0.6 Hz, 2H), 2.66 (s, 3H), 2.39 (s, 3H), 2.09 – 1.85 (m, 4H).

DDL-352: *(S)-2-(4-(4-chlorophenyl)-2,3,9-trimethyl-6H-thieno[3,2-f][1,2,4]triazolo[4,3-a][1,4]diazepin-6-yl)-1-(pyrrolidin-1-yl)ethan-1-one ^{13}C NMR*

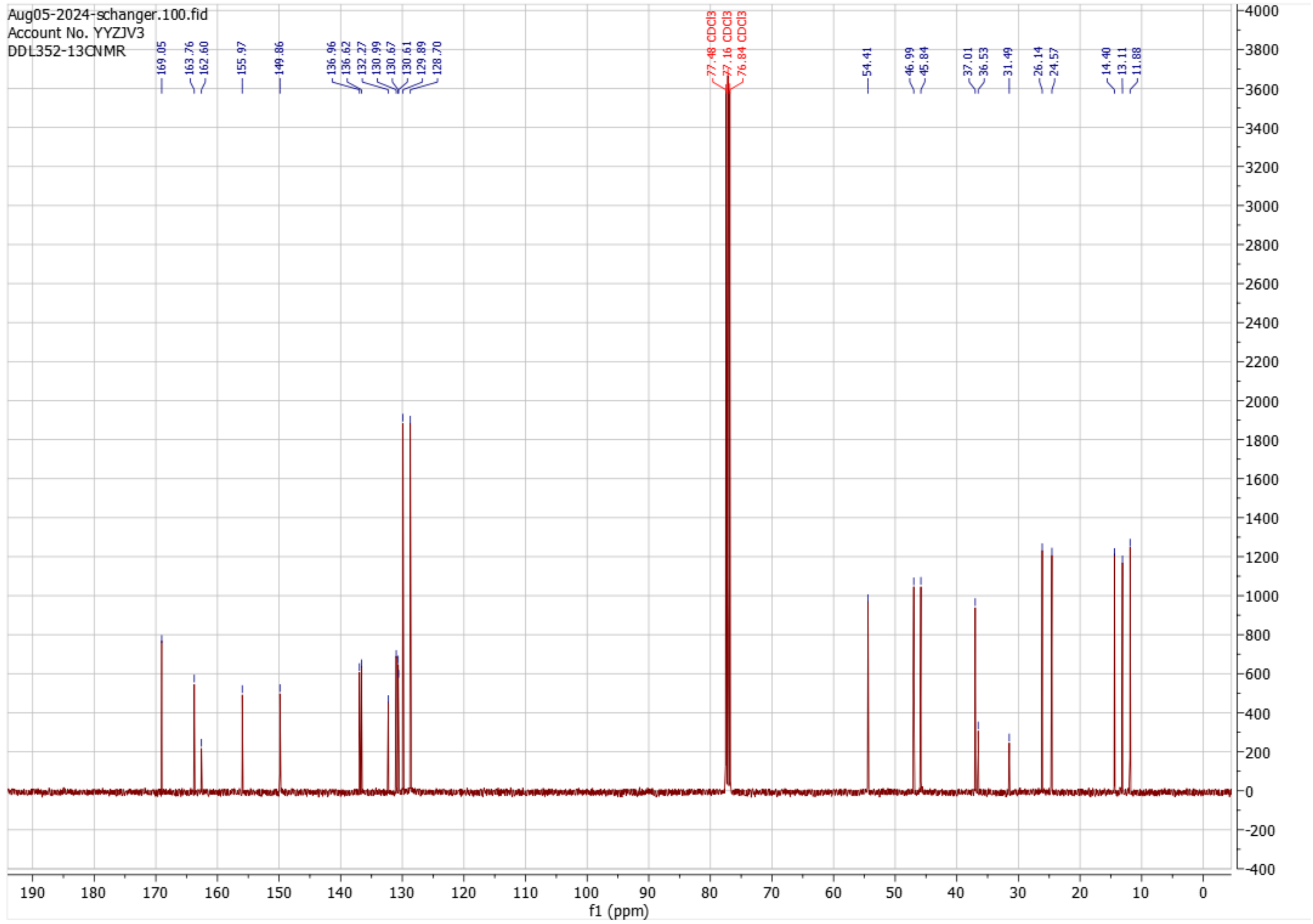

^{13}C NMR ($CDCl_3$) δ 169.05, 163.76, 162.60, 155.97, 149.86, 136.96, 136.62, 132.27, 130.99, 130.67, 130.61, 129.89, 128.70, 54.41, 46.99, 45.84, 37.01, 36.53, 31.49, 26.14, 24.57, 14.40, 13.11, 11.88.

DDL-353: *(S)-2-(4-(4-chlorophenyl)-2,3,9-trimethyl-6H-thieno[3,2-f][1,2,4]triazolo[4,3-a][1,4]diazepin-6-yl)-N-cyclopentylacetamide* ^{1}*H NMR*

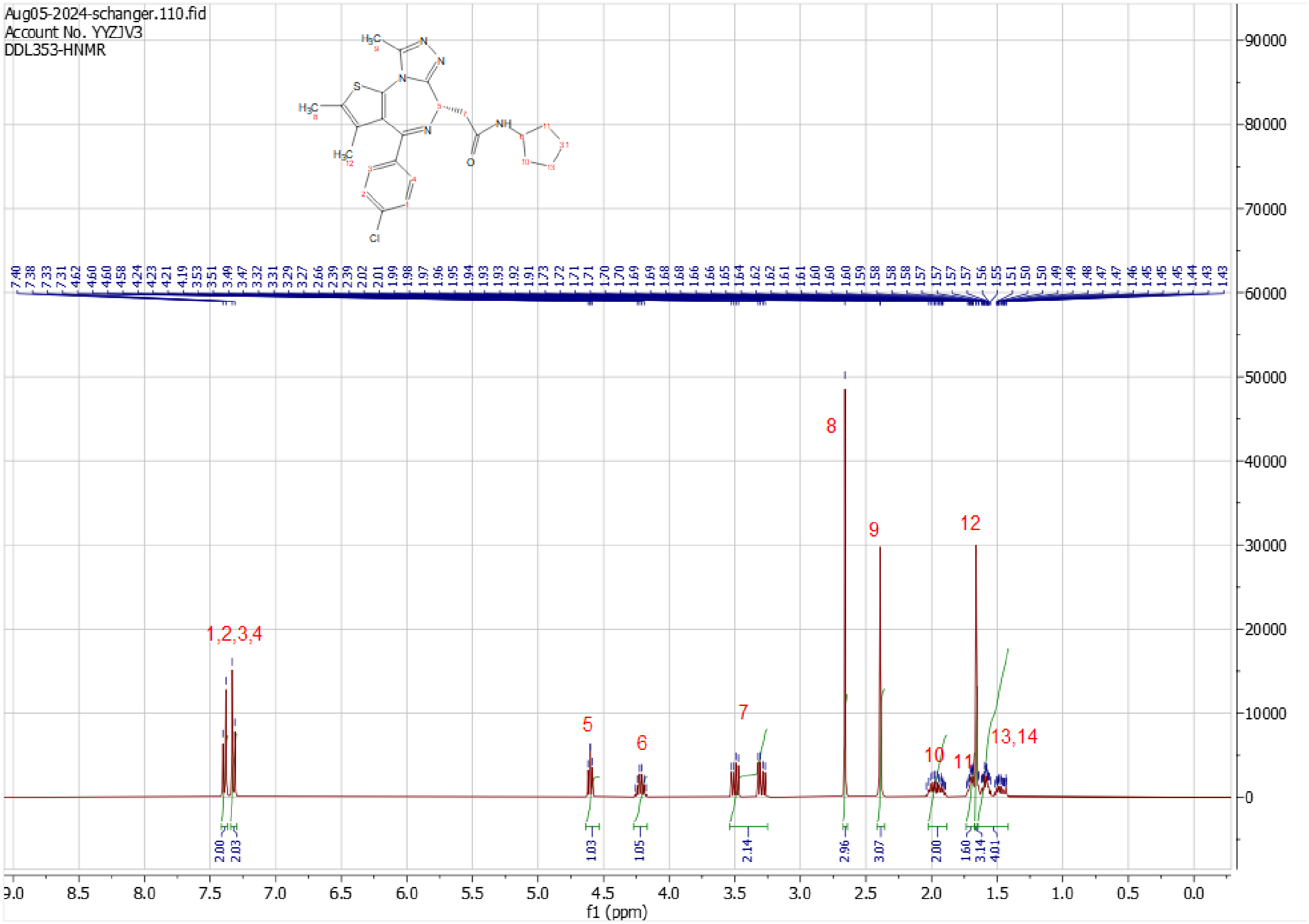

^{1}H NMR (400 MHz, $CDCl_3$) δ 7.39 (d, *J* = 8.6 Hz, 2H), 7.32 (d, *J* = 8.7 Hz, 2H), 4.60 (t, *J* = 8 Hz 1H), 4.26-4.18 (m, 1H), 3.53-3.27 (m, 2H), 2.66 (s, 3H), 2.39 (s, 3H), 2.04-1.89 (m, 2H), 1.73-1.68 (m, 2H), 1.66 (s, 1H), 1.65-1.43 (m, 4H).

DDL-353: *(S)-2-(4-(4-chlorophenyl)-2,3,9-trimethyl-6H-thieno[3,2-f][1,2,4]triazolo[4,3-a][1,4]diazepin-6-yl)-N-cyclopentylacetamide* ^{13}*C NMR*

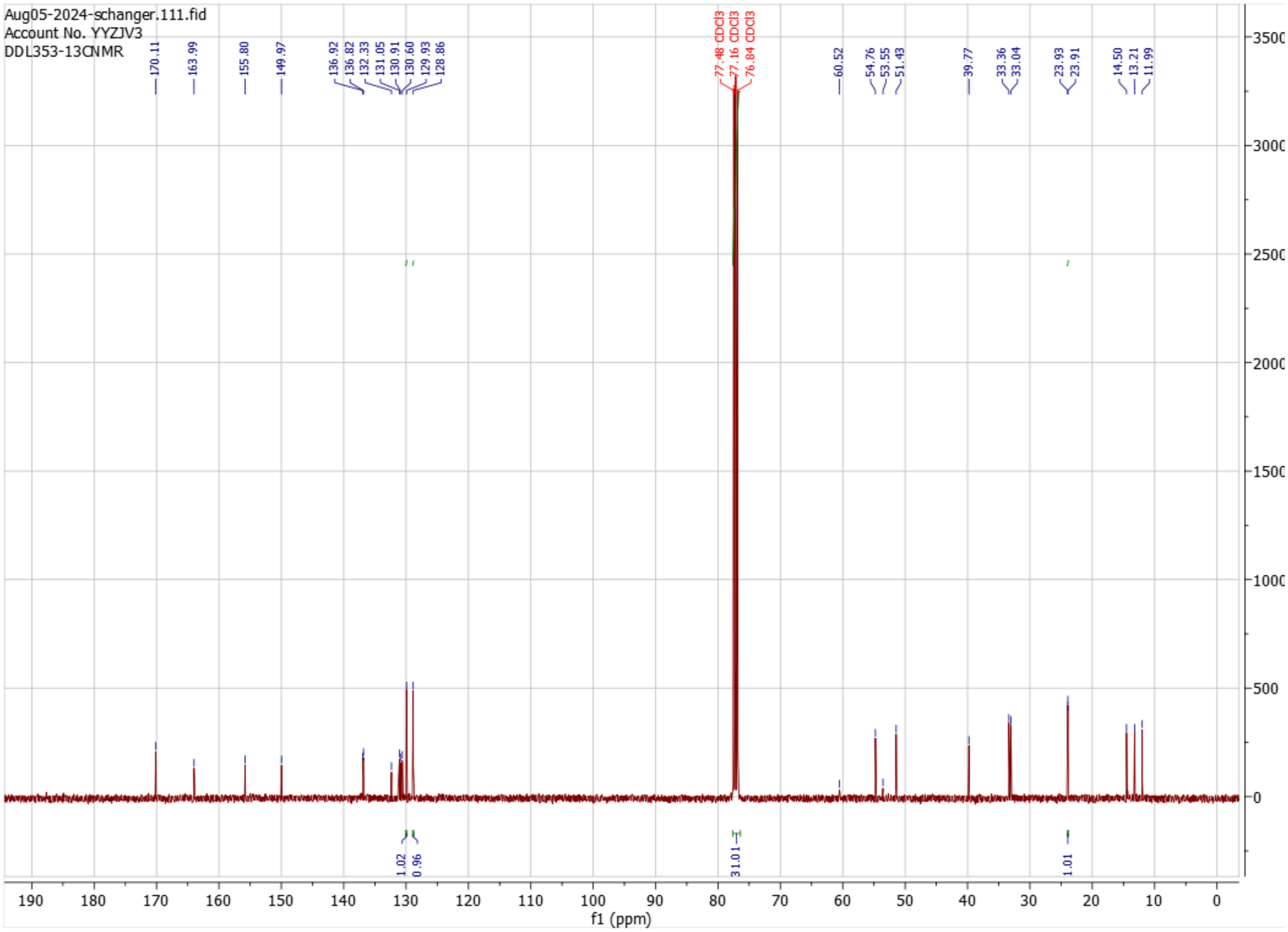

^{13}C NMR ($CDCl_3$) δ 170.11, 163.99, 155.80, 149.97, 136.92, 136.82, 132.33, 131.05, 130.91, 130.60, 129.93, 128.86, 60.52, 54.76, 53.55, 51.43, 39.77, 33.36, 33.04, 23.93, 23.91, 14.50, 13.21, 11.99.

DDL-354: *Isopropyl (S)-(2-(4-(4-chlorophenyl)-2,3,9-trimethyl-6H-thieno[3,2-f][1,2,4]triazolo[4,3-a][1,4]diazepin-6-yl)acetyl)glycinate* ^{1}*H NMR*

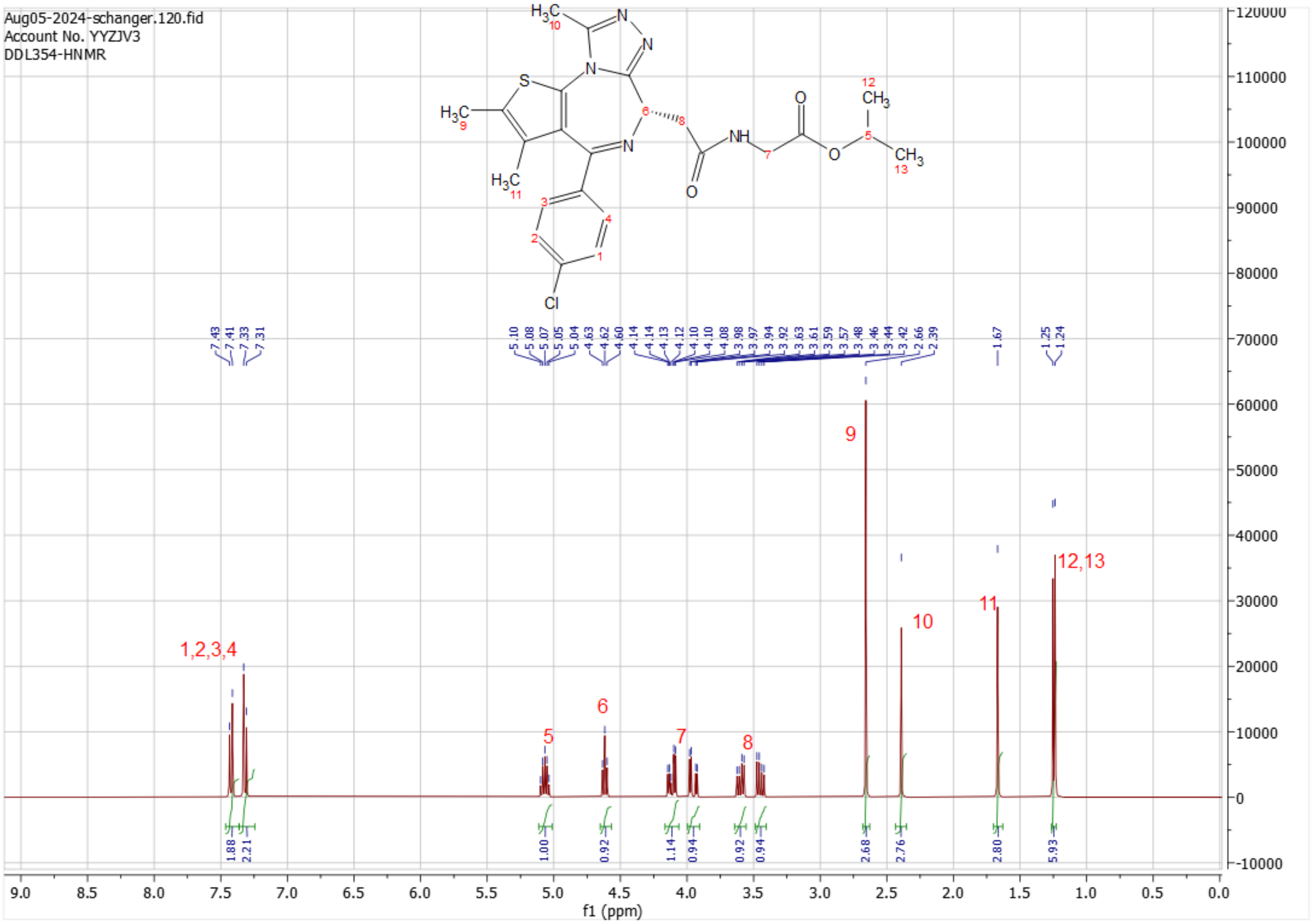

^{1}H NMR (400 MHz, $CDCl_3$) δ 7.42 (d, *J* = 8.6 Hz, 2H), 7.32 (d, *J* = 8.7 Hz, 2H), 5.06 (hept, *J* = 6.3 Hz, 1H), 4.62 (t, *J* = 6.9 Hz, 1H), 4.16 – 4.06 (m, 1H), 3.95 (dd, *J* = 18.2, 4.9 Hz, 1H), 3.60 (dd, *J* = 14.6, 6.8 Hz, 1H), 3.45 (dd, *J* = 14.6, 7.1 Hz, 1H), 2.66 (s, 3H), 2.39 (s, 3H), 1.67 (s, 3H), 1.24 (d, *J* = 6.3 Hz, 6H).

DDL-354: *Isopropyl (S)-(2-(4-(4-chlorophenyl)-2,3,9-trimethyl-6H-thieno[3,2-f][1,2,4]triazolo[4,3-a][1,4]diazepin-6-yl)acetyl)glycinate ^{13}C NMR*

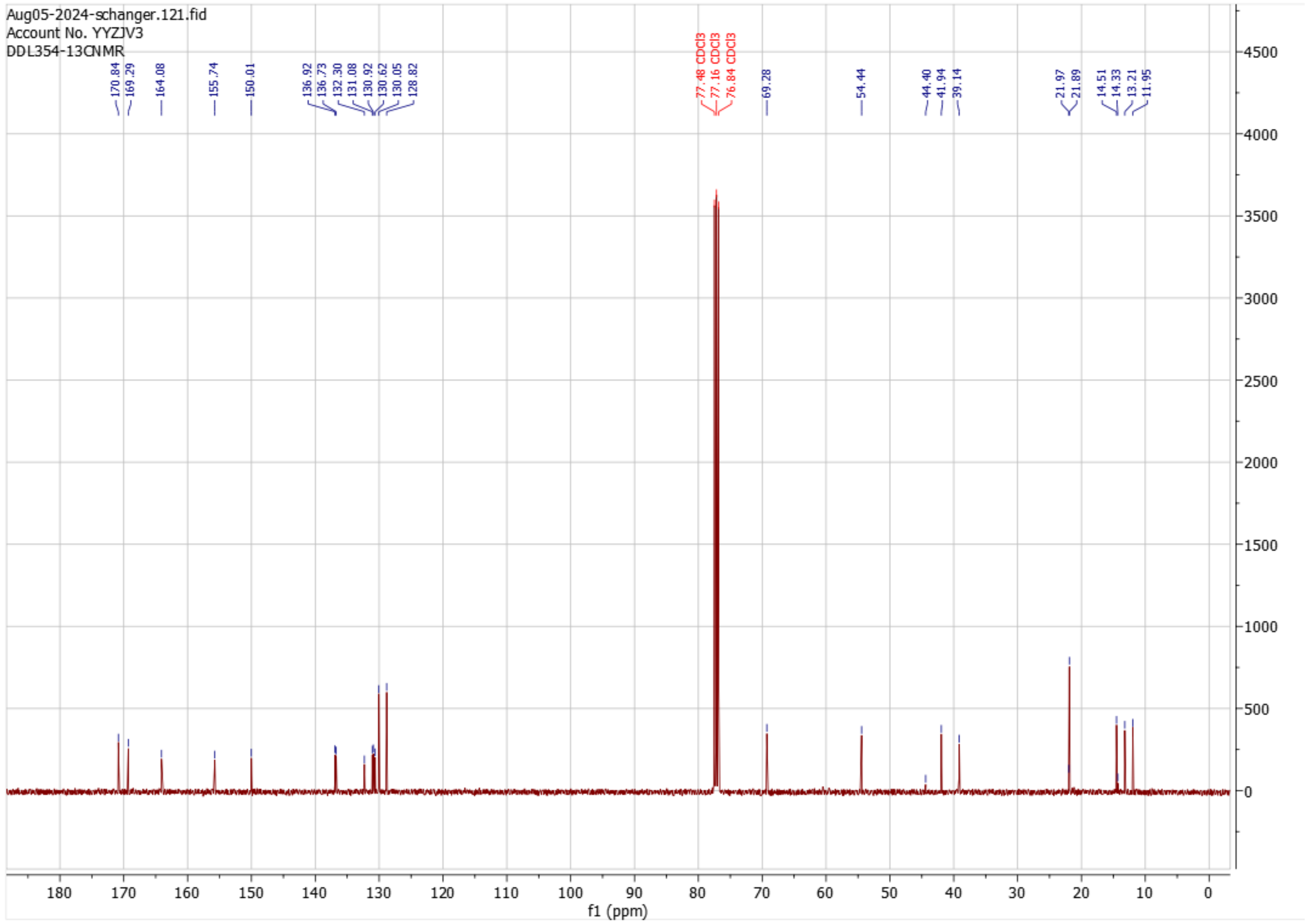

^{13}C NMR ($CDCl_3$) δ 170.84, 169.29, 164.08, 155.74, 136.92, 136.73, 132.30, 131.08, 130.92, 130.62, 130.05, 128.82, 69.28, 54.44, 44.40, 41.94, 39.14, 21.97, 21.89, 14.51, 13.21, 11.95.

DDL-355: *Cyclobutyl (S)-2-(4-(4-chlorophenyl)-2,3,9-trimethyl-6H-thieno[3,2-f][1,2,4]triazolo[4,3-a][1,4]diazepin-6-yl)acetate* ^{1}*H NMR*

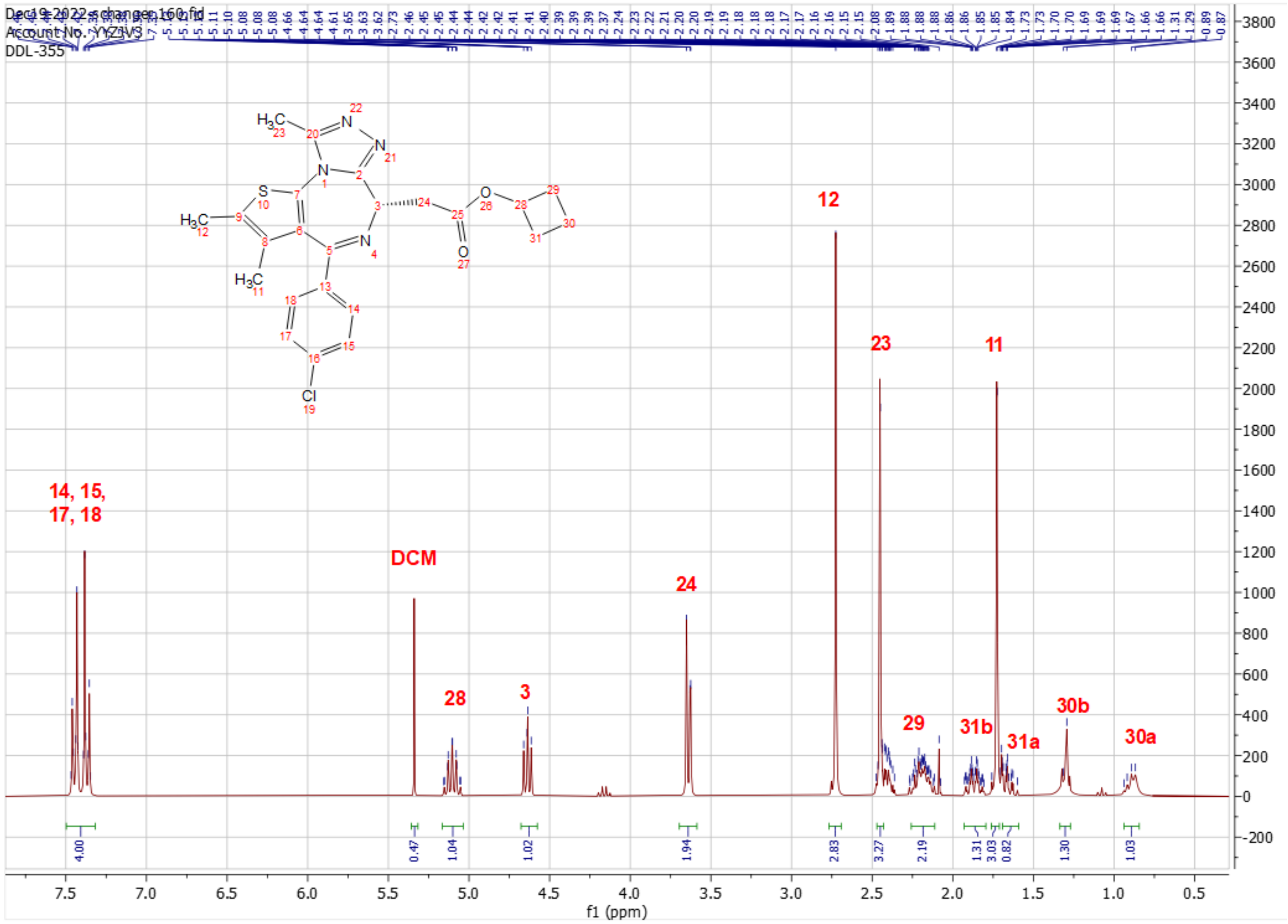

^{1}H NMR (300 MHz, $CDCl_3$) δ 7.50 – 7.32 (m, 4H), 5.16 – 5.04 (m, 1H), 4.64 (dd, J = 7.5, 6.6 Hz, 1H), 3.70 – 3.59 (m, 2H), 2.73 (s, 3H), 2.45 (s, 3H), 2.26 – 2.11 (m, 2H), 1.93 – 1.79 (m, 1H), 1.73 (s, 3H), 1.69 – 1.59 (m, 1H), 1.30 (dt, J = 7.1, 4.1 Hz, 1H), 0.90 (dd, J = 14.6, 6.8 Hz, 1H).

DDL-355: *Cyclobutyl (S)-2-(4-(4-chlorophenyl)-2,3,9-trimethyl-6H-thieno[3,2-f][1,2,4]triazolo[4,3-a][1,4]diazepin-6-yl)acetate* ^{13}*C NMR*

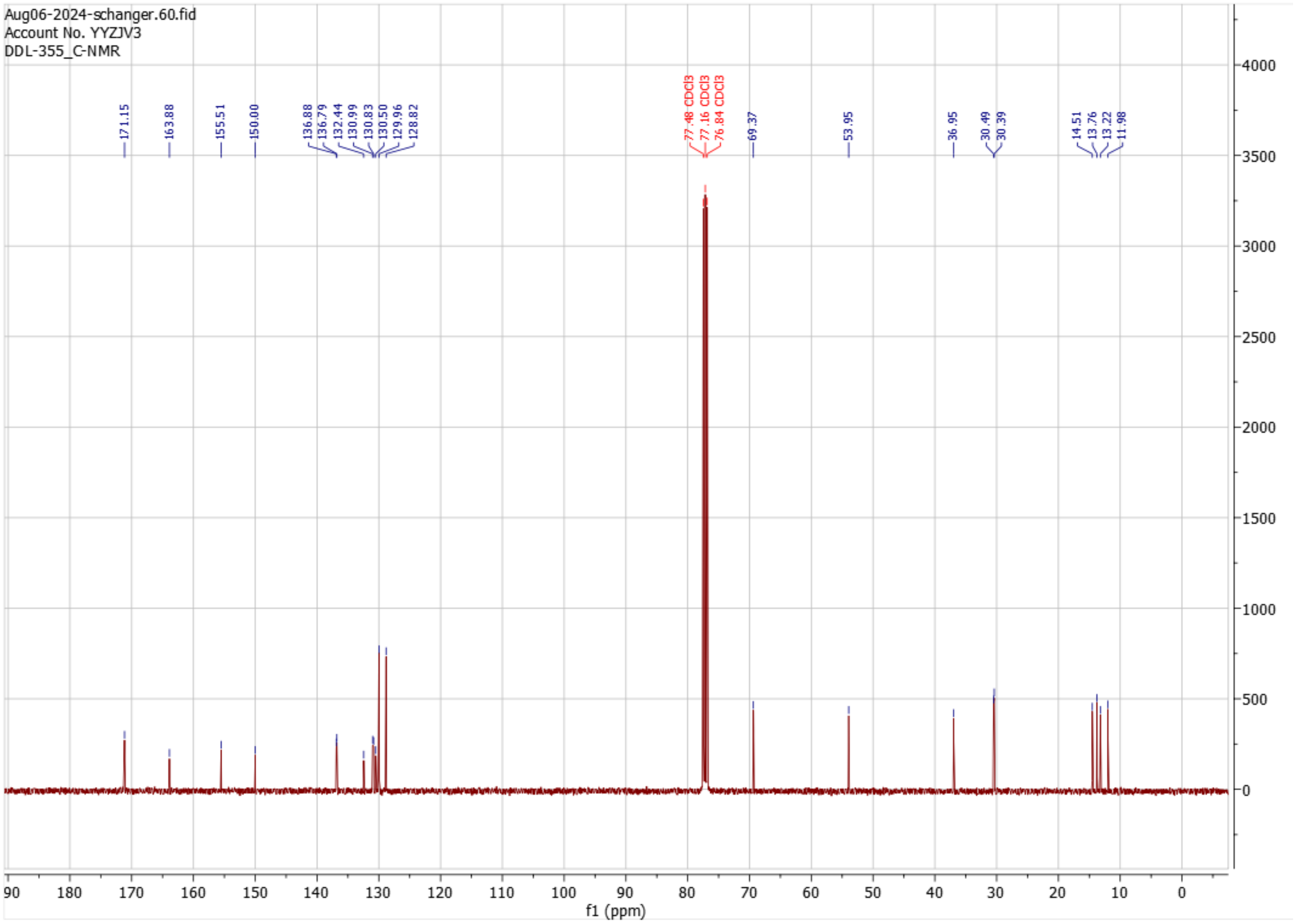

^{13}C NMR ($CDCl_3$) δ 171.15, 163.88, 155.51, 150.00, 136.88, 136.79, 132.44, 130.99, 130.83, 130.50, 129.96, 128.82, 69.37, 53.95, 36.95, 30.49, 30.39, 14.51, 13.76, 13.22, 11.98.

DDL-356: *Oxetan-3-yl (S)-2-(4-(4-chlorophenyl)-2,3,9-trimethyl-6H-thieno[3,2-f][1,2,4]triazolo[4,3-a][1,4]diazepin-6-yl)acetate* ^{1}*H NMR*

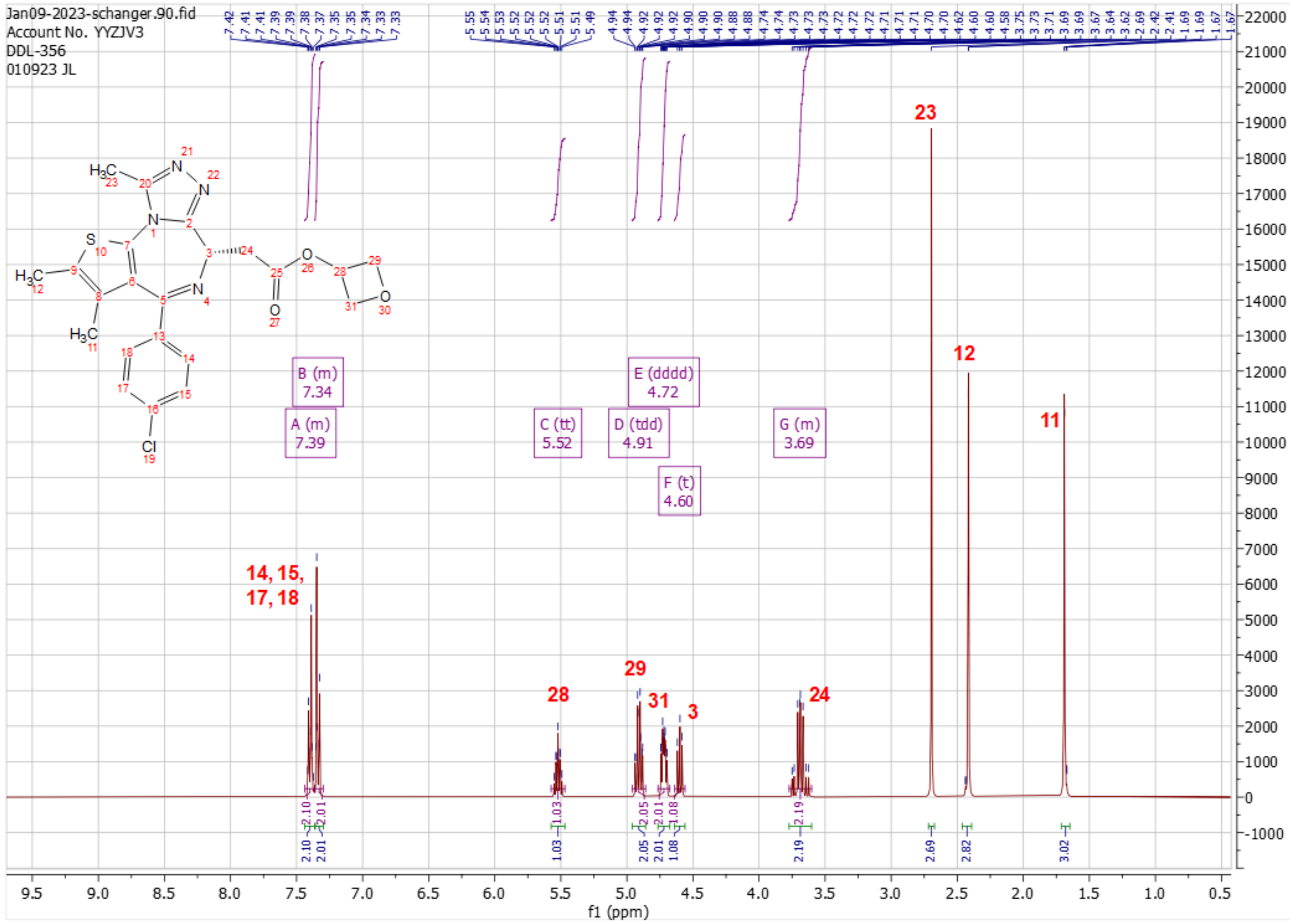

^{1}H NMR (400 MHz, $CDCl_3$) δ 7.44 – 7.36 (m, 2H), 7.36 – 7.30 (m, 2H), 5.52 (tt, J = 6.4, 5.3 Hz, 1H), 4.91 (tdd, J = 7.5, 6.3, 1.0 Hz, 2H), 4.72 (dddd, J = 7.5, 5.0, 3.8, 0.9 Hz, 2H), 4.60 (t, J = 6.3 Hz, 1H), 3.77 – 3.60 (m, 2H), 2.72-2.67 (s, 3H), 2.46-2.39 (s, 3H), 1.71-1.65 (s, 3H).

DDL-356: *Oxetan-3-yl (S)-2-(4-(4-chlorophenyl)-2,3,9-trimethyl-6H-thieno[3,2-f][1,2,4]triazolo[4,3-a][1,4]diazepin-6-yl)acetate* ^{13}C *NMR*

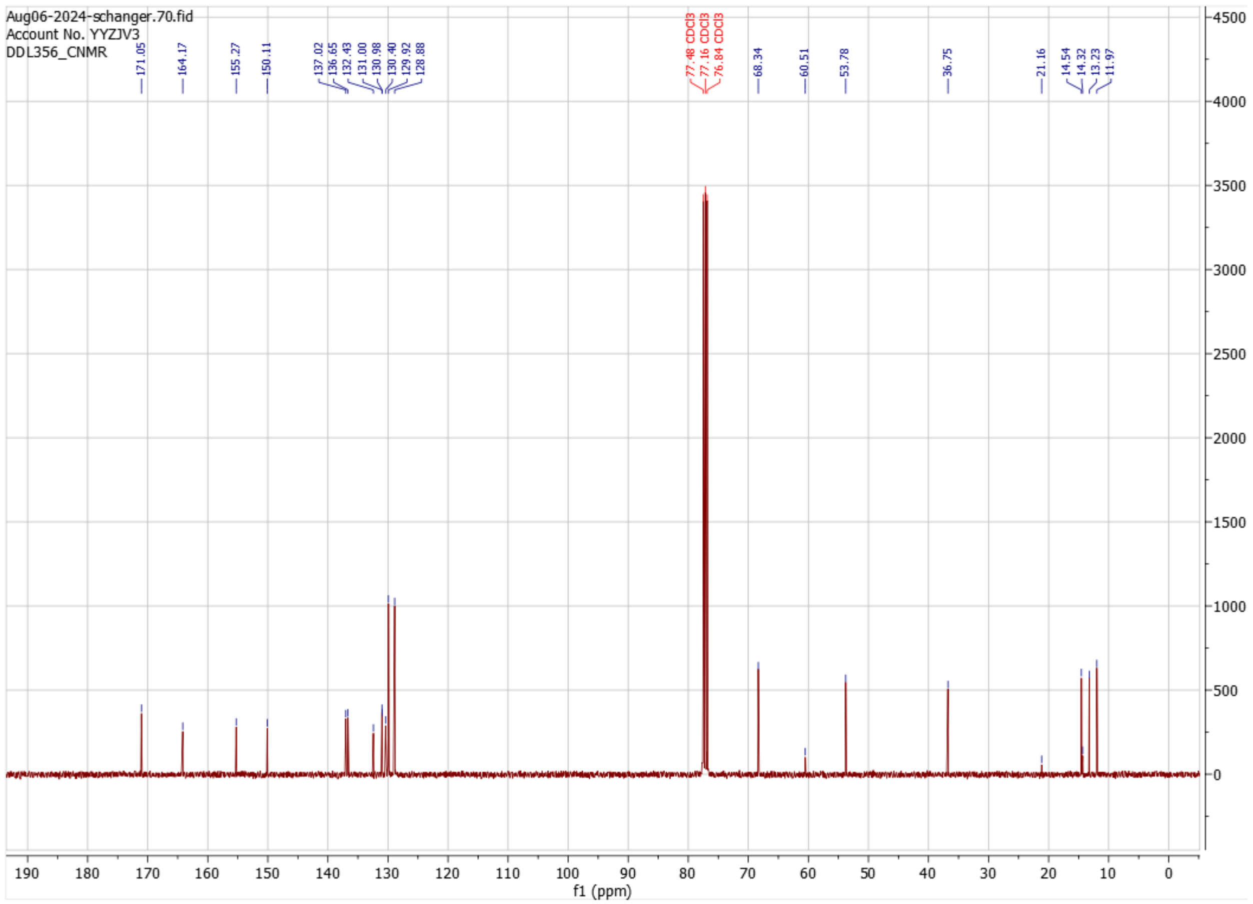

^{13}C NMR ($CDCl_3$) δ 171.05, 164.17, 155.27, 150.11, 137.02, 136.65, 132.43, 131.00, 130.98, 130.40, 129.92, 128.88, 68.34, 60.51, 53.78, 36.75, 21.16, 14.54, 13.23, 11.97.

DDL-357: *Cyclopropyl (S)-2-(4-(4-chlorophenyl)-2,3,9-trimethyl-6H-thieno[3,2-f][1,2,4]triazolo[4,3-a][1,4]diazepin-6-yl)acetate* ^{1}*H NMR*

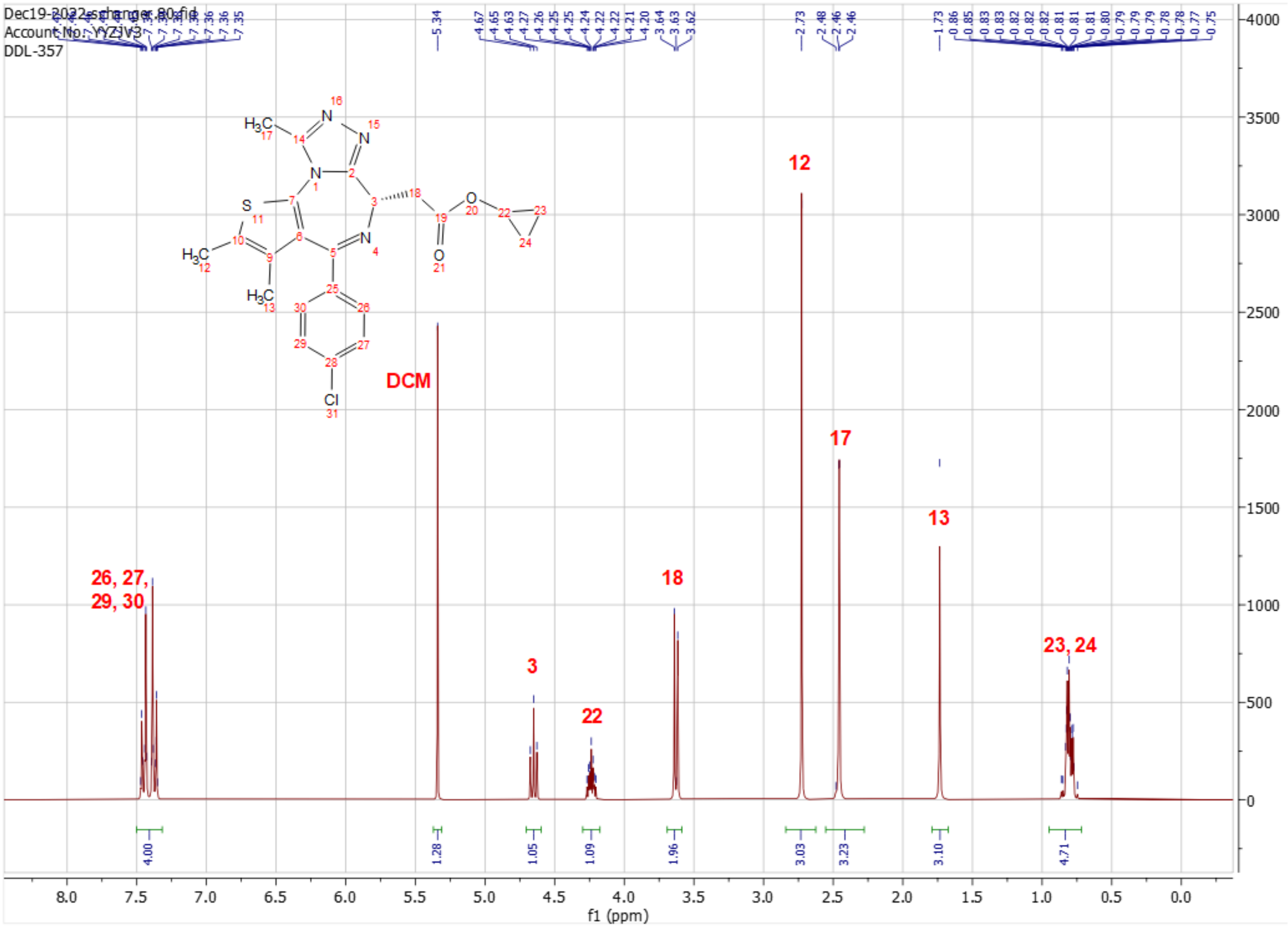

δ ^{1}H NMR (400 MHz, CDCl3) δ^{1}H NMR (300 MHz, $CDCl_3$) δ 7.50 – 7.32 (m, 4H), 4.65 (t, *J* = 7.0 Hz, 1H), 4.30 – 4.17 (m, 1H), 3.63 (d, *J* = 7.1 Hz, 2H), 2.73 (s, 3H), 2.46 (d, *J* = 0.8 Hz, 3H), 1.73 (s, 3H), 0.95 – 0.72 (m, 4H).

DDL-357: *Cyclopropyl (S)-2-(4-(4-chlorophenyl)-2,3,9-trimethyl-6H-thieno[3,2-f][1,2,4]triazolo[4,3-a][1,4]diazepin-6-yl)acetate ^{13}C NMR*

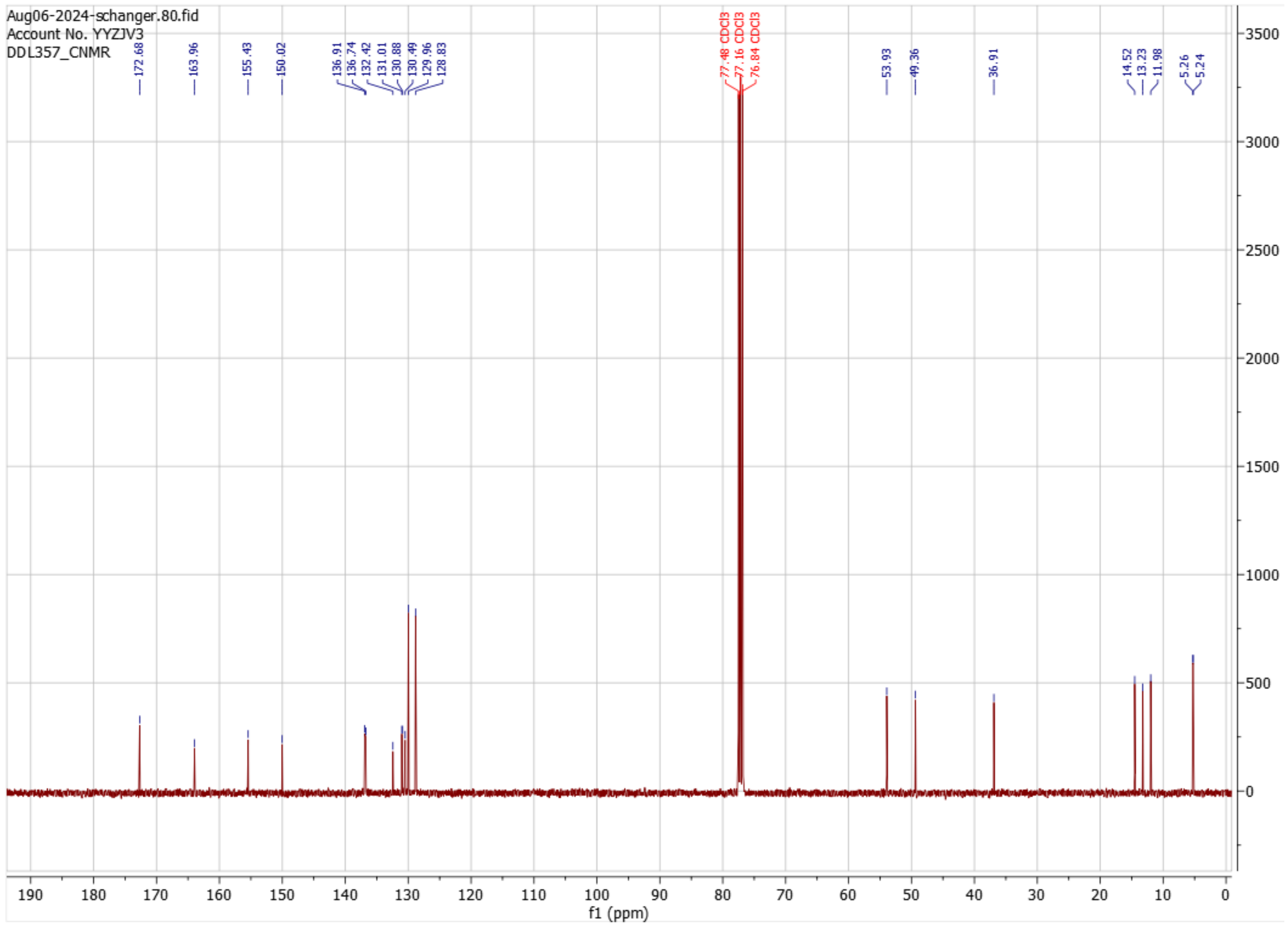

^{13}C NMR ($CDCl_3$) δ 172.68, 163.96, 155.43, 150.02, 136.91, 136.74, 132.42, 131.01, 130.88, 130.49, 129.96, 128.83, 53.93, 49.36, 36.91, 14.52, 13.23,11.98, 5.25.

DDL-358: *Butyl (S)-2-(4-(4-chlorophenyl)-2,3,9-trimethyl-6H-thieno[3,2-f][1,2,4]triazolo[4,3-a][1,4]diazepin-6-yl)acetate* ^{1}*H NMR*

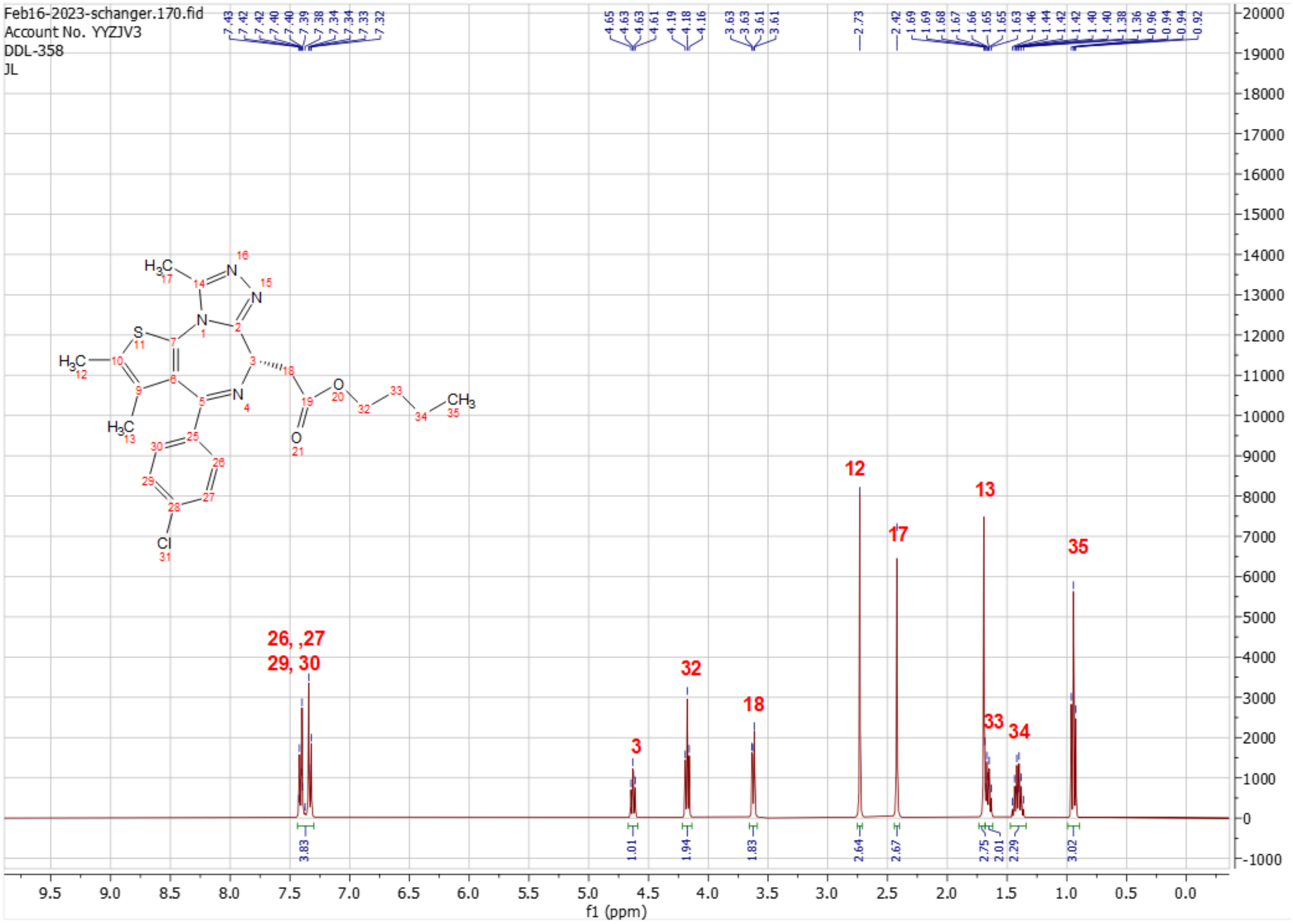

δ ^{1}H NMR (400 MHz, CDCl3) δ 7.44 – 7.30 (m, 4H), 4.63 (t, J = 6.7 Hz, 1H), 4.18 (t, J = 6.6 Hz, 2H), 3.62 (d, J = 1.4 Hz, 2H), 2.73 (s, 3H), 2.42 (s, 3H), 1.69 (s, 3H), 1.68 – 1.62 (m, 2H), 1.47 – 1.34 (m, 2H), 0.94 (t, J = 7.4 Hz, 3H).

DDL-358: *Butyl (S)-2-(4-(4-chlorophenyl)-2,3,9-trimethyl-6H-thieno[3,2-f][1,2,4]triazolo[4,3-a][1,4]diazepin-6-yl)acetate ^{13}C NMR*

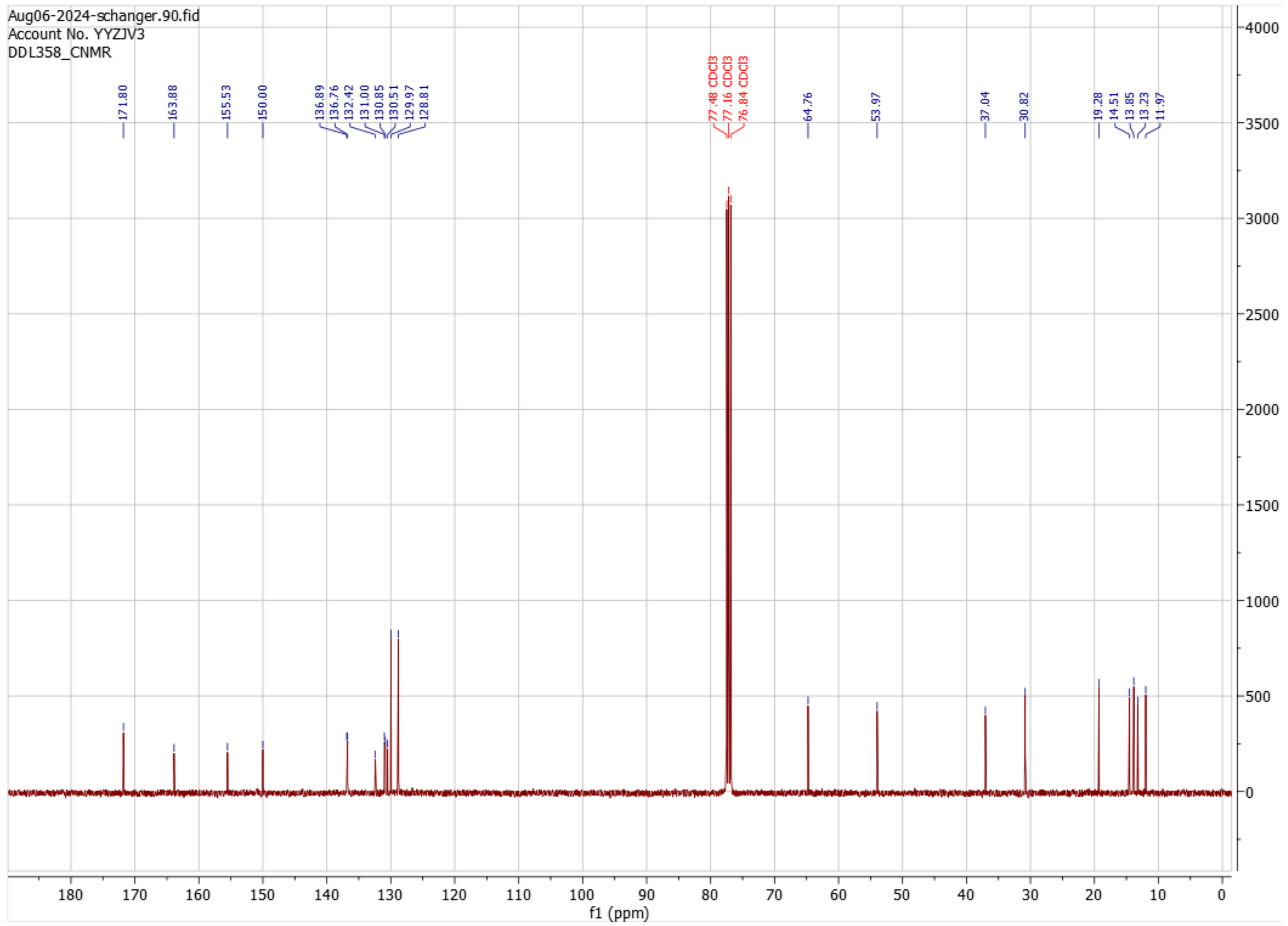

^{13}C NMR ($CDCl_3$) δ 171.80, 163.88, 155.53, 150.00, 136.89, 136.76, 132.42, 131.00, 130.85, 130.51, 129.97, 128.81, 64.76, 53.97, 37.04, 30.82, 19.28, 14.51, 13.85, 13.23, 11.97.

DDL-359: *Isopropyl (2-((S)-4-(4-chlorophenyl)-2,3,9-trimethyl-6H-thieno[3,2-f][1,2,4]triazolo[4,3-a][1,4]diazepin-6-yl)acetyl)-L-alaninate* ^{1}*H NMR*

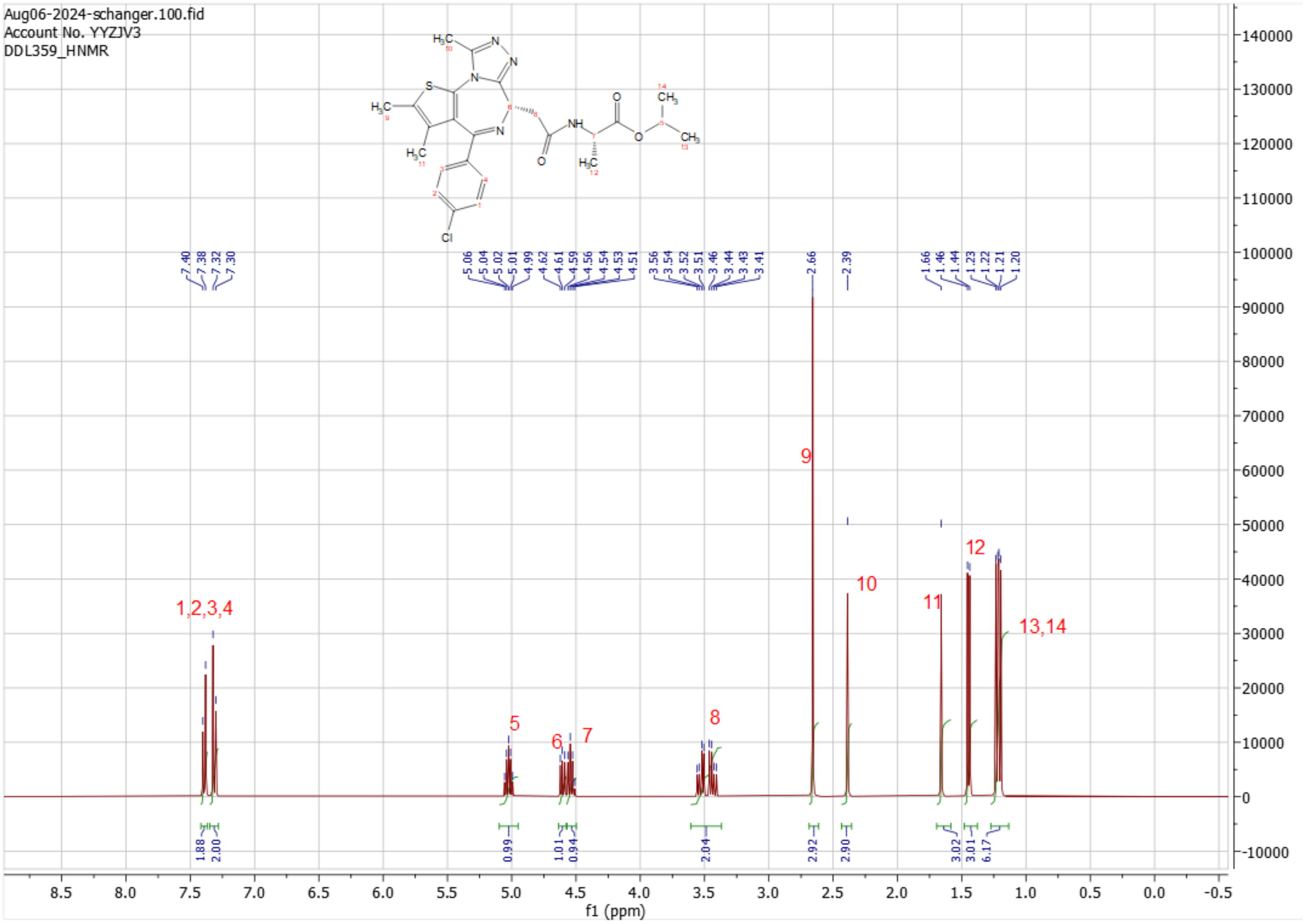

^{1}H NMR (400 MHz, $CDCl_3$) δ 7.39 (d, J = 8.6 Hz, 2H), 7.31 (d, J = 8.8 Hz, 2H), 5.02 (hept, J = 6.3 Hz, 1H), 4.60 (t, J = 6.7 Hz, 1H), 4.54 (q, J = 7.2 Hz, 1H), 3.61 – 3.37 (m, 2H), 2.66 (s, 3H), 2.39 (s, 3H), 1.66 (s, 3H), 1.45 (d, J = 7.2 Hz, 3H), 1.21 (dd, J = 9.5, 6.2 Hz, 6H).

DDL-359: *Isopropyl (2-((S)-4-(4-chlorophenyl)-2,3,9-trimethyl-6H-thieno[3,2-f][1,2,4]triazolo[4,3-a][1,4]diazepin-6-yl)acetyl)-L-alaninate* ^{13}*C NMR*

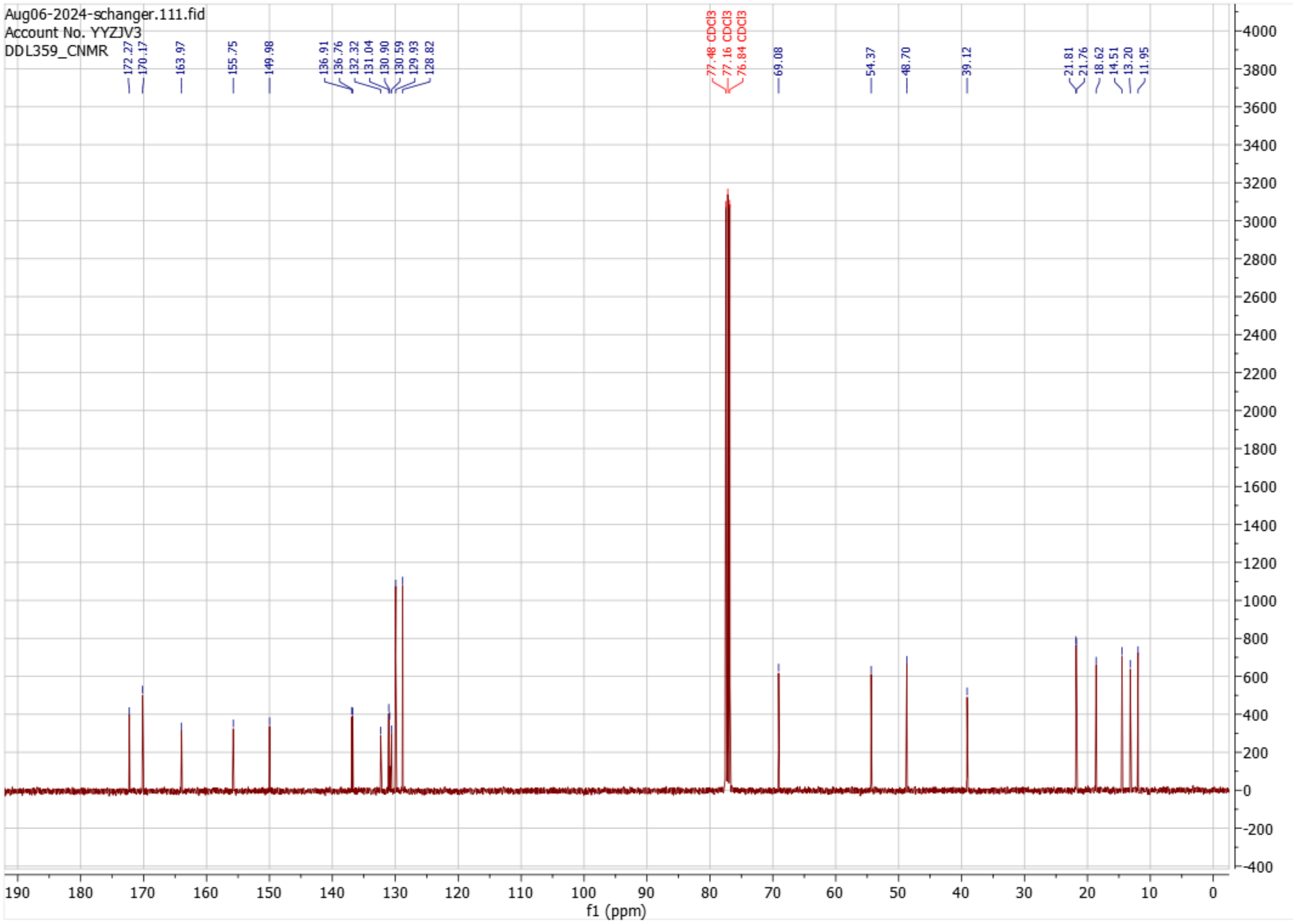

^{13}C NMR ($CDCl_3$) δ 172.27, 170.17, 163.97, 155.75, 149.98, 136.91, 136.76, 132.32, 131.04, 130.90, 130.59, 129.93, 128.82, 69.08, 54.37, 48.70, 39.12, 21.81, 21.76, 18.62, 14.51, 13.20, 11.95.

DDL-360: *(S)-2-(4-(4-chlorophenyl)-2,3,9-trimethyl-6H-thieno[3,2-f][1,2,4]triazolo[4,3-a][1,4]diazepin-6-yl)-N-hydroxyacetamide* ^{1}*H NMR*

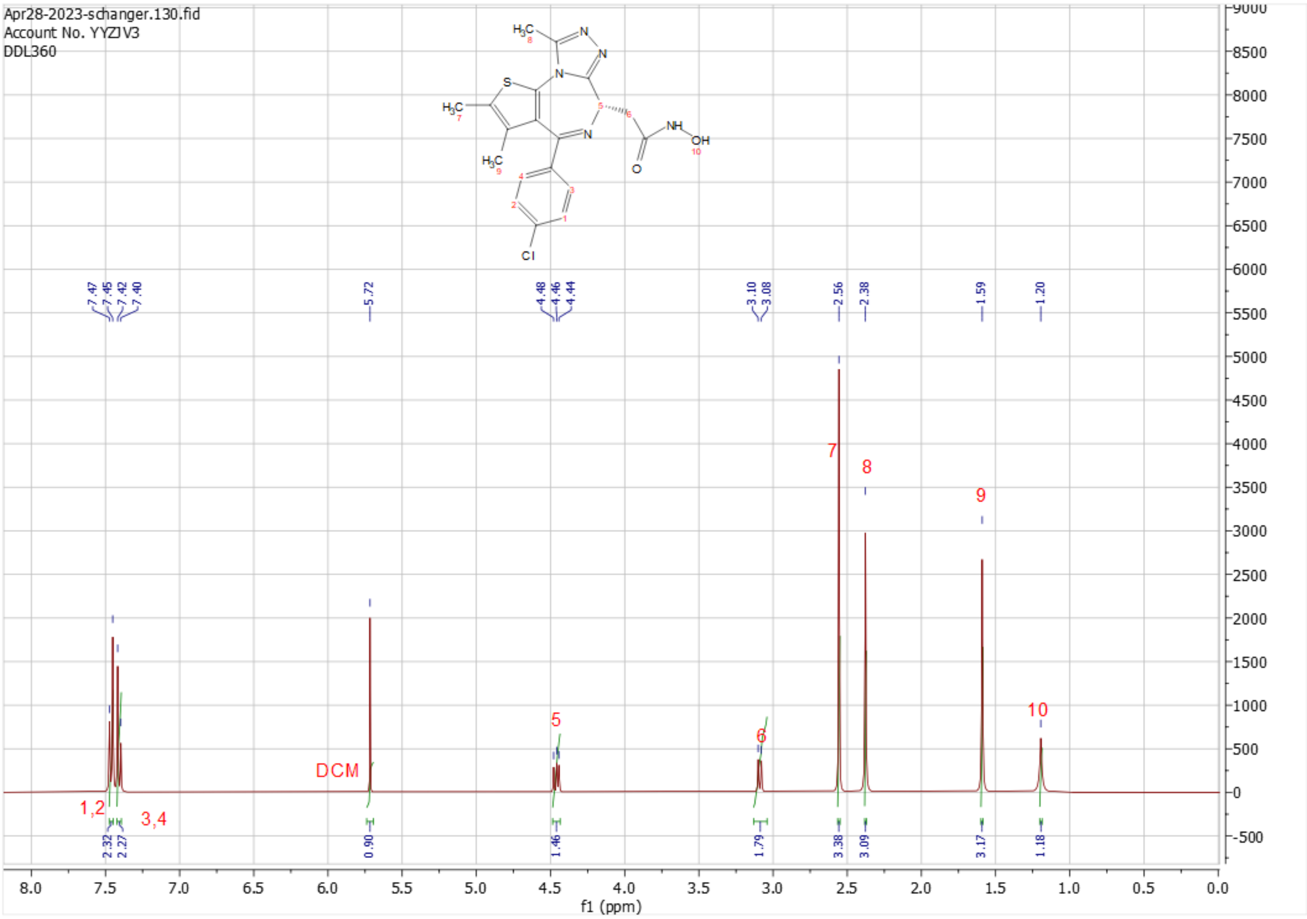

^{1}H NMR (400 MHz, $CDCl_3$) δ 7.46 (d, J = 8 Hz, 2H), 7.41 (d, J = 8 Hz, 2H), 4.46 (t, J = 8 Hz, 1H), 3.09 (d, J = 8 Hz), 2.56 (s, 3H), 2.38 (s, 3H), 1.59 (s, 3H), 1.20 (s, 1H).

DDL-360: *(S)-2-(4-(4-chlorophenyl)-2,3,9-trimethyl-6H-thieno[3,2-f][1,2,4]triazolo[4,3-a][1,4]diazepin-6-yl)-N-hydroxyacetamide ^{13}C NMR*

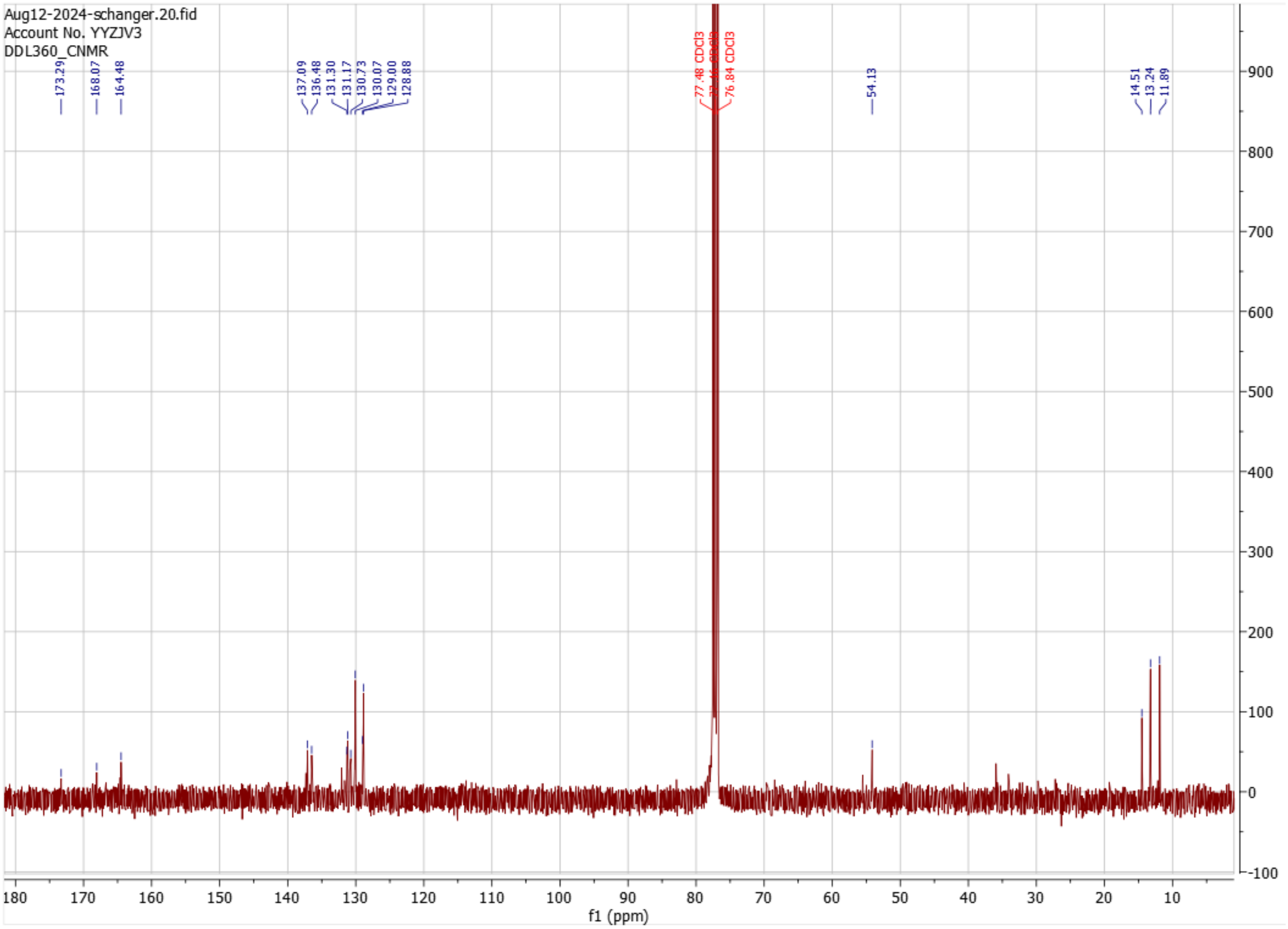

^{13}C NMR ($CDCl_3$) δ 173.29, 168.07, 164.48, 137.09, 136.48, 131.30, 131.17, 130.73, 130.07, 129.00, 128.88, 54.13, 14.51, 13.24, 11.89

High resolution MS for each compound

DDL351:

Cyclopentyl(S)-2-(4-(4-chlorophenyl)-2,3,9-trimethyl-6H-thieno[3,2-f][1,2,4]triazolo[4,3-a][1,4]diazepin-6-yl)acetate

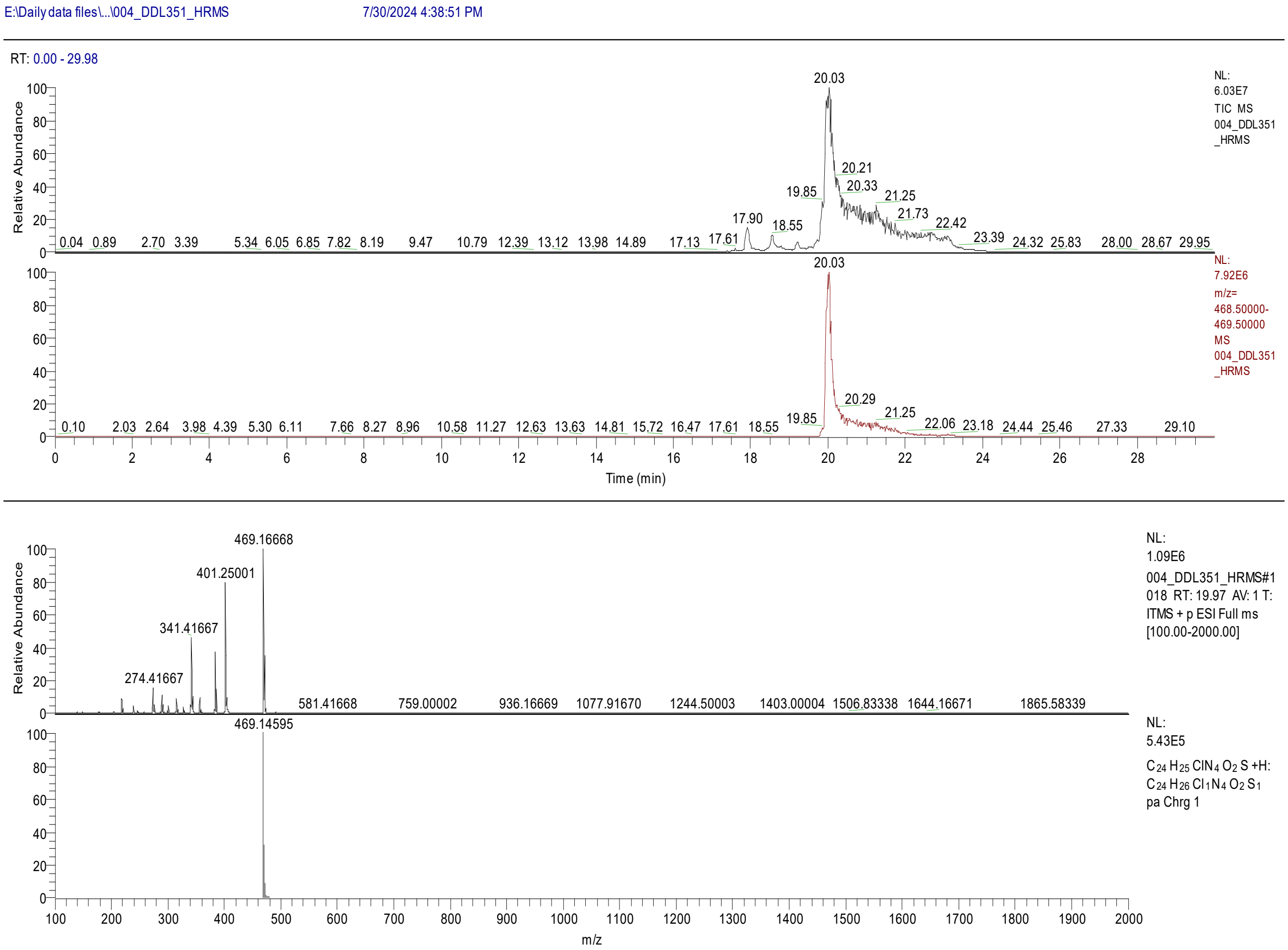

DDL 352:

(S)-2-(4-(4-chlorophenyl)-2,3,9-trimethyl-6H-thieno[3,2-f][1,2,4]triazolo[4,3-a][1,4]diazepin-6-yl)-1-(pyrrolidin-1-yl)ethan-1-one

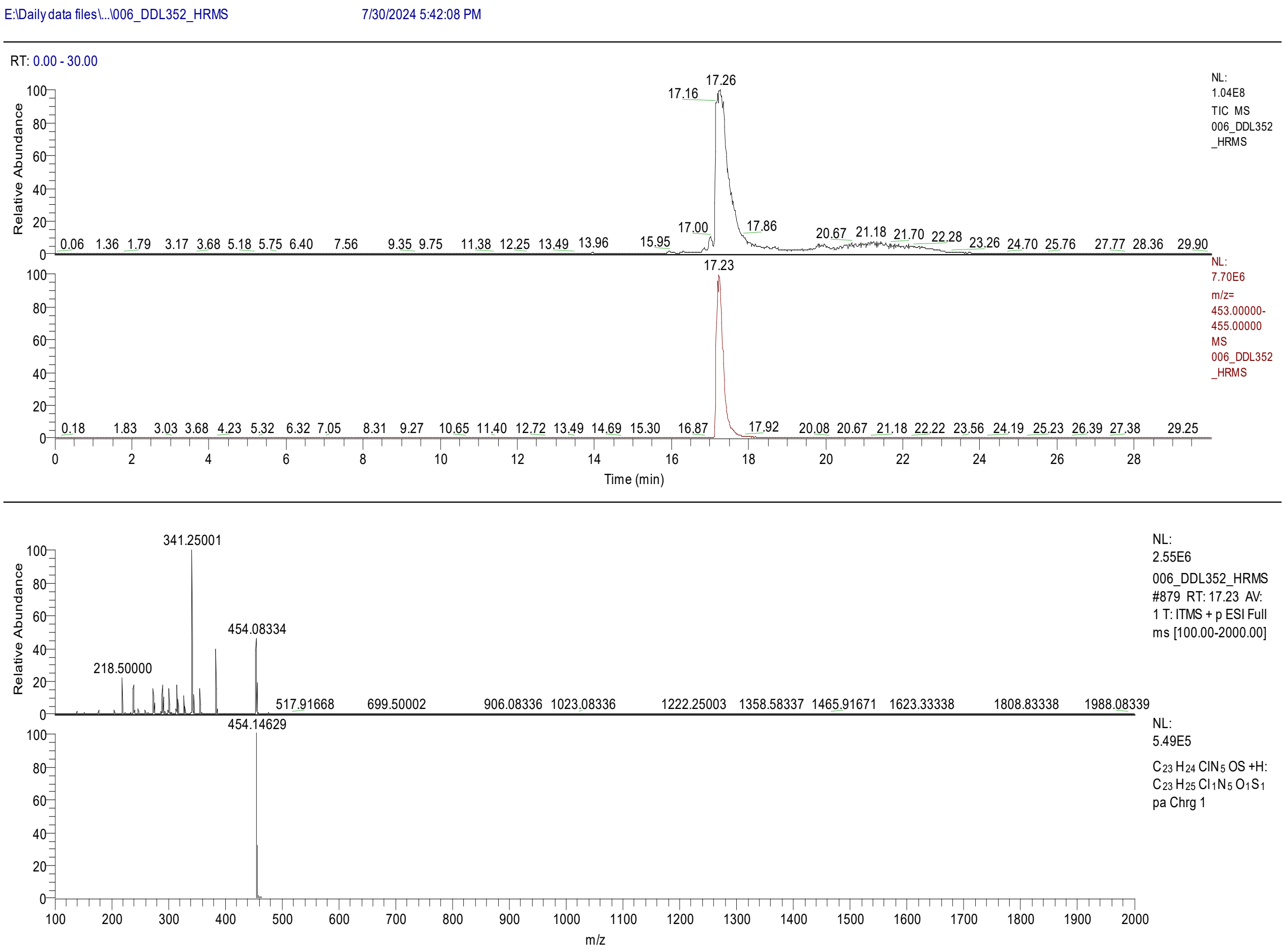

DDL-353:

(S)-2-(4-(4-chlorophenyl)-2,3,9-trimethyl-6H-thieno[3,2-f][1,2,4]triazolo[4,3-a][1,4]diazepin-6-yl)-N-cyclopentylacetamide

E:\Daily data files\...\008_DDL353_HRMS 7/30/2024 6:45:28 PM

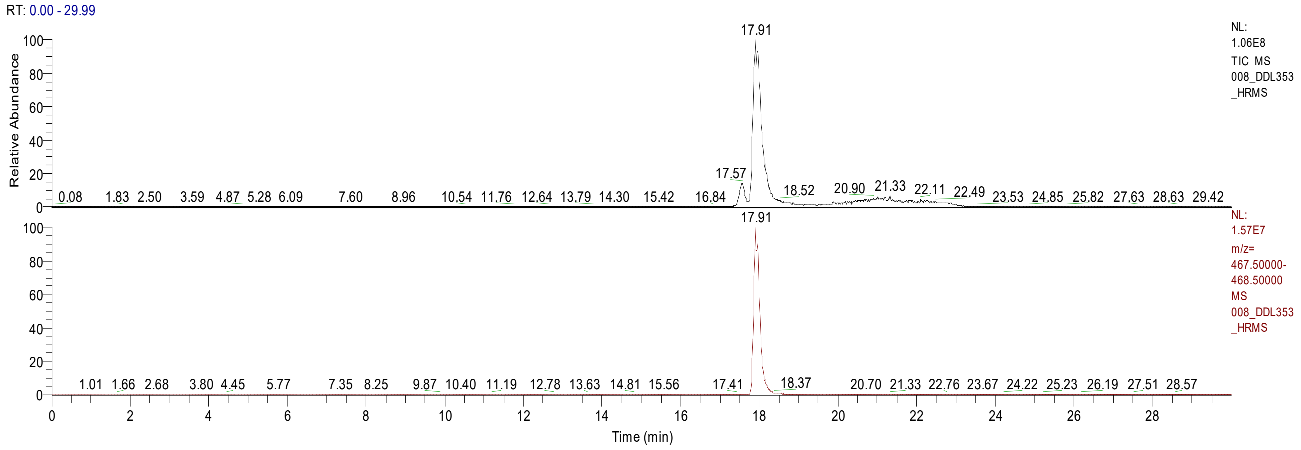

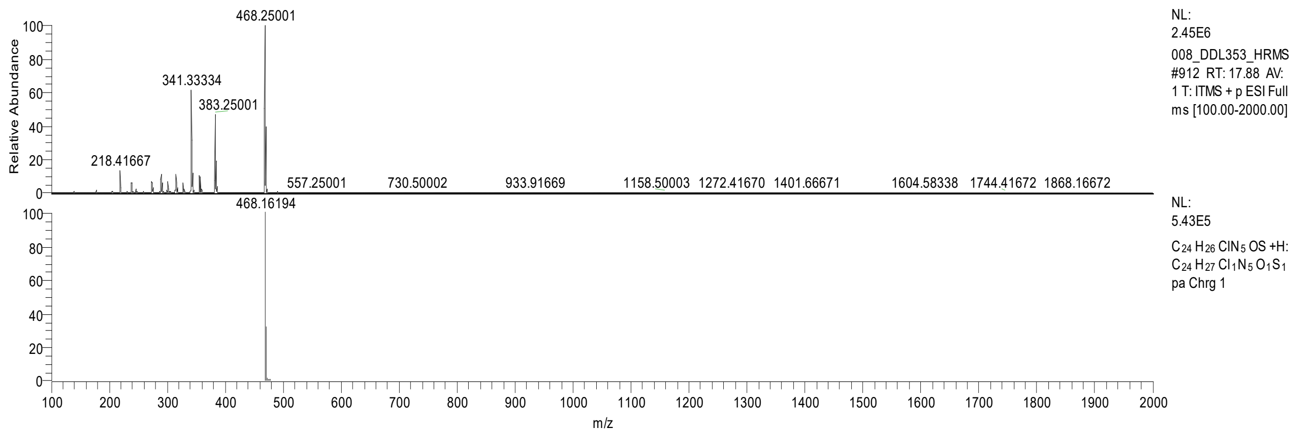

DDL-354:

Isopropyl (S)-(2-(4-(4-chlorophenyl)-2,3,9-trimethyl-6H-thieno[3,2-f][1,2,4]triazolo[4,3-a][1,4]diazepin-6-yl)acetyl)glycinate

010_DDL354_HRMS_Frac43 7/30/2024 7:48:53 PM

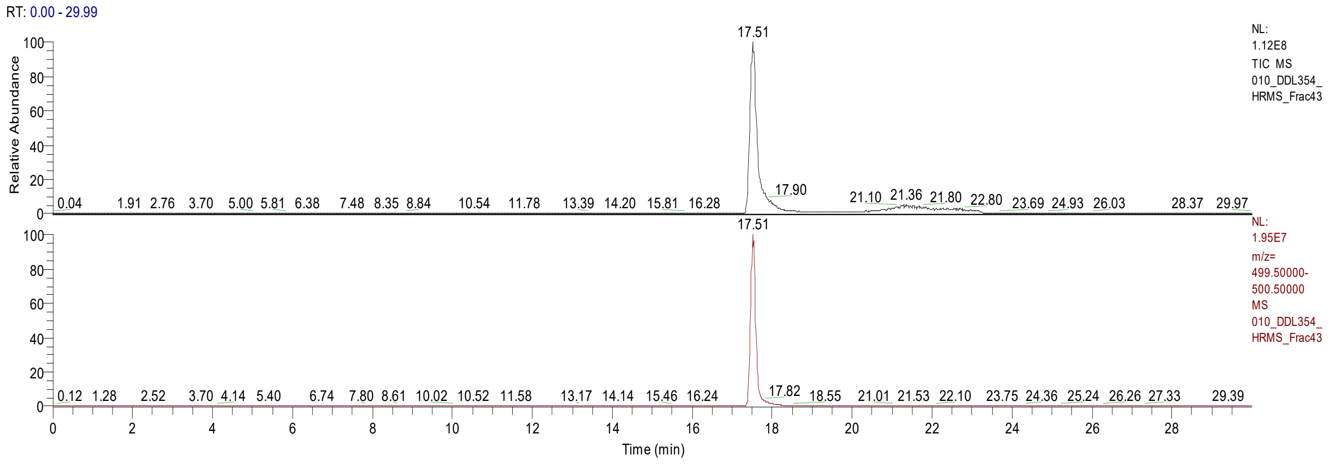

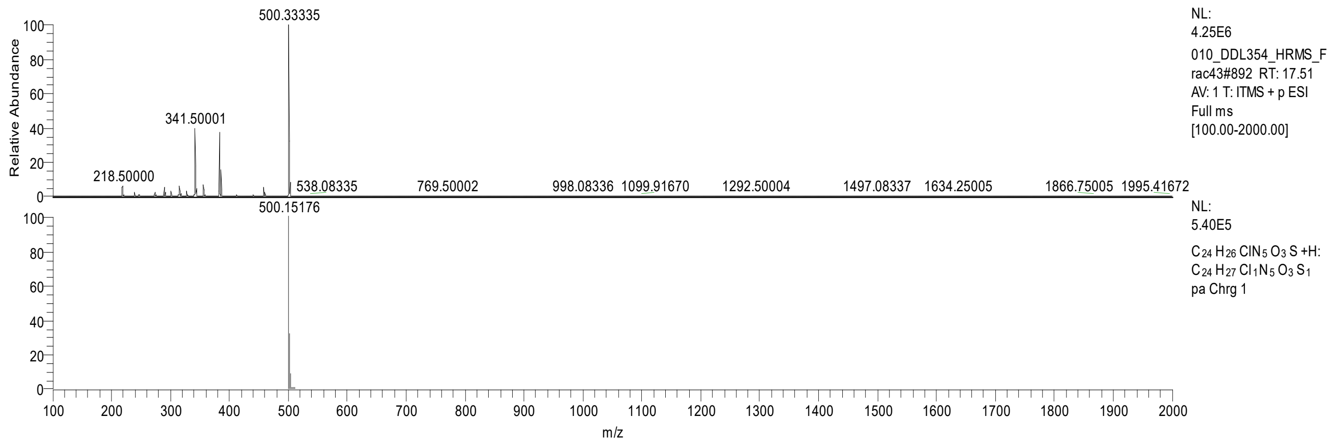

DDL 355:

Cyclobutyl (S)-2-(4-(4-chlorophenyl)-2,3,9-trimethyl-6H-thieno[3,2-f][1,2,4]triazolo[4,3-a][1,4]diazepin-6-yl)acetate

E:\Daily data files\...\012_DDL355_HRMS 7/30/2024 8:52:23 PM

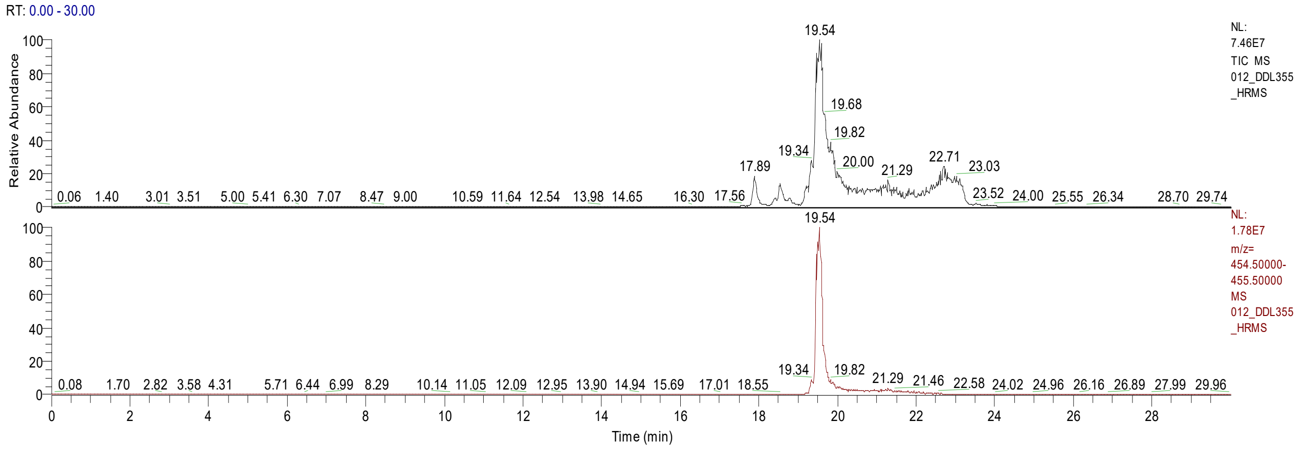

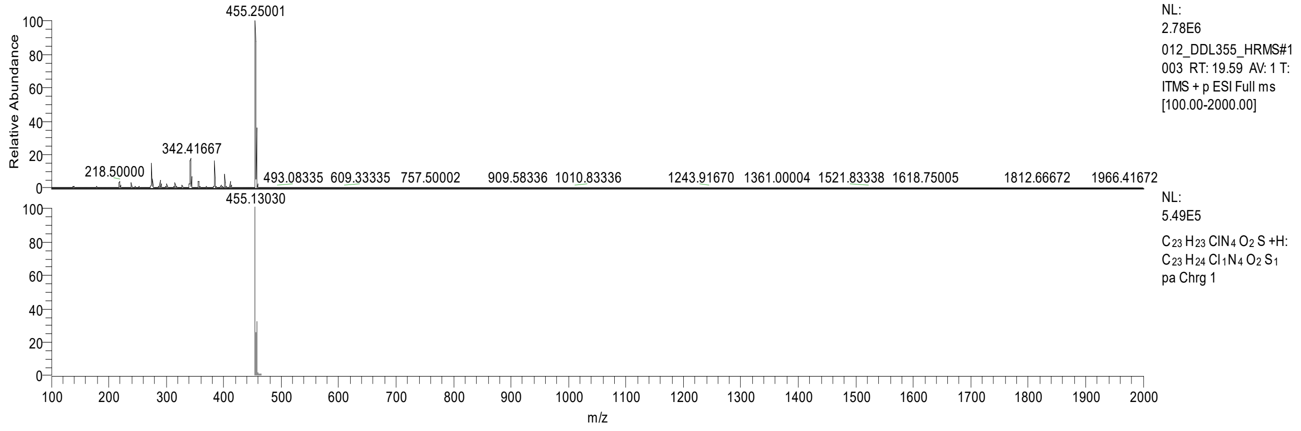

DDL 356:

Oxetan-3-yl (S)-2-(4-(4-chlorophenyl)-2,3,9-trimethyl-6H-thieno[3,2-f][1,2,4]triazolo[4,3-a][1,4]diazepin-6-yl)acetate

E:\Daily data files\...\014_DDL356_HRMS 7/30/2024 9:55:47 PM

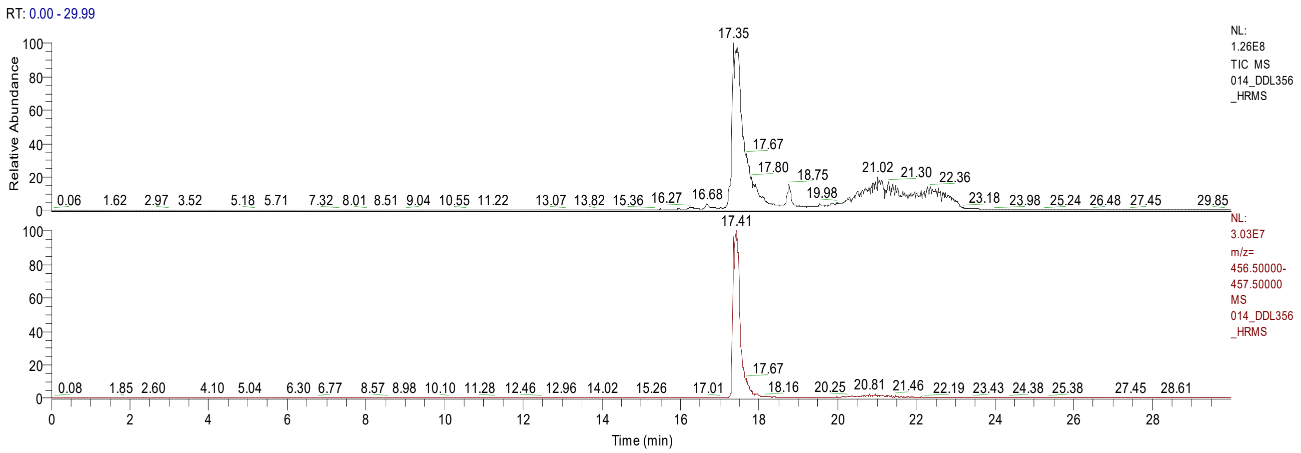

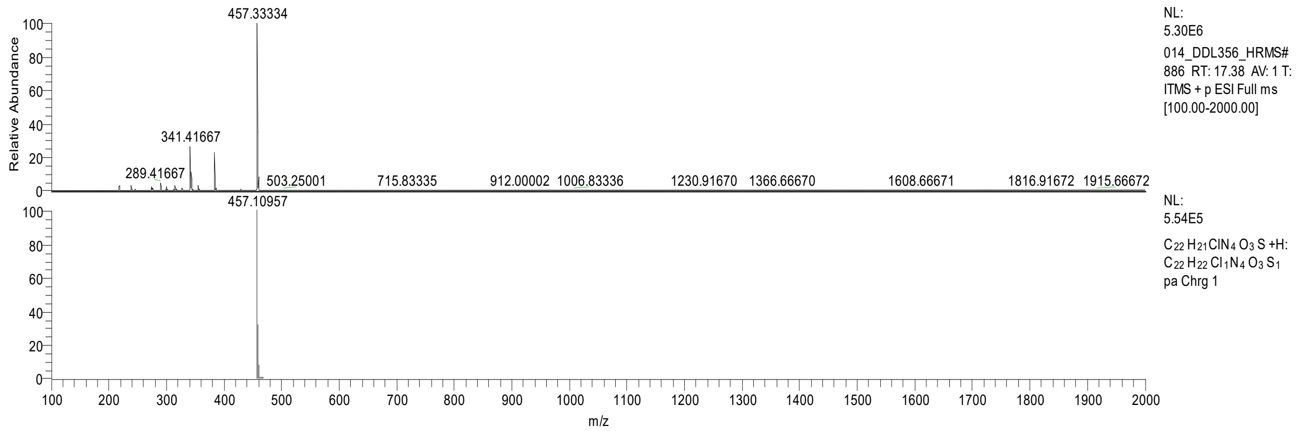

DDL-357:

Cyclopropyl (S)-2-(4-(4-chlorophenyl)-2,3,9-trimethyl-6H-thieno[3,2-f][1,2,4]triazolo[4,3-a][1,4]diazepin-6-yl)acetate

E:\Daily data files\...\016_DDL357_HRMS 7/30/2024 10:59:20 PM

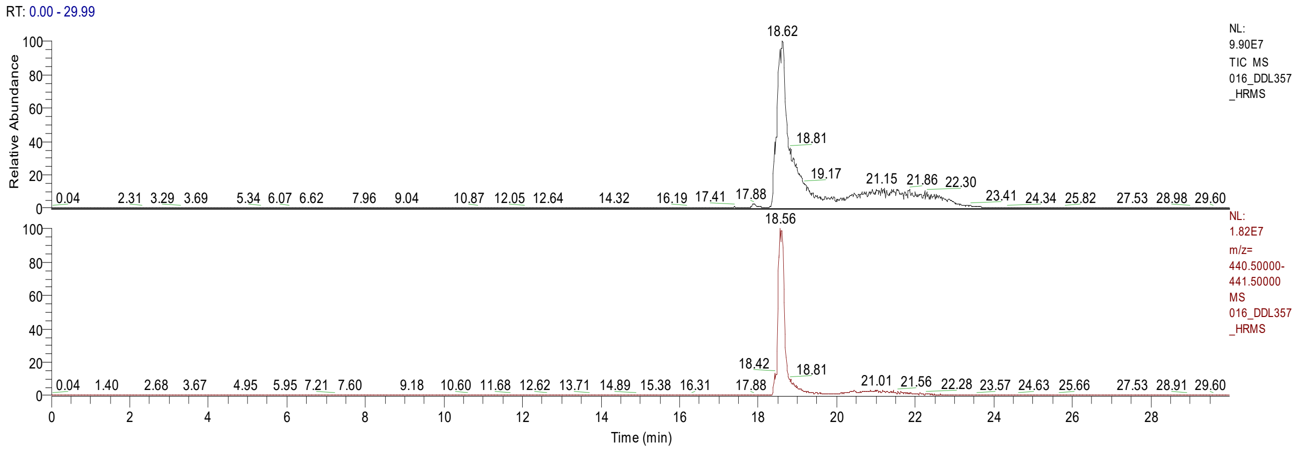

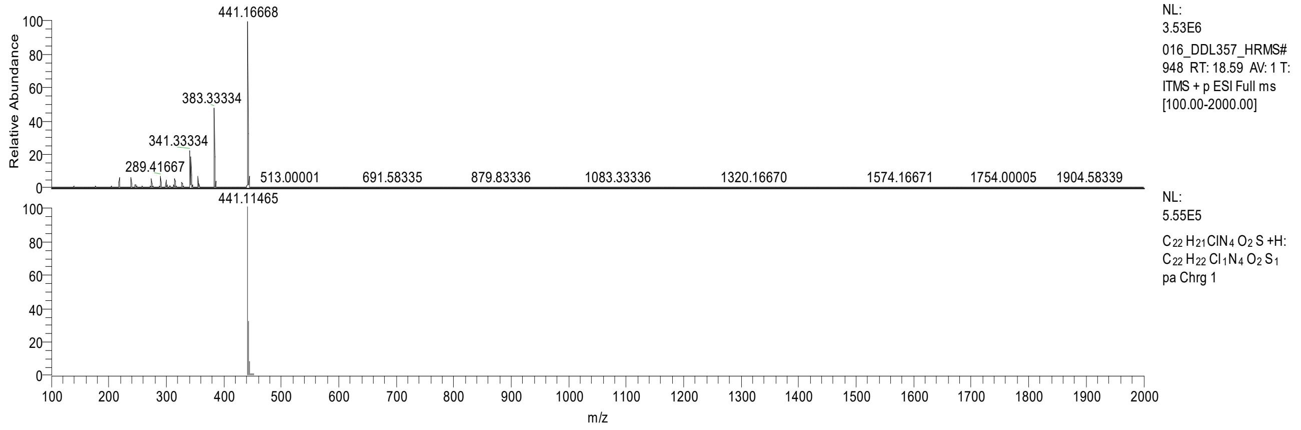

DDL-358:

Butyl (S)-2-(4-(4-chlorophenyl)-2,3,9-trimethyl-6H-thieno[3,2-f][1,2,4]triazolo[4,3-a][1,4]diazepin-6-yl)acetate

E:\Daily data files\...\018_DDL358_HRMS 7/31/2024 12:02:54 AM

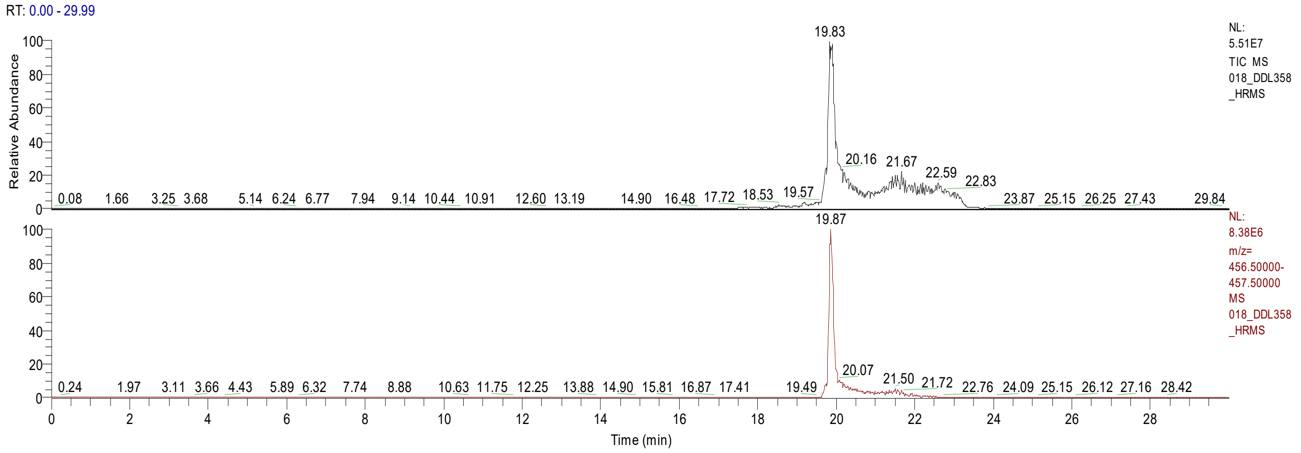

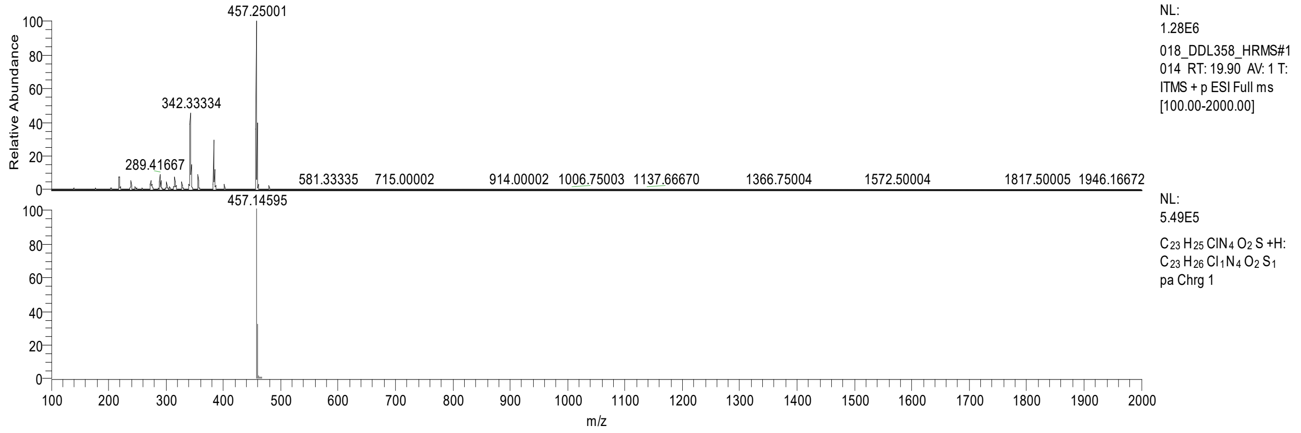

DDL-359:

Isopropyl (2-((S)-4-(4-chlorophenyl)-2,3,9-trimethyl-6H-thieno[3,2-f][1,2,4]triazolo[4,3-a][1,4]diazepin-6-yl)acetyl)-L-alaninate

030_DDL359_Frac42_HRMS 7/31/2024 4:31:44 PM

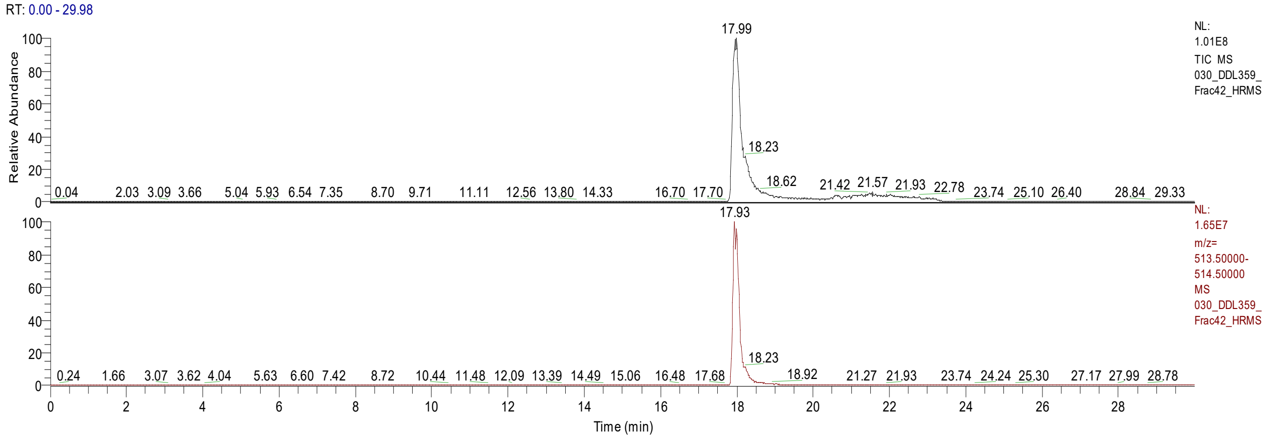

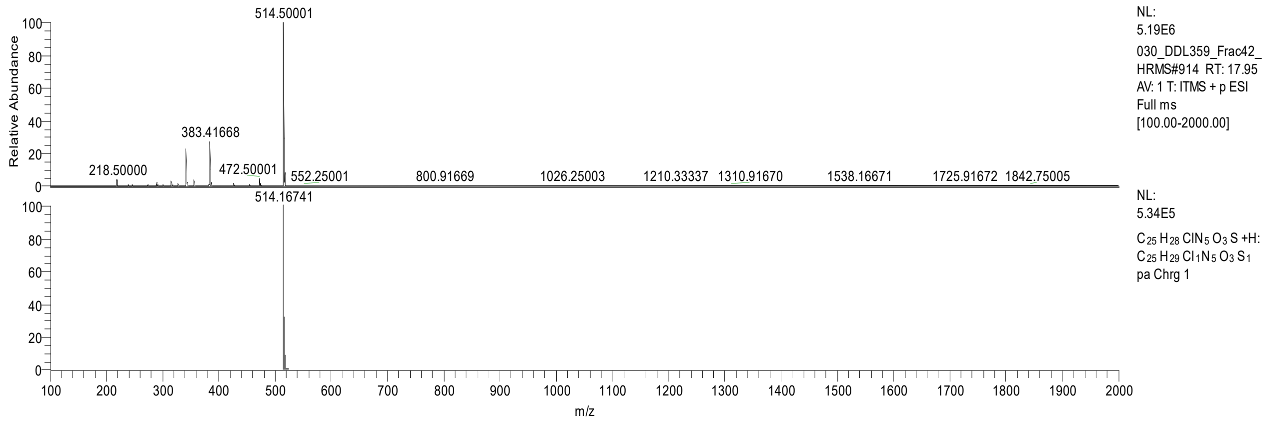

DDL-360:

(S)-2-(4-(4-chlorophenyl)-2,3,9-trimethyl-6H-thieno[3,2-f][1,2,4]triazolo[4,3-a][1,4]diazepin-6-yl)-N-hydroxyacetamide

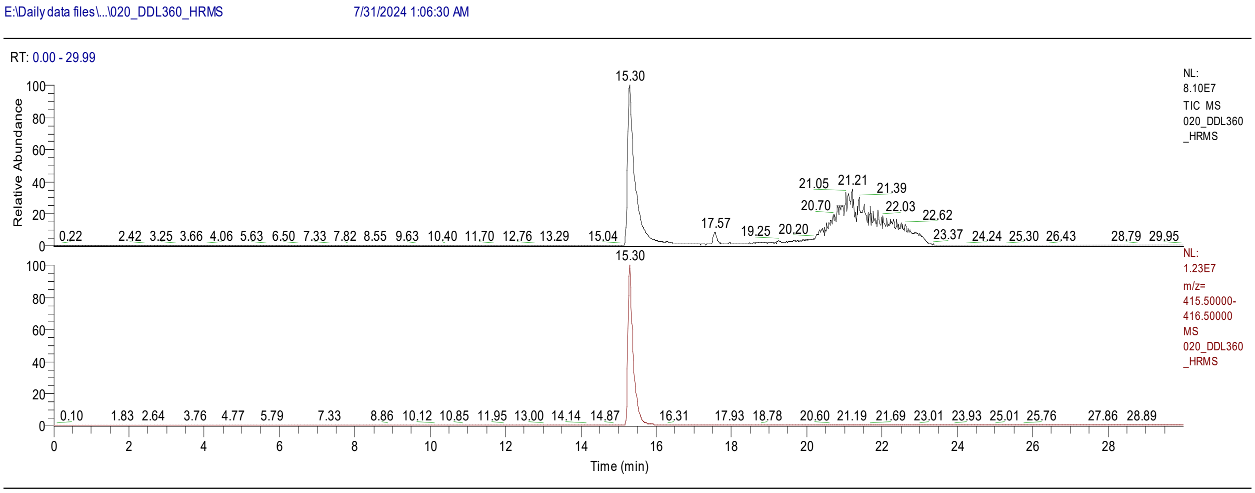

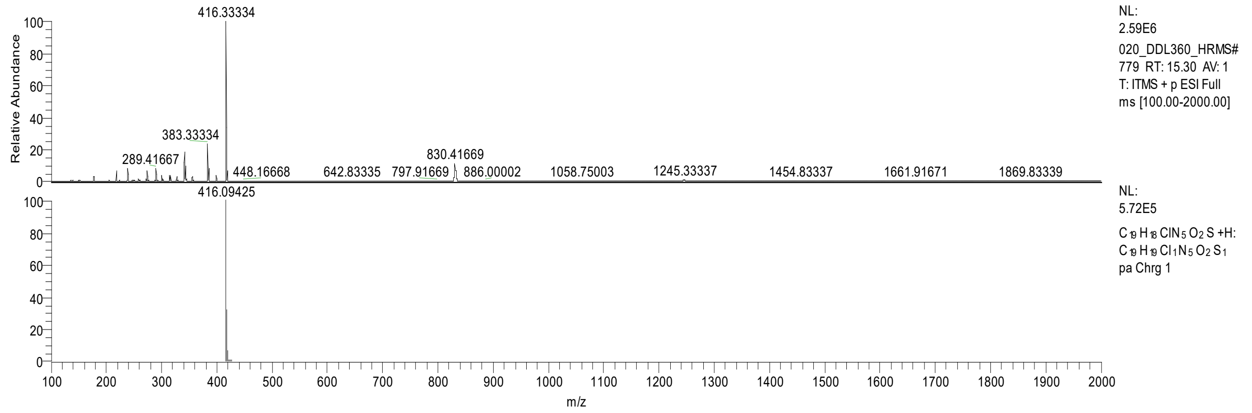

Supplementary References

1. Mamais A, Sanyal A, Fajfer A, Zykoski CG, Guldin M, Riley-DiPaolo A, Subrahmanian N, Gibbs W, Lin S, LaVoie MJ. The LRRK2 kinase substrates Rab8a and Rab10 contribute complementary but distinct disease-relevant phenotypes in human neurons. bioRxiv [Preprint]. 2023 Apr 30:2023.04.30.538317. doi: 10.1101/2023.04.30.538317. Update in: Stem Cell Reports. 2024 Feb 13;19(2):163-173. doi: 10.1016/j.stemcr.2024.01.001. PMID: 37163109; PMCID: PMC10168414.
2. Mamais A, Sanyal A, Fajfer A, Zykoski CG, Guldin M, Riley-DiPaolo A, Subrahmanian N, Gibbs W, Lin S, LaVoie MJ. The LRRK2 kinase substrates Rab8a and Rab10 contribute complementary but distinct disease-relevant phenotypes in human neurons. bioRxiv [Preprint]. 2023 Apr 30:2023.04.30.538317. doi: 10.1101/2023.04.30.538317. Update in: Stem Cell Reports. 2024 Feb 13;19(2):163-173. doi: 10.1016/j.stemcr.2024.01.001. PMID: 37163109; PMCID: PMC10168414.
3. Wong J. Altered expression of RNA splicing proteins in Alzheimer's disease patients: evidence from two microarray studies. Dement Geriatr Cogn Dis Extra. 2013 Mar 12;3(1):74-85. doi: 10.1159/000348406. PMID: 23637700; PMCID: PMC3617979.
4. Mori K, Nihei Y, Arzberger T, Zhou Q, Mackenzie IR, Hermann A, Hanisch F; German Consortium for Frontotemporal Lobar Degeneration; Bavarian Brain Banking Alliance; Kamp F, Nuscher B, Orozco D, Edbauer D, Haass C. Reduced hnRNPA3 increases C9orf72 repeat RNA levels and dipeptide-repeat protein deposition. EMBO Rep. 2016 Sep;17(9):1314-25. doi: 10.15252/embr.201541724. Epub 2016 Jul 26. PMID: 27461252; PMCID: PMC5007570.
5. Roberts JA, Varma VR, An Y, Varma S, Candia J, Fantoni G, Tiwari V, Anerillas C, Williamson A, Saito A, Loeffler T, Schilcher I, Moaddel R, Khadeer M, Lovett J, Tanaka T, Pletnikova O, Troncoso JC, Bennett DA, Albert MS, Yu K, Niu M, Haroutunian V, Zhang B, Peng J, Croteau DL, Resnick SM, Gorospe M, Bohr VA, Ferrucci L, Thambisetty M. A brain proteomic signature of incipient Alzheimer's disease in young APOE ε4 carriers identifies novel drug targets. Sci Adv. 2021 Nov 12;7(46):eabi8178. doi: 10.1126/sciadv.abi8178. Epub 2021 Nov 10. PMID: 34757788; PMCID: PMC8580310.
6. Song H, Kim W, Kim SH, Kim KT. VRK3-mediated nuclear localization of HSP70 prevents glutamate excitotoxicity-induced apoptosis and Aβ accumulation via enhancement of ERK phosphatase VHR activity. Sci Rep. 2016 Dec 12;6:38452. doi: 10.1038/srep38452. PMID: 27941812; PMCID: PMC5150261.
7. Bayraktar A, Lam S, Altay O, Li X, Yuan M, Zhang C, Arif M, Turkez H, Uhlén M, Shoaie S, Mardinoglu A. Revealing the Molecular Mechanisms of Alzheimer's Disease Based on Network Analysis. Int J Mol Sci. 2021 Oct 26;22(21):11556. doi: 10.3390/ijms222111556. PMID: 34768988; PMCID: PMC8584243.
8. Ilic K, Mlinac-Jerkovic K, Sedmak G, Rosenzweig I, Kalanj-Bognar S. Neuroplastin in human cognition: review of literature and future perspectives. Transl Psychiatry. 2021 Jul

16;11(1):394. doi: 10.1038/s41398-021-01509-1. PMID: 34282131; PMCID: PMC8289873.

9. Alfaro-Ruiz R, Martín-Belmonte A, Aguado C, Hernández F, Moreno-Martínez AE, Ávila J, Luján R. The Expression and Localisation of G-Protein-Coupled Inwardly Rectifying Potassium (GIRK) Channels Is Differentially Altered in the Hippocampus of Two Mouse Models of Alzheimer's Disease. Int J Mol Sci. 2021 Oct 14;22(20):11106. doi: 10.3390/ijms222011106. PMID: 34681766; PMCID: PMC8541655.
10. Kleschevnikov A. GIRK2 Channels in Down syndrome and Alzheimer's disease. Curr Alzheimer Res. 2022 Dec 23. doi: 10.2174/1567205020666221223122110. Epub ahead of print. PMID: 36567290.
11. Yang S, Du Y, Zhao X, Tang Q, Su W, Hu Y, Yu P. Cannabidiol Enhances Microglial Beta-Amyloid Peptide Phagocytosis and Clearance via Vanilloid Family Type 2 Channel Activation. Int J Mol Sci. 2022 May 11;23(10):5367. doi: 10.3390/ijms23105367. PMID: 35628181; PMCID: PMC9140666.
12. Li H, Gong W, Sun W, Yao Y, Han Y. Role of VPS39, a key tethering protein for endolysosomal trafficking and mitochondria-lysosome crosstalk, in health and disease. J Cell Biochem. 2023 Mar 16. doi: 10.1002/jcb.30396. Epub ahead of print. PMID: 36924104.
13. Babazadeh R, Ahmadpour D, Jia S, Hao X, Widlund P, Schneider K, Eisele F, Edo LD, Smits GJ, Liu B, Nystrom T. Syntaxin 5 Is Required for the Formation and Clearance of Protein Inclusions during Proteostatic Stress. Cell Rep. 2019 Aug 20;28(8):2096-2110.e8. doi: 10.1016/j.celrep.2019.07.053. PMID: 31433985.
14. Yin T, Yao W, Lemenze AD, D'Adamio L. Danish and British dementia ITM2b/BRI2 mutations reduce BRI2 protein stability and impair glutamatergic synaptic transmission. J Biol Chem. 2021 Jan-Jun;296:100054. doi: 10.1074/jbc.RA120.015679. Epub 2020 Nov 22. PMID: 33172889; PMCID: PMC7948410.
15. Faúndez V, Horng JT, Kelly RB. A function for the AP3 coat complex in synaptic vesicle formation from endosomes. Cell. 1998 May 1;93(3):423-32. doi: 10.1016/s0092-8674(00)81170-8. PMID: 9590176.
16. Shin J, Nile A, Oh JW. Role of adaptin protein complexes in intracellular trafficking and their impact on diseases. Bioengineered. 2021 Dec;12(1):8259-8278. doi: 10.1080/21655979.2021.1982846. PMID: 34565296; PMCID: PMC8806629.
17. Ramanan VK, Kim S, Holohan K, Shen L, Nho K, Risacher SL, Foroud TM, Mukherjee S, Crane PK, Aisen PS, Petersen RC, Weiner MW, Saykin AJ; Alzheimer's Disease Neuroimaging Initiative (ADNI). Genome-wide pathway analysis of memory impairment in the Alzheimer's Disease Neuroimaging Initiative (ADNI) cohort implicates gene candidates, canonical pathways, and networks. Brain Imaging Behav. 2012 Dec;6(4):634-48. doi: 10.1007/s11682-012-9196-x. PMID: 22865056; PMCID: PMC3713637.
18. Kourosh-Arami M, Komaki A, Joghataei MT, Mohsenzadegan M. Phospholipase Cβ3 in the hippocampus may mediate impairment of memory by long-term blockade of orexin 1

receptors assessed by the Morris water maze. Life Sci. 2020 Sep 15;257:118046. doi: 10.1016/j.lfs.2020.118046. Epub 2020 Jul 3. PMID: 32622948.
19. Wang XL, Li L. Cell type-specific potential pathogenic genes and functional pathways in Alzheimer's Disease. BMC Neurol. 2021 Oct 2;21(1):381. doi: 10.1186/s12883-021-02407-1. PMID: 34600516; PMCID: PMC8487122.
20. Zhang Y, Li D, Zeng Q, Feng J, Fu H, Luo Z, Xiao B, Yang H, Wu M. LRRC4 functions as a neuron-protective role in experimental autoimmune encephalomyelitis. Mol Med. 2021 May 1;27(1):44. doi: 10.1186/s10020-021-00304-4. PMID: 33932995; PMCID: PMC8088686.
21. Wang PY, Chang KT, Lin YM, Kuo TY, Wang GS. Ubiquitination of MBNL1 Is Required for Its Cytoplasmic Localization and Function in Promoting Neurite Outgrowth. Cell Rep. 2018 Feb 27;22(9):2294-2306. doi: 10.1016/j.celrep.2018.02.025. PMID: 29490267.
22. Sta Maria NS, Zhou C, Lee SJ, Valiulahi P, Li X, Choi J, Liu X, Jacobs R, Comai L, Reddy S. Mbnl1 and Mbnl2 regulate brain structural integrity in mice. Commun Biol. 2021 Nov 30;4(1):1342. doi: 10.1038/s42003-021-02845-0. PMID: 34848815; PMCID: PMC8633067.
23. 1. Lee AJ, Raghavan NS, Bhattarai P, Siddiqui T, Sariya S, Reyes-Dumeyer D, Flowers XE, Cardoso SAL, De Jager PL, Bennett DA, Schneider JA, Menon V, Wang Y, Lantigua RA, Medrano M, Rivera D, Jiménez-Velázquez IZ, Kukull WA, Brickman AM, Manly JJ, Tosto G, Kizil C, Vardarajan BN, Mayeux R. FMNL2 regulates gliovascular interactions and is associated with vascular risk factors and cerebrovascular pathology in Alzheimer's disease. Acta Neuropathol. 2022 Jul;144(1):59-79. doi: 10.1007/s00401-022-02431-6. Epub 2022 May 24. PMID: 35608697; PMCID: PMC9217776.
24. Ouellette AR, Neuner SM, Dumitrescu L, Anderson LC, Gatti DM, Mahoney ER, Bubier JA, Churchill G, Peters L, Huentelman MJ, Herskowitz JH, Yang HS, Smith AN, Reitz C, Kunkle BW, White CC, De Jager PL, Schneider JA, Bennett DA, Seyfried NT; Alzheimer's Disease Genetics Consortium; Chesler EJ, Hadad N, Hohman TJ, Kaczorowski CC. Cross-Species Analyses Identify Dlgap2 as a Regulator of Age-Related Cognitive Decline and Alzheimer's Dementia. Cell Rep. 2020 Sep 1;32(9):108091. doi: 10.1016/j.celrep.2020.108091. PMID: 32877673; PMCID: PMC7502175.
25. Hsieh MY, Tuan LH, Chang HC, Wang YC, Chen CH, Shy HT, Lee LJ, Gau SS. Altered synaptic protein expression, aberrant spine morphology, and impaired spatial memory in Dlgap2 mutant mice, a genetic model of autism spectrum disorder. Cereb Cortex. 2023 Apr 4;33(8):4779-4793. doi: 10.1093/cercor/bhac379. PMID: 36169576.
26. Bandura J, Feng ZP. Current Understanding of the Role of Neuronal Calcium Sensor 1 in Neurological Disorders. Mol Neurobiol. 2019 Sep;56(9):6080-6094. doi: 10.1007/s12035-019-1497-2. Epub 2019 Feb 4. PMID: 30719643.
27. Otera H, Wang C, Cleland MM, Setoguchi K, Yokota S, Youle RJ, Mihara K. Mff is an essential factor for mitochondrial recruitment of Drp1 during mitochondrial fission in

mammalian cells. J Cell Biol. 2010 Dec 13;191(6):1141-58. doi: 10.1083/jcb.201007152. PMID: 21149567; PMCID: PMC3002033.
28. Shin JH, Park SJ, Jo DS, Park NY, Kim JB, Bae JE, Jo YK, Hwang JJ, Lee JA, Jo DG, Kim JC, Jung YK, Koh JY, Cho DH. Down-regulated TMED10 in Alzheimer disease induces autophagy via ATG4B activation. Autophagy. 2019 Sep;15(9):1495-1505. doi: 10.1080/15548627.2019.1586249. Epub 2019 Mar 19. PMID: 30821607; PMCID: PMC6693468.
29. Li Q, Liu X, Xing R, Sui R. Transmembrane p24 trafficking protein 10 (TMED10) inhibits mitochondrial damage and protects neurons in ischemic stroke via the c-Jun N-terminal kinase (JNK) signaling pathway. Exp Anim. 2023 May 17;72(2):151-163. doi: 10.1538/expanim.22-0060. Epub 2022 Oct 21. PMID: 36244749; PMCID: PMC10202712.
30. Trumpff C, Owusu-Ansah E, Klein HU, Lee AJ, Petyuk V, Wingo TS, Wingo AP, Thambisetty M, Ferrucci L, Seyfried NT, Bennett DA, De Jager PL, Picard M. Mitochondrial respiratory chain protein co-regulation in the human brain. Heliyon. 2022 Apr 30;8(5):e09353. doi: 10.1016/j.heliyon.2022.e09353. PMID: 35600441; PMCID: PMC9118667.
31. Chen F, Wang N, He X. Identification of Differential Genes of DNA Methylation Associated With Alzheimer's Disease Based on Integrated Bioinformatics and Its Diagnostic Significance. Front Aging Neurosci. 2022 May 9;14:884367. doi: 10.3389/fnagi.2022.884367. PMID: 35615586; PMCID: PMC9125150.
32. Cuttler K, Bignoux MJ, Otgaar TC, Chigumba S, Ferreira E, Weiss SFT. LRP::FLAG Reduces Phosphorylated Tau Levels in Alzheimer's Disease Cell Culture Models. J Alzheimers Dis. 2020;76(2):753-768. doi: 10.3233/JAD-200244. PMID: 32568204.
33. Blazejewski SM, Bennison SA, Ha NT, Liu X, Smith TH, Dougherty KJ, Toyo-Oka K. Rpsa Signaling Regulates Cortical Neuronal Morphogenesis via Its Ligand, PEDF, and Plasma Membrane Interaction Partner, Itga6. Cereb Cortex. 2022 Feb 8;32(4):770-795. doi: 10.1093/cercor/bhab242. PMID: 34347028; PMCID: PMC8841558.
34. O'Reilly J, Pangilinan F, Hokamp K, Ueland PM, Brosnan JT, Brosnan ME, Brody LC, Molloy AM. The impact of common genetic variants in the mitochondrial glycine cleavage system on relevant metabolites. Mol Genet Metab Rep. 2018 Jun 11;16:20-22. doi: 10.1016/j.ymgmr.2018.05.006. PMID: 29988937; PMCID: PMC6034155.
35. Chen J, Dai AX, Tang HL, Lu CH, Liu HX, Hou T, Lu ZJ, Kong N, Peng XY, Lin KX, Zheng ZD, Xu SL, Ying XF, Ji XY, Pan H, Wu J, Zeng X, Wei NL. Increase of ALCAM and VCAM-1 in the plasma predicts the Alzheimer's disease. Front Immunol. 2023 Jan 4;13:1097409. doi: 10.3389/fimmu.2022.1097409. PMID: 36685605; PMCID: PMC9846483.
36. Bye CR, Rytova V, Alsanie WF, Parish CL, Thompson LH. Axonal Growth of Midbrain Dopamine Neurons is Modulated by the Cell Adhesion Molecule ALCAM Through Trans-Heterophilic Interactions with L1cam, Chl1, and Semaphorins. J

Neurosci. 2019 Aug 21;39(34):6656-6667. doi: 10.1523/JNEUROSCI.0278-19.2019. Epub 2019 Jul 12. PMID: 31300520; PMCID: PMC6703882.
37. Dekker FA, Rüdiger SGD. The Mitochondrial Hsp90 TRAP1 and Alzheimer's Disease. Front Mol Biosci. 2021 Jun 18;8:697913. doi: 10.3389/fmolb.2021.697913. PMID: 34222342; PMCID: PMC8249562.
38. Ramos Rego I, Santos Cruz B, Ambrósio AF, Alves CH. TRAP1 in Oxidative Stress and Neurodegeneration. Antioxidants (Basel). 2021 Nov 19;10(11):1829. doi: 10.3390/antiox10111829. PMID: 34829705; PMCID: PMC8614808.
39. Vardarajan BN, Bruesegem SY, Harbour ME, Inzelberg R, Friedland R, St George-Hyslop P, Seaman MN, Farrer LA. Identification of Alzheimer disease-associated variants in genes that regulate retromer function. Neurobiol Aging. 2012 Sep;33(9):2231.e15-2231.e30. doi: 10.1016/j.neurobiolaging.2012.04.020. Epub 2012 Jun 5. Erratum in: Neurobiol Aging. 2013 Jul;34(7):1923. Inzelberg, Rivka [added]; Friedland, Robert [added]. PMID: 22673115; PMCID: PMC3391348.
40. Zhang H, Huang T, Hong Y, Yang W, Zhang X, Luo H, Xu H, Wang X. The Retromer Complex and Sorting Nexins in Neurodegenerative Diseases. Front Aging Neurosci. 2018 Mar 26;10:79. doi: 10.3389/fnagi.2018.00079. PMID: 29632483; PMCID: PMC5879135.
41. Nielsen MS, Gustafsen C, Madsen P, Nyengaard JR, Hermey G, Bakke O, Mari M, Schu P, Pohlmann R, Dennes A, Petersen CM. Sorting by the cytoplasmic domain of the amyloid precursor protein binding receptor SorLA. Mol Cell Biol. 2007 Oct;27(19):6842-51. doi: 10.1128/MCB.00815-07. Epub 2007 Jul 23. PMID: 17646382; PMCID: PMC2099242.
42. Farhadieh ME, Ghaedi K. Analyzing alternative splicing in Alzheimer's disease postmortem brain: a cell-level perspective. Front Mol Neurosci. 2023 Sep 20;16:1237874. doi: 10.3389/fnmol.2023.1237874. PMID: 37799732; PMCID: PMC10548223.
43. Xu B, Gao C, Zhang H, Huang X, Yang X, Yang C, Liu W, Wu D, Liu J. A quantitative proteomic analysis reveals the potential roles of PRDX3 in neurite outgrowth in N2a-APPswe cells. Biochem Biophys Res Commun. 2022 May 14;604:144-150. doi: 10.1016/j.bbrc.2022.03.021. Epub 2022 Mar 5. PMID: 35303681.
44. Chen L, Na R, Ran Q. Enhanced defense against mitochondrial hydrogen peroxide attenuates age-associated cognition decline. Neurobiol Aging. 2014 Nov;35(11):2552-2561. doi: 10.1016/j.neurobiolaging.2014.05.007. Epub 2014 May 10. PMID: 24906890.
45. Lau DHW, Paillusson S, Hartopp N, Rupawala H, Mórotz GM, Gomez-Suaga P, Greig J, Troakes C, Noble W, Miller CCJ. Disruption of endoplasmic reticulum-mitochondria tethering proteins in post-mortem Alzheimer's disease brain. Neurobiol Dis. 2020 Sep;143:105020. doi: 10.1016/j.nbd.2020.105020. Epub 2020 Jul 17. PMID: 32682953; PMCID: PMC7794060.
46. Han KA, Ko J. Orchestration of synaptic functions by WAVE regulatory complex-mediated actin reorganization. Exp Mol Med. 2023 Jun;55(6):1065-1075. doi:

10.1038/s12276-023-01004-1. Epub 2023 Jun 1. PMID: 37258575; PMCID: PMC10318009.
47. Li Q, Li X, Wang L, Zhang Y, Chen L. miR-98-5p Acts as a Target for Alzheimer's Disease by Regulating Aβ Production Through Modulating SNX6 Expression. J Mol Neurosci. 2016 Dec;60(4):413-420. doi: 10.1007/s12031-016-0815-7. Epub 2016 Aug 19. PMID: 27541017.
48. Okada H, Zhang W, Peterhoff C, Hwang JC, Nixon RA, Ryu SH, Kim TW. Proteomic identification of sorting nexin 6 as a negative regulator of BACE1-mediated APP processing. FASEB J. 2010 Aug;24(8):2783-94. doi: 10.1096/fj.09-146357. Epub 2010 Mar 30. PMID: 20354142; PMCID: PMC2909280.
49. Wei CJ, Cui P, Li H, Lang WJ, Liu GY, Ma XF. Shared genes between Alzheimer's disease and ischemic stroke. CNS Neurosci Ther. 2019 Aug;25(8):855-864. doi: 10.1111/cns.13117. Epub 2019 Mar 11. PMID: 30859738; PMCID: PMC6630005.
50. Ambivero CT, Cilenti L, Main S, Zervos AS. Mulan E3 ubiquitin ligase interacts with multiple E2 conjugating enzymes and participates in mitophagy by recruiting GABARAP. Cell Signal. 2014 Dec;26(12):2921-9. doi: 10.1016/j.cellsig.2014.09.004. Epub 2014 Sep 16. PMID: 25224329.
51. Zhang X, Huo C, Liu Y, Su R, Zhao Y, Li Y. Mechanism and Disease Association With a Ubiquitin Conjugating E2 Enzyme: UBE2L3. Front Immunol. 2022 Feb 21;13:793610. doi: 10.3389/fimmu.2022.793610. PMID: 35265070; PMCID: PMC8899012.
52. Xiong J, Pang X, Song X, Yang L, Pang C. The coherence between PSMC6 and α-ring in the 26S proteasome is associated with Alzheimer's disease. Front Mol Neurosci. 2024 Jan 31;16:1330853. doi: 10.3389/fnmol.2023.1330853. PMID: 38357597; PMCID: PMC10864545.
53. Fedele E, Ricciarelli R. Memory Enhancers for Alzheimer's Dementia: Focus on cGMP. Pharmaceuticals (Basel). 2021 Jan 13;14(1):61. doi: 10.3390/ph14010061. PMID: 33451088; PMCID: PMC7828493.
54. Correia SS, Iyengar RR, Germano P, Tang K, Bernier SG, Schwartzkopf CD, Tobin J, Lee TW, Liu G, Jacobson S, Carvalho A, Rennie GR, Jung J, Renhowe PA, Lonie E, Winrow CJ, Hadcock JR, Jones JE, Currie MG. The CNS-Penetrant Soluble Guanylate Cyclase Stimulator CY6463 Reveals its Therapeutic Potential in Neurodegenerative Diseases. Front Pharmacol. 2021 May 24;12:656561. doi: 10.3389/fphar.2021.656561. PMID: 34108877; PMCID: PMC8181742.
55. Puthiyedth N, Riveros C, Berretta R, Moscato P. Identification of Differentially Expressed Genes through Integrated Study of Alzheimer's Disease Affected Brain Regions. PLoS One. 2016 Apr 6;11(4):e0152342. doi: 10.1371/journal.pone.0152342. PMID: 27050411; PMCID: PMC4822961.
56. Chen S, He R, Lin X, Zhang W, Chen H, Xu R, Kang M. PTMA binds to HMGB1 to regulate mitochondrial oxidative phosphorylation and thus affect the malignant progression of esophageal squamous cell carcinoma. J Thorac Dis. 2023 Mar

31;15(3):1302-1318. doi: 10.21037/jtd-23-143. Epub 2023 Mar 29. PMID: 37065565; PMCID: PMC10089875.

57. Ueda H, Sasaki K, Halder SK, Deguchi Y, Takao K, Miyakawa T, Tajima A. Prothymosin alpha-deficiency enhances anxiety-like behaviors and impairs learning/memory functions and neurogenesis. J Neurochem. 2017 Apr;141(1):124-136. doi: 10.1111/jnc.13963. Epub 2017 Feb 21. PMID: 28122138.
58. Nelson TJ, Cui C, Luo Y, Alkon DL. Reduction of beta-amyloid levels by novel protein kinase C(epsilon) activators. J Biol Chem. 2009 Dec 11;284(50):34514-21. doi: 10.1074/jbc.M109.016683. Epub 2009 Oct 22. PMID: 19850930; PMCID: PMC2787312.
59. Choi DS, Wang D, Yu GQ, Zhu G, Kharazia VN, Paredes JP, Chang WS, Deitchman JK, Mucke L, Messing RO. PKCepsilon increases endothelin converting enzyme activity and reduces amyloid plaque pathology in transgenic mice. Proc Natl Acad Sci U S A. 2006 May 23;103(21):8215-20. doi: 10.1073/pnas.0509725103. Epub 2006 May 12. PMID: 16698938; PMCID: PMC1472455.
60. Aksenov MY, Aksenova MV, Payne RM, Smith CD, Markesbery WR, Carney JM. The expression of creatine kinase isoenzymes in neocortex of patients with neurodegenerative disorders: Alzheimer's and Pick's disease. Exp Neurol. 1997 Aug;146(2):458-65. doi: 10.1006/exnr.1997.6550. PMID: 9270056.
61. Lin YS, Cheng TH, Chang CP, Chen HM, Chern Y. Enhancement of brain-type creatine kinase activity ameliorates neuronal deficits in Huntington's disease. Biochim Biophys Acta. 2013 Jun;1832(6):742-53. doi: 10.1016/j.bbadis.2013.02.006. Epub 2013 Feb 15. PMID: 23416527.
62. Farid MM, Yang X, Kuboyama T, Tohda C. Trigonelline recovers memory function in Alzheimer's disease model mice: evidence of brain penetration and target molecule. Sci Rep. 2020 Oct 2;10(1):16424. doi: 10.1038/s41598-020-73514-1. PMID: 33009465; PMCID: PMC7532147.
63. Beck SJ, Guo L, Phensy A, Tian J, Wang L, Tandon N, Gauba E, Lu L, Pascual JM, Kroener S, Du H. Deregulation of mitochondrial F1FO-ATP synthase via OSCP in Alzheimer's disease. Nat Commun. 2016 May 6;7:11483. doi: 10.1038/ncomms11483. PMID: 27151236; PMCID: PMC5494197.
64. Gauba E, Sui S, Tian J, Driskill C, Jia K, Yu C, Rughwani T, Wang Q, Kroener S, Guo L, Du H. Modulation of OSCP mitigates mitochondrial and synaptic deficits in a mouse model of Alzheimer's pathology. Neurobiol Aging. 2021 Feb;98:63-77. doi: 10.1016/j.neurobiolaging.2020.09.018. Epub 2020 Oct 16. PMID: 33254080; PMCID: PMC7923248.

65. Zho Z, Bai J, Zhong S, Zhang R, Kang K, Zhang X, Xu Y, Zhao C, Zhao M. Integrative genomic analysis of PPP3R1 in AD: aa potential biomarker for predictive, preventative,

and personalized medical approach. EPMA Journal, 2021, 15, 647-658. doi.org/10.1007/s13167-021-00261-2. PMID: 34956428.

66. Chung D, Shum A, Caraveo G. GAP-43 and BASP1 in Axon Regeneration: Implications for treatment of Neurodegenerative Diseases. Fron Cell Dev Biol., 2020, 8: 567537. PMID:33015061, doi:10.3389/fcell.2020.567537.

67. Thelen K, Maier B, Faber M, Albrecht C, Fischer P, Pollerberg GE. Translation of the cell adhesion molecule ALCAM in axonal growth cones - regulation and functional importance. J Cell Sci. 2012 Feb 15;125(Pt 4):1003-14. doi: 10.1242/jcs.096149. Epub 2012 Mar 15. PMID: 22421359.\

68. Chen J, Dai AX, Tang HL, Lu CH, Liu HX, Hou T, Lu ZJ, Kong N, Peng XY, Lin KX, Zheng ZD, Xu SL, Ying XF, Ji XY, Pan H, Wu J, Zeng X, Wei NL. Increase of ALCAM and VCAM-1 in the plasma predicts the Alzheimer's disease. Front Immunol. 2023 Jan 4;13:1097409. doi: 10.3389/fimmu.2022.1097409. PMID: 36685605; PMCID: PMC9846483.

69. Jakob B, Kochlamazashvili G, Jäpel M, Gauhar A, Bock HH, Maritzen T, Haucke V. Intersectin 1 is a component of the Reelin pathway to regulate neuronal migration and synaptic plasticity in the hippocampus. Proc Natl Acad Sci U S A. 2017 May 23;114(21):5533-5538. doi: 10.1073/pnas.1704447114. Epub 2017 May 8. PMID: 28484035; PMCID: PMC5448185.

70. Malakooti N, Pritchard MA, Chen F, Yu Y, Sgambelloni C, Adlard PA, Finkelstein DI. The Long Isoform of Intersectin-1 Has a Role in Learning and Memory. Front Behav Neurosci. 2020 Feb 25;14:24. doi: 10.3389/fnbeh.2020.00024. PMID: 32161523; PMCID: PMC7052523.

71. Yan W, Fan J, Zhang X, Song H, Wan R, Wang W, Yin Y. Decreased neuronal synaptosome associated protein 29 contributes to poststroke cognitive impairment by disrupting presynaptic maintenance. Theranostics. 2021 Mar 4;11(10):4616-4636. doi: 10.7150/thno.54210. PMID: 33754017; PMCID: PMC7978312.

72. Hiesberger T, Trommsdorff M, Howell BW, Goffinet A, Mumby MC, Cooper JA, Herz J. Direct binding of Reelin to VLDL receptor and ApoE receptor 2 induces tyrosine phosphorylation of disabled-1 and modulates tau phosphorylation. Neuron. 1999 Oct;24(2):481-9. doi: 10.1016/s0896-6273(00)80861-2. PMID: 10571241.

73. Chen XY, Xu F, Chen JQ, Landeck L, Chen SQ, Zhou Y, Wang Z, Fu NC, Zheng M, Zhang X, Man XY. UBE2L3 Reduces TRIM21 Expression and IL-1β Secretion in Epidermal Keratinocytes and Improves Psoriasis-Like Skin. J Invest Dermatol. 2023 May;143(5):822-831.e4. doi: 10.1016/j.jid.2022.10.016. Epub 2022 Dec 9. PMID: 36502938.

74. Zhang Z, Klionsky DJ. CCT2, a newly identified aggrephagy receptor in mammals, specifically mediates the autophagic clearance of solid protein aggregates. Autophagy. 2022 Jul;18(7):1483-1485. doi: 10.1080/15548627.2022.2083305. Epub 2022 Jun 14. PMID: 35699934; PMCID: PMC9298431.
